# An exploration of the causal relationship between 731 immunophenotypes and osteoporosis: a bidirectional Mendelian randomized study

**DOI:** 10.3389/fendo.2024.1341002

**Published:** 2024-07-17

**Authors:** Dongqi Zhou, Changyan Zi, Gaofeng Gan, Shiyun Tang, Qiu Chen

**Affiliations:** ^1^ Department of Traditional Chinese Medicine, Sichuan Taikang Hospital, Chengdu, Sichuan, China; ^2^ Department of Good Clinical Practice (GCP), Hospital of Chengdu University of Traditional Chinese Medicine, Chengdu, Sichuan, China; ^3^ Department of Endocrine, Hospital of Chengdu University of Traditional Chinese Medicine, Chengdu, Sichuan, China

**Keywords:** osteoporosis, immunocyte phenotype, mendelian randomization, causal connection, B cells

## Abstract

**Background:**

There are complex interactions between osteoporosis and the immune system, and it has become possible to explore their causal relationship based on Mendelian randomization methods.

**Methods:**

Utilizing openly accessible genetic data and employing Mendelian randomization analysis, we investigated the potential causal connection between 731 immune cell traits and the risk of developing osteoporosis.

**Results:**

Ten immune cell phenotypes were osteoporosis protective factors and three immune cell phenotypes were osteoporosis risk factors. Specifically, the odds ratio (OR) of IgD+ CD24+ %B cell (B cell panel) risk on Osteoporosis was estimated to be 0.9986 (95% CI = 0.9978~0.9996, P<0.01). The OR of CD24+ CD27+ %B cell (B cell panel) risk on Osteoporosis was estimated to be 0.9991 (95% CI = 0.9984~0.9998, P = 0.021). The OR of CD33- HLA DR+AC (Myeloid cell panel) risk on Osteoporosis was estimated to be 0.9996 (95% CI = 0.9993~0.9999, P = 0.038). The OR of EM CD8br %CD8br (Maturation stages of T cell panel) risk on Osteoporosis was estimated to be 1.0004 (95% CI = 1.0000~1.0008, P = 0.045). The OR of CD25 on IgD+ (B cell panel) risk on Osteoporosis was estimated to be 0.9995 (95% CI = 0.9991~0.9999, P = 0.024). The OR of CD25 on CD39+ activated Treg+ (Treg panel) risk on Osteoporosis was estimated to be 1.001 (95% CI = 1.0001~1.0019, P = 0.038). The OR of CCR2 on CD62L+ myeloid DC (cDC panel) risk on Osteoporosis was estimated to be 0.9992 (95% CI = 0.9984~0.9999, P = 0.048). The OR of CCR2 on CD62L+ plasmacytoid DC (cDC panel) risk on Osteoporosis was estimated to be 0.9993 (95% CI = 0.9987~0.9999, P = 0.035). The OR of CD45 on CD33dim HLA DR+ CD11b- (Myeloid cell panel) risk on Osteoporosis was estimated to be 0.9988 (95% CI = 0.9977~0.9998, P = 0.031). The OR of CD45 on Mo MDSC (Myeloid cell panel) risk on Osteoporosis was estimated to be 0.9992 (95% CI = 0.9985~0.9998, P = 0.017). The OR of SSC-A on B cell (TBNK panel) risk on Osteoporosis was estimated to be 0.9986 (95% CI = 0.9972~0.9999, P = 0.042). The OR of CD11c on CD62L+ myeloid DC (cDC panel) risk on Osteoporosis was estimated to be 0.9987 (95% CI = 0.9978~0.9996, P<0.01). The OR of HLA DR on DC (cDC panel) risk on Osteoporosis was estimated to be 1.0007 (95% CI = 1.0002~1.0011, P<0.01). No causal effect of osteoporosis on immune cells was observed.

**Conclusions:**

Our study identified 13 unreported immune phenotypes that are causally related to osteoporosis, providing a theoretical basis for the bone immunology doctrine.

## 1 Introduction

Osteoporosis is a metabolic bone disease characterized by changes in the microstructure of bones, decreased bone density, and increased bone fragility (1). In recent years, the complex interaction between the immune system and osteoporosis has received attention. With the introduction of the concept of “immunoporosis” (2), the correlation between immune cells and osteoporosis has become a research hotspot.

A multitude of research indicates that the immune system, encompassing both its innate and adaptive branches, is critically involved in both the onset and persistence of the inflammatory state characteristic of osteoporosis (3–5). Cells of the immune system, along with an array of chemokines and cytokines, affect the metabolic dynamics of bones, influencing the growth, differentiation, and equilibrium of osteoclasts and osteoblasts (6, 7). Bone marrow macrophages (BMMs) can switch between M1 inflammatory state and M2 repair state depending on the surrounding environment (8). Research reports that M1 macrophages can disrupt osteoblast formation by secreting tumor necrosis factor-α (TNF-α) (9). Certain subsets of pro-inflammatory T cells may promote osteoblast differentiation during the early stages of fracture healing, but it is currently unclear whether T cells will exert the same effects in the persistent inflammatory environment of osteoporosis (10). B cells play a role in regulating the receptor activator of nuclear factor kappa-B ligand/osteoprotegerin (RANKL/OPG) axis, which affects osteoclast differentiation. They can inhibit osteoclast differentiation by secreting OPG (1). Breg cells, a subset of B cells, can inhibit the differentiation of Th17 cells by secreting interleukin-10 (IL-10) and transforming growth factor-beta (TGF-β). This inhibition leads to a decrease in the secretion of inflammatory factors by monocytes and dendritic cells (11). so, promoting B cell differentiation can have immunosuppressive effects and may also promote the maturation and growth of osteogenic precursor cells. It is hypothesized that there is a causal relationship between immune cells and osteoporosis. However, due to limitations in sample size, study design flaws, and confounding factors, it is currently not possible to clearly elucidate this relationship (12–15). Therefore, further research is necessary to clarify the causal relationship between immune cells, including B cells, and osteoporosis.

The method of Mendelian randomization (MR) combines instrumental variables (IVs) and Mendel’s laws of inheritance (16). It overcomes limitations of traditional randomized controlled trials by minimizing the impact of confounding factors (17). Compared to observational analyses, MR offers several advantages: (1) it maintains the proper sequence of exposure and outcome, preventing reverse causality; (2) it reduces the influence of confounding factors on the results; (3) it enables long-term studies of exposure and outcome (IVs remain valid throughout a lifetime); (4) it avoids regression dilution resulting from testing errors, thanks to the accurate genotype testing (18, 19). In this study, a comprehensive two-sample bidirectional MR analysis was conducted to establish the causal relationship between immune cell characteristics and osteoporosis.

## 2 Materials and methods

### 2.1 Study design

We employed a two-sample MR analysis to investigate the causality between 731 immune cell characteristics and osteoporosis. The IVs used in the analysis satisfied the following critical assumptions: genetic variation exhibits a direct association with exposure factors; genetic variation remains independent of possible confounders; and genetic variation does not affect outcome through pathways other than exposure.

### 2.2 Genome-wide association study data sources

The GWAS summary statistics for osteoporosis were obtained from the IEU openGWAS public database (Osteoporosis GWAS id: ebi-a-GCST90038656). The study included GWAS data from 484,598 individuals, with 7,751 cases and 476,847 controls. For each immune trait, GWAS summary statistics are available in the GWAS Catalog with accession numbers ranging from GCST0001391 to GCST0002121 (20, 21). The dataset includes a total of 731 immunophenotypes, which consist of various features such as absolute cell (AC) counts (118 traits), median fluorescence intensities (MFI) reflecting surface antigen levels (389 traits), morphological parameters (MP) (32 traits), and relative cell (RC) counts (192 traits). The MFI, AC, and RC features encompass various immune cell types, including B cells, CDCs, mature stages of T cells, monocytes, myeloid cells, TBNK (T cells, B cells, natural killer cells), and Treg panels. The MP feature includes CDC and TBNK panels. These immunophenotypes provide valuable information for investigating the relationship between immune cell characteristics and osteoporosis through two-sample MR analysis.

### 2.3 Screening mendelian randomized IVs

In the first step of the analysis, the IVs was set at 1×10^-5^, based on previous related studies (20, 22). The second step involved removing single nucleotide polymorphisms (SNPs) in linkage disequilibrium (LD) with each other, with a parameter setting of r2 = 0.001 and kb=10,000. This step helps to avoid bias caused by the correlation between SNPs in close proximity to one another. Finally, if any of the selected SNPs were found to be associated with confounding factors that significantly correlated with the outcome (p value <5×10^−8^), they were removed from the list of selected SNPs. This step is important to ensure that the observed associations are not due to the influence of other factors that may affect both the genetic variant and the outcome of interest (23). The R package used in this study is available from a public database (URL: https://github.com/ZDQZBXZ/731-Immune-Cell-Code).

### 2.4 Statistical analysis

The analysis process was conducted using R 3.5.3 software (http://www.Rproject.org). A Mendelian Randomization (MR) analysis employing a two-sample approach was carried out on GWAS data to investigate the causal link between 731 immune cell types (as exposures) and osteoporosis (as the outcome), with the Wald ratio (WR) combined inverse variance weighting (IVW) strategy as the principal analytic method (24). Where MR results showed heterogeneity, the random effects version of IVW was applied; in its absence, a fixed effects model was adopted. To enhance the robustness and dependability of the findings, additional methods such as MR-Egger regression, Weighted Median Regression (WMR), and MR Pleiotropy RESidual Sum and Outlier (MR-PRESSO) were used alongside IVW (25). Specifically, the IVW method aims to assess the overall population by combining the effect estimates of each individual SNP in MR studies. It’s important to note that if a single SNP contributes more than 50% of the weight in all relevant SNP pairs, or if multiple SNPs collectively exceed half of the weight, it can lead to horizontal pleiotropy. Therefore, in this study, the WMR method was employed as an alternative strategy to address situations where IVW findings might lack precision (26). Indeed, the IVW method assumes its intercept must pass through zero (27). However, this assumption may not accommodate scenarios where the intercept deviates from zero. To address this limitation, the MR-Egger method was employed as an additional strategy (28). The use of the MR-PRESSO method aimed to detect and correct biases arising from pleiotropy (29). In this study, data adjusted for pleiotropy via MR-PRESSO were incorporated to ensure potential outliers were appropriately addressed. F-statistics were calculated as measures of IV strength, with values greater than 10 indicating minimal susceptibility to weak instrument bias (30). IVs failing to meet the criterion of F ≥ 10 were excluded, ensuring robust IV strength. Sensitivity checks including the Cochran’s Q test, MR-Egger intercept test, and MR-PRESSO global test were performed, ensuring the solidity of the results, evidenced by all MR-Egger intercept tests having p-values over 0.05, thus excluding the presence of a horizontal pleiotropy effect (31). In the event of inconsistency in MR findings, a reanalysis with stricter cutoffs for the p-value of instrumental variables and more rigorous selection criteria was executed. Scatter plots offered a graphical display of the reevaluated data, while funnel plots were employed to scrutinize any potential publication bias.

## 3 Results

**Figure 1:**
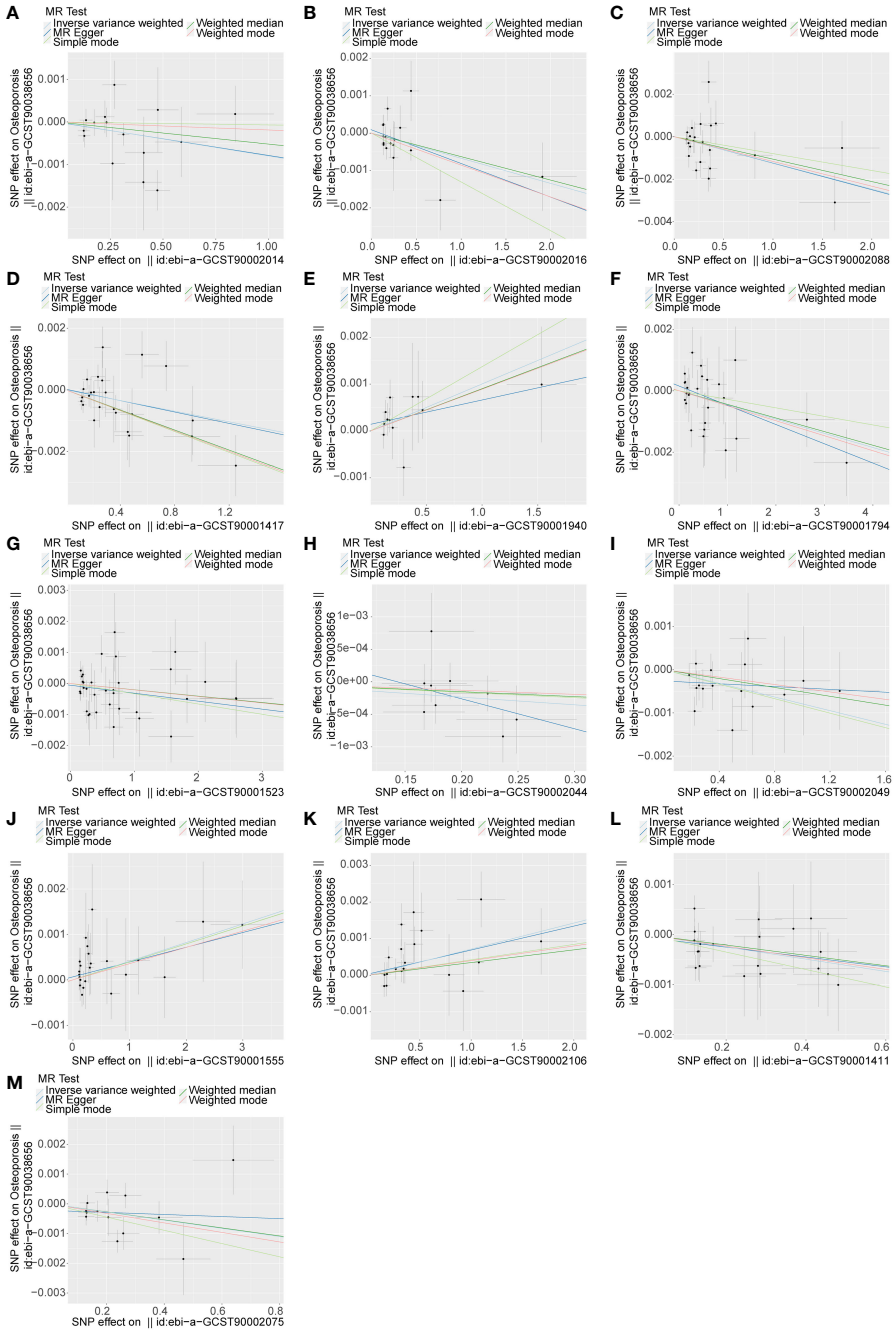
The causal relationship between 13 immune cell phenotypes and osteoporosis. An upward slope indicates exposure as a risk factor and a downward slope indicates exposure as a protective factor. The outcome is osteoporosis. **(A)** exposure factor is CCR2 on CD62L+ myeloid DC. **(B)** exposure factor is CCR2 on CD62L+ plasmacytoid DC. **(C)** exposure factor is CD11c on CD62L+ myeloid DC. **(D)** exposure factor is CD24+ CD27+ %B cell. **(E)** exposure factor is CD25 on CD39+ activated Treg. **(F)** exposure factor is CD25 on IgD+. **(G)** exposure factor is CD33- HLA DR+ AC. **(H)** exposure factor is CD45 on CD33dim HLA DR+ CD11b-. **(I)** exposure factor is CD45 on Mo MDSC. **(J)** exposure factor is EM CD8br %CD8br. **(K)** exposure factor is HLA DR on DC. **(L)** exposure factor is IgD+ CD24+ %B cell. **(M)** exposure factor is SSC-A on B cell.

**Figure 2:**
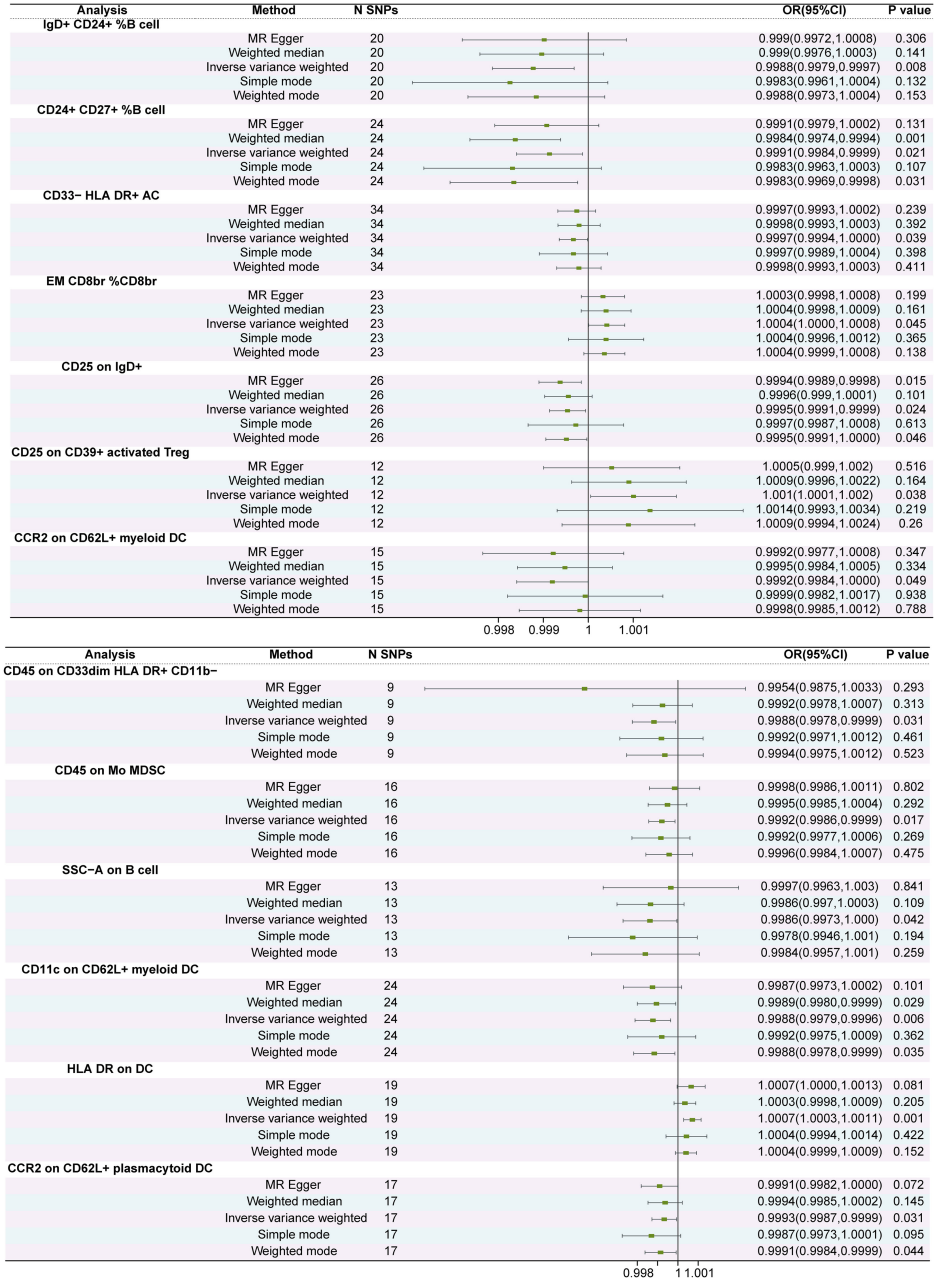
The forest plot of the causal relationship between 13 immune cell phenotypes and osteoporosis. The inverse variance weighting method is used as the primary outcome p < 0.05 is considered causal relationship.

In this study, we used a two-sample bidirectional MR method to explore the relationship between 731 immune cell phenotypes and osteoporosis. Regarding the causal analysis of immune cells for osteoporosis, we observed a total of 13 different immune cell phenotypes that have an effect on osteoporosis. Of these 3 immune cell phenotypes were osteoporosis risk factors and 10 immune cell phenotypes provided osteoporosis protection (**Figure 1**; **Supplementary File 1**). The results of IVW analysis were referenced as the primary assessment tool for the study. The IVW results for 13 immune cell phenotypes are shown in **Table 1**. Specifically, the odds ratio (OR) of *IgD+ CD24+ %B cell* (B cell panel) risk on Osteoporosis was estimated to be 0.9986 (95% CI = 0.9978~0.9996, *P*<0.01). The OR of *CD24+ CD27+ %B cell* (B cell panel) risk on Osteoporosis was estimated to be 0.9991 (95% CI = 0.9984~0.9998, *P* = 0.021). The OR of *CD33- HLA DR+AC* (Myeloid cell panel) risk on Osteoporosis was estimated to be 0.9996 (95% CI = 0.9993~0.9999, *P* = 0.038). The OR of *EM CD8br %CD8br* (Maturation stages of T cell panel) risk on Osteoporosis was estimated to be 1.0004 (95% CI = 1.0000~1.0008, *P* = 0.045). The OR of *CD25 on IgD+* (B cell panel) risk on Osteoporosis was estimated to be 0.9995 (95% CI = 0.9991~0.9999, *P* = 0.024). The OR of *CD25 on CD39+ activated Treg+* (Treg panel) risk on Osteoporosis was estimated to be 1.001 (95% CI = 1.0001~1.0019, *P* = 0.038). The OR of *CCR2 on CD62L+ myeloid DC* (cDC panel) risk on Osteoporosis was estimated to be 0.9992 (95% CI = 0.9984~0.9999, *P* = 0.048). The OR of *CCR2 on CD62L+ plasmacytoid DC* (cDC panel) risk on Osteoporosis was estimated to be 0.9993 (95% CI = 0.9987~0.9999, *P* = 0.035). The OR of *CD45 on CD33dim HLA DR+ CD11b-* (Myeloid cell panel) risk on Osteoporosis was estimated to be 0.9988 (95% CI = 0.9977~0.9998, *P* = 0.031). The OR of *CD45 on Mo MDSC* (Myeloid cell panel) risk on Osteoporosis was estimated to be 0.9992 (95% CI = 0.9985~0.9998, *P* = 0.017). The OR of *SSC-A on B cell* (TBNK panel) risk on Osteoporosis was estimated to be 0.9986 (95% CI = 0.9972~0.9999, *P* = 0.042). The OR of *CD11c on CD62L+ myeloid DC* (cDC panel) risk on Osteoporosis was estimated to be 0.9987 (95% CI = 0.9978~0.9996, *P*<0.01). The OR of *HLA DR on DC* (cDC panel) risk on Osteoporosis was estimated to be 1.0007 (95% CI = 1.0002~1.0011, *P*<0.01) (**Figure 2**; **Supplementary File 2**). Sensitivity analysis of 13 immune cells to osteoporosis was uploaded as a supplementary file (**Supplementary File 3**). There was no gene pleiotropy for 13 immune cell phenotypes, indicating that the results are reliable (**Supplementary File 4**). Publication bias and funnel plot results are also presented as supplementary documents (**Supplementary File 5**). No causal effect of osteoporosis on immune cells was observed (**Supplementary File 6**). The raw data of 731 immune cell phenotypes after excluding weak IVs, along with F-statistics for 13 immune cell phenotypes, are detailed in **Supplementary File 7**.

**Table 1:** The IVW results for 13 immune cell traits.

immune cell traits	beta coefficients	standard error	OR values	95% confidence	p-value	Trait type
IgD+ CD24+ %B cell	-0.00123	0.000464153	0.9986	0.9978~0.9996	<0.01	Relative count
CD24+ CD27+ %B cell	-0.00086	0.000374428	0.9991	0.9984~0.9998	0.021	Relative count
CD33- HLA DR+ AC	-0.00033	0.000160646	0.9996	0.9993~0.9999	0.038	Absolute count
EM CD8br %CD8br	0.00041	0.000204786	1.0004	1.0000~1.0008	0.045	Relative count
CD25 on IgD+	-0.00046	0.000204577	0.9995	0.9991~0.9999	0.024	MFI
CD25 on CD39+ activated Treg	0.00100	0.000485582	1.0010	1.0001~1.0019	0.038	MFI
CCR2 on CD62L+ myeloid DC	-0.00080	0.000405533	0.9992	0.9984~0.9999	0.048	MFI
CCR2 on CD62L+ plasmacytoid DC	-0.00067	0.000310695	0.9993	0.9987~0.9999	0.035	MFI
CD45 on CD33dim HLA DR+ CD11b-	-0.00118	0.000547639	0.9988	0.9977~0.9998	0.031	MFI
CD45 on Mo MDSC	-0.00079	0.000331975	0.9992	0.9985~0.9998	0.017	MFI
SSC-A on B cell	-0.00138	0.000677511	0.9986	0.9972~0.9999	0.042	Morphological parameter
CD11c on CD62L+ myeloid DC	-0.00123	0.000449283	0.9987	0.9978~0.9996	<0.01	MFI
HLA DR on DC	0.00071	0.000219126	1.0007	1.0002~1.0011	<0.01	MFI

**Table 1** displays the IVW results for 13 immune cell traits causally linked to osteoporosis. The 95% confidence intervals represent the 95% confidence intervals for OR values. The ‘type’ of various immune cells can be referenced in the Trait type column. The original data for IVW are retained in **Supplementary File 1**.

## 4 Discussion

Utilizing the genetic information from publicly accessible databases, our investigation delved into the potential causative links between 731 immune cell characteristics and osteoporosis via MR. As far as we are aware, Mendelian studies are the current focus of much scientific attention; however, the investigation into the connection between immune cells and diseases remains largely unexplored. Our MR analysis is pioneering in examining the causative links between a variety of immune phenotypes and osteoporosis, thereby laying some groundwork for the concept of immune-related osteoporosis. Our research identified 13 immune cell phenotypes spanning four categories of immune traits (MFI, RC, AC, and MP) that have a causal relationship with osteoporosis. Within these traits, 3 phenotypes were identified as risk factors, while 10 were established as protective factors, all with a *P* value of less than 0.05.

According to MR results, the traits IgD+ CD24+ %B cells, CD24+ CD27+ %B cells, and CD25 on IgD+ (B cell panel) have been identified as protective factors against osteoporosis. These three phenotypes all reflect the expression of surface-active substances on B cells. Typically, common markers found on the surface of B cells are utilized to isolate and characterize the various subgroups within the B cell population. To elaborate, IgD is a membrane-bound immunoglobulin on B cells that binds to antigens and participates in the B cell immune response (32, 33). CD24 is a cell surface marker involved in cell-to-cell interactions and interactions between cells and their external microenvironment, and it is associated with the adhesion and migration capabilities of B cells (33–35). The term IgD+ CD24+ %B cells indicates the percentage of B cell subgroups that co-express IgD and CD24 within the overall B cell population (the same principle applies to the other phenotypic characteristics mentioned in the text). Consequently, MR analyses suggest that a higher proportion of B cell subgroups expressing IgD and CD24 correlates with a reduced risk of osteoporosis. IgD plays a crucial role within the adaptive immune system, participating in the B-cell driven antibody-mediated immune reactions (36). Studies indicate that higher IgD levels and lower IgM levels on B cells correspond with reduced B cell activity in human peripheral blood (36–40). However, the connection between these observations and osteoporosis remains unclear. It’s hypothesized that the suppressive activity of B cells may interact with the development and expansion of bone-forming progenitor cells (11). Whether the control of B cell functionality through IgD surface expression is implicated in bone metabolism requires comprehensive investigation. CD24 participates in the osteogenic differentiation process of bone marrow mesenchymal stem cells (BMSCs) and acts as a signaling molecule within this context (41, 42). Study has demonstrated that CD24, located on the cell surface, displays a notable disparity in expression levels when BMSCs undergo differentiation into osteogenic or lipogenic lineages, with reduced CD24 expression being associated with diminished osteogenic differentiation (43). CD24 is currently proposed as a selective biomarker for a subpopulation of BMSCs with enhanced osteogenic potential (33). CD27, on the other hand, is a molecular marker on the cell membrane commonly used to differentiate various subgroups of memory B cells (44, 45). A previous MR study has reported a causal relationship between CD27 and bone density (46). CD25, also known as the IL-2 receptor alpha chain, is another cell surface marker that characterizes a cell surface receptor and plays a crucial role in regulating the activation, proliferation, and differentiation of T and B cells (47, 48). Evidence on CD25 expression on the surface of B cells and osteoporosis is limited, so this finding is revealing.

The term “SSC-A on B cell (TBNK panel)” refers to the quantification and assessment of the side scatter signal intensity (SSC-A) observed in B cells within the TBNK panel for cell analysis. SSC-A indicates the intensity of side scatter signals detected during flow cytometry, providing insights into cellular dimensions, shape, and intracellular complexity (21). Generally, a heightened SSC-A reading may indicate a greater cell population, though it does not directly quantify the actual number of cells. So, MR results from this research demonstrate a negative causal link between side scatter signal intensity on B cells (SSC-A) and osteoporosis. Moreover, the immunosuppressive characteristics of B cells, along with the presentation of certain proteins on their surface, play a significant role in providing defense against osteoporosis. However, further discussion is needed on the two main aspects of the potential relationship between B cells and osteoporosis: an osteoprotective function in physiological conditions and a bone-destructive function in pathological states. In the bone marrow microenvironment, B lymphocytes are unable to mature in the absence of secretory factors from mesenchymal and osteoblastic cells (49). Bruton’s tyrosine kinase (BTK) is an enzyme integral to signaling in pre-B cell receptors (pre-BCRs) (50). As part of their maturation process, B cells participate in the assembly of pre-BCRs via V(D)J recombination (49). Upon successful pre-BCR formation, BTK engages in subsequent signaling pathways (51). Interestingly, BTK signaling unexpectedly plays a crucial role in the differentiation of osteoclasts. When BTK signaling is impaired, osteoclasts become dysfunctional and bone resorption is diminished (52). OPG, belonging to the tumor necrosis factor (TNF) receptor superfamily, competitively interacts with RANKL, inhibiting the interaction of RANKL with its receptor RANK—a critical step in osteoclastogenesis and activation (53). There is previous evidence that B cells under physiological conditions can secrete OPG (54), and in the bone marrow, B cells contribute nearly half of the OPG (55), suggesting that B lymphocytes are crucial in maintaining bone integrity by mitigating excessive bone resorption. This means that B lymphocytes play a very important role in regulating bone health, particularly in preventing excessive bone resorption.

In instances of osteoporosis pathology, notably under conditions of chronic inflammation commonly associated with postmenopausal osteoporosis in women, B lymphocytes can contribute to bone destruction (56). Unlike senile osteoporosis, which is characterized by reduced bone resorption and even more significant decreases in bone formation (57), postmenopausal osteoporosis sees a marked increase in osteoclast numbers and a surge in bone resorption (58). More critically, the abrupt decline in estrogen triggers an inflammatory state and elevates inflammatory factors within the bone milieu, which in turn stimulates B lymphocytes to produce more granulocyte macrophage colony-stimulating factor (GM-CSF) (59). This factor has been shown to promote proliferation in osteoclast progenitor cells and an uptick in osteoclast numbers (60). Additionally, under inflammatory conditions, activated B cells secrete RANKL, which is instrumental in the activation of osteoclast formation (61). Besides osteoporosis in postmenopausal women, smoking and chronic diseases such as obesity, diabetes, and hypertension can also lead the body to a state of chronic inflammation. Compared to the physiological state where OPG secretion inhibits osteoclasts, B cells in pathological states promote osteoclasts. However, it is undeniable that B cells appear to play a significant role in the regulation of osteoclastogenesis through the RANKL/OPG signaling pathway. This study has identified only three B lymphocyte subpopulations and the SSC-A on B cell marker that are protective against osteoporosis. Due to MR analysis avoiding confounding bias and reverse causation, the results have some significance, but whether these cell subpopulations have potential roles in conjunction with the RANKL/OPG signaling system remains to be further investigated.

The term “EM CD8br %CD8br (Maturation stages of T cell panel)” denotes a specific subset of T cells within the T cell differentiation process that are characterized by an effective memory function, high CD8 expression, and comprise a certain percentage of the overall T cell population. This subset of T cells, known as memory CD8+ T cells, is found persistently in the peripheral blood of patients who suffer from delayed bone healing. Memory CD8+ T cells have been observed to hinder the activity and osteogenic differentiation of bone marrow stromal cells by excessively producing interferon-gamma (IFN-γ) (62). This was also suggested as an osteoporosis risk factor in the MR results. CD39 is prevalently located on the surface of Treg cells and is part of the Nucleoside Triphosphate Diphosphohydrolase (NTPDase) superfamily, and it plays a significant role in T cell functionality, storage, and proliferation (63–65). The term “activated Treg+” refers to a subset of Treg cells that express both CD39 and CD25 and are in an activated state, exhibiting regulatory properties in response to antigen stimulation. Evidence found that CD39 mediates osteoporosis by regulating the balance between osteoclasts and osteoblasts through the Wnt/β-collagen pathway (66). Additional evidence corroborates that soluble pro-inflammatory cytokines IL-17A and IL-17F, secreted by T cells (Th17 cells), are vital mediators for the maturation and differentiation of osteoblasts, and play a crucial role in enhancing bone formation as well as facilitating the healing of fractures (67, 68). Despite the known association of various cytokines, proteins, and T cells with osteoporosis, the intricate processes related to bone metabolism and remodeling involving CD25 and CD39, which are expressed on the T cells, remain a mystery (69).

In our study, CD11c on CD62L+ myeloid DC, CCR2 on CD62L+ myeloid DC, and CCR2 on CD62L+ plasmacytoid DC were all observed to be protective factors against osteoporosis. CD62L, also known as L-selectin, is an adhesion molecule found primarily on the surface of lymphocytes and indicated by certain dendritic cells (70, 71). Down-regulation of CD62L antigen has been suggested as a possible mechanism for neutrophilia during Immune response (72, 73). A previous study reported decreased CD62L expression in the spleen and increased expression in the bone marrow during inflammation-induced osteoporosis (74). CCR2 serves as a chemokine receptor on the cell membrane, playing a critical role in the migration of monocytes and the management of inflammation-associated activities (75). It has been associated with a decline in bone mass and the facilitation of bone degradation (76). CCR2 can also be found on preosteoblasts and osteoblasts, where its interaction with the chemokine ligand 2 (CCL2) is thought to contribute to the fusion and development of osteoblasts (77–79). Research indicates that the removal or blocking of CCR2 results in a reduction in both the quantity and the functionality of fully formed osteoclasts, as well as mitigated bone resorption (80). According to MR findings from the current study, CCR2 activity on both plasmacytoid and conventional dendritic cells acts as a defensive mechanism against osteoporosis, aligning with the prevailing body of evidence.

MR results show that CD45 on CD33dim HLA DR+ CD11b-, CD45 on Mo MDSC, and CD33- HLA DR+ AC were protective factors against osteoporosis, and all three were associated with Myeloid cells. Where CD33dim indicates a lower level of CD33 expression and CD11b- indicates no expression of CD11b.Mo-MDSC stands for monocyte-derived myeloid-derived suppressor cells, which play an important role in the regulation of immune responses, inflammation, and the tumor microenvironment (81). CD45 is a common leukocyte co-antigen that plays an important role in immune responses and cell signaling (82). For T cells, the extracellular structural domain of CD45 is expressed in several different isoforms, and the expression of specific isoforms depends on specific cellular subpopulations. The nature of the ligands for the different isoforms of CD45 is still uncertain, and the exact mechanism by which the potential ligands regulate CD45 function is not known (83). CD45 is inextricably linked to the immune system, and current studies have elucidated significant associations between CD45 in systemic lupus erythematosus, rheumatoid arthritis, and HIV (84). Dysfunction of CD45 has also been associated with hematologic malignancies and Alzheimer’s disease (85). Some evidence suggests that perturbations in CD45 activity may lead to the development of autoimmune diseases (86, 87). Despite the abundant expression of CD45, its role in myeloid cells is unclear, and there is no definitive proof indicating an association with osteoporosis (88). HLA-DR is an MHC class II cell surface receptor encoded by the human leukocyte antigen complex on chromosome 6, region 6P21. A statistically significant association was found between the number of HLA-DR mismatches and the diagnosis of osteoporosis in renal transplant patients (89). Increased immune response due to HLA-DR mismatch may be associated with osteoporosis and hip fracture development (90). CD33 is a myeloid-specific antigen that is highly expressed in myeloid cells. CD33 plays an important role in immune regulation and cell adhesion functions (91). CD11b is an adhesion molecule expressed in monocytes and myeloid cells, which facilitates cell adhesion and migration (92, 93). The low expression of CD11b and CD33 in myeloid cells may limit their cell adhesion and migration functions, suggesting a negative causal effect on osteoporosis, which is enlightening.

Before this study, research (Cao’s study) had already reported causal relationships between immune cell phenotypes and bone mineral density (BMD). Therefore, it is important to discuss the necessity of this study. Cao’s study reported a total of 53 causal relationships between different immune cell phenotypes and BMD (see Cao’s study **Supplementary Table 2**) (46). Similar to this study, neither found causal effects of osteoporosis or BMD on immune cells. CD45 on CD33dim HLA DR+ CD11b- was the same exposure factor found in both studies, one study defined its outcome as total body BMD, while this study defined its outcome as osteoporosis. Using BMD as an outcome may lean more towards assessing the overall impact of immune cells on bone density. Using osteoporosis as an outcome aims to better understand the specific role of immune cells in the development of osteoporosis. Therefore, the causal relationship between the other 12 immune cell phenotypes and osteoporosis, which was found in this study, is unreported and holds clinical significance. In this study, we identified and extensively discussed the complex causal role of B cells in osteoporosis, whereas Cao’s study focused on the correlation between the CD40/CD40L system and bone metabolism. In addition, based on existing literature evidence, this study discusses in detail the potential links between each immune phenotype traits and osteoporosis The results of these two studies complement each other, providing insights from different perspectives into the potential causal relationships between osteoporosis and immune cells.

Through our study, we found that four immune cell characteristics, including absolute cell counts, median fluorescence intensities reflecting surface antigen levels, morphological parameters, and relative cells all had different causal relationships with osteoporosis. CD24, CD27, CD25 from B cells are protective factors against osteoporosis, and similar findings have been discussed, but more in-depth mechanisms of action remain to be investigated. The signal intensity of side scatter (SSC-A) on B cells reflects the protective effect of osteoporosis, which is related to the comprehensive assessment of B cell size, number, complexity, and granular content. Therefore, several active factors on B cells and a certain degree of B cells may be beneficial for osteoporosis. It must be mentioned that there are two risk factors associated with T cells. The EM CD8br %CD8br (Maturation stages of T cell panel) primarily represents effector T cells that have undergone stimulation and differentiation in immune response and possess the ability for rapid response. These cells are characterized by high expression of CD8. Another factor is a subset of regulatory T cells (Tregs), which are characterized by the surface expression of CD25 and CD39. Additionally, these Treg cells display an activated state. However, the regulation of T cells in osteoporosis is complex and diverse, and further evidence is still worth exploring.

### 4.1 Limitations

Due to the self-reported nature of the diagnostic data for osteoporosis in the GWAS database with the identifier ebi-a-GCST90038656, there is a potential for a certain degree of bias in the results. Due to database limitations, we were unable to stratify osteoporosis by gender, age, race, and whether or not they smoked, and we will work to address this issue in our upcoming studies.

## 5 Conclusions

In conclusion, we demonstrated a causal relationship between 13 immune phenotypes and osteoporosis through comprehensive bidirectional MR analysis, highlighting the complex pattern of interactions between the immune system and osteoporosis. In addition, our study significantly reduced the effects of unavoidable confounders, reverse causality, and other factors. This may provide new avenues for researchers to explore the biological mechanisms of osteoporosis and help to explore early intervention and therapeutic approaches.

## Data availability statement

The original contributions presented in the study are included in the article/**Supplementary Material**. Further inquiries can be directed to the corresponding author.

## Author contributions

DZ: Data curation, Formal analysis, Methodology, Software, Writing – original draft, Writing – review & editing. CZ: Writing – review & editing. GG: Writing – review & editing. DZ: Writing – review & editing. QC: Data curation, Methodology, Software, Supervision, Writing – original draft, Writing – review & editing.

## Supplementary material

Supplementary File 1 Results of the causal effect of immune cells on osteoporosis.

Supplementary File 2 Sensitivity analysis results of immune cells on osteoporosis.

Supplementary File 3 Scatterplots of immune cells on osteoporosis.

Supplementary File 4 Pleiotropy and Heterogeneity of immune cells on osteoporosis.

Supplementary File 5 Supplementary figures.

Supplementary File 6 Causal effects of osteoporosis on immune cells.

Supplementary File 7 Removal of weak IVs and F-statistics.
