# Supplementary material for: An exploration of the causal relationship between 731 immunophenotypes and osteoporosis: a bidirectional Mendelian randomized study
Source: Front Endocrinol (Lausanne). 2024 Jul 17;15:1341002. doi: 10.3389/fendo.2024.1341002 (PMC11288873; doi:10.3389/fendo.2024.1341002)
Supplement: Supplementary File 1 — Results of the causal effect of immune cells on osteoporosis. [file DataSheet_1.zip › Supplementary file 1/CD25 on IgD+.pdf]

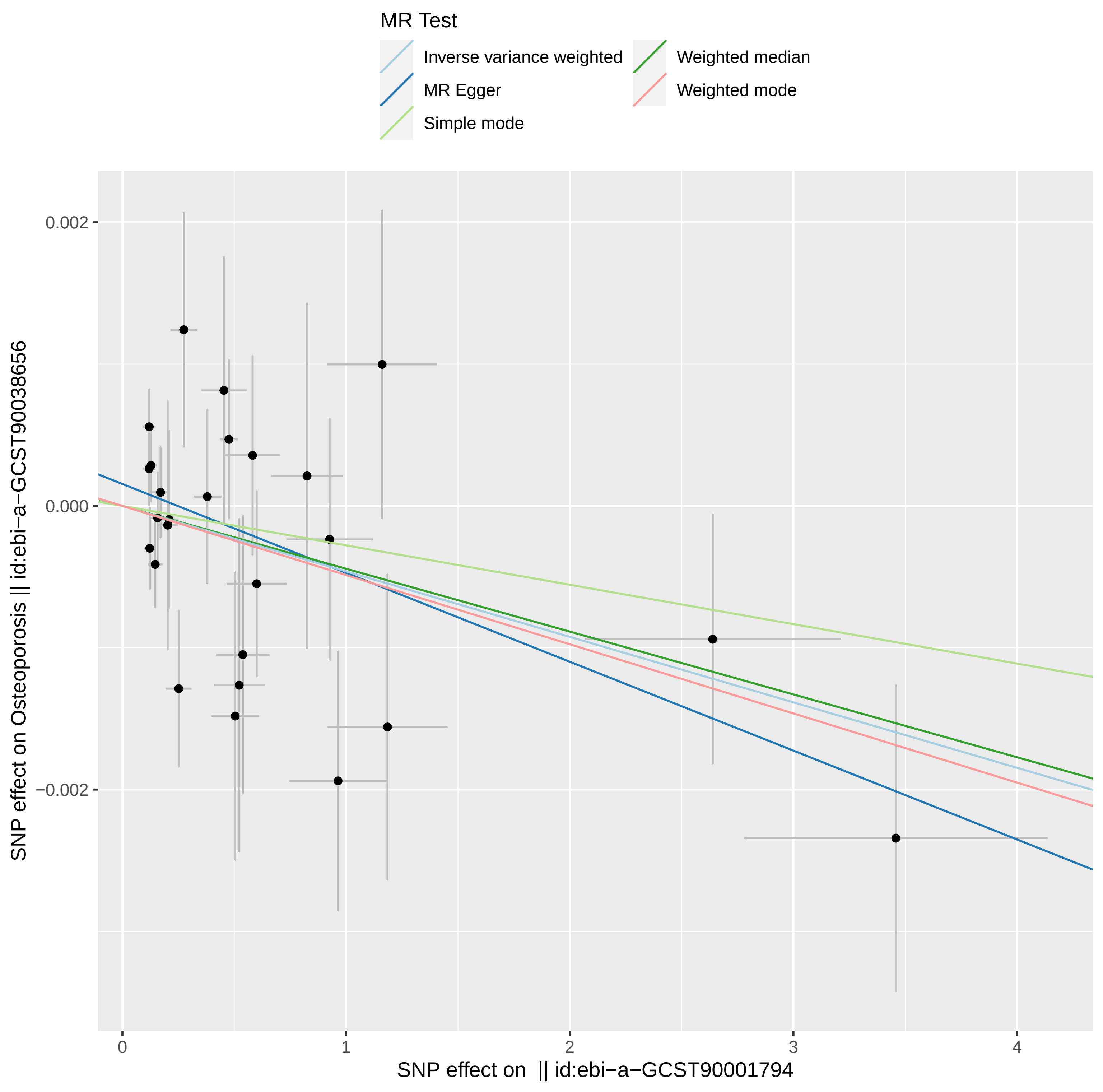
MR Test
Inverse variance weighted
MR Egger
Simple mode
Weighted median
Weighted mode
0.002
0.000
−0.002
0
1
2
3
4
SNP effect on Osteoporosis || id:ebi−a−GCST90038656
SNP effect on || id:ebi−a−GCST90001794
