# Supplementary figures and images for: An exploration of the causal relationship between 731 immunophenotypes and osteoporosis: a bidirectional Mendelian randomized study
Source: Front Endocrinol (Lausanne). 2024 Jul 17;15:1341002. doi: 10.3389/fendo.2024.1341002 (PMC11288873; doi:10.3389/fendo.2024.1341002)

# MR Test

- Inverse variance weighted
- MR Egger
- Simple mode
- Weighted median
- Weighted mode

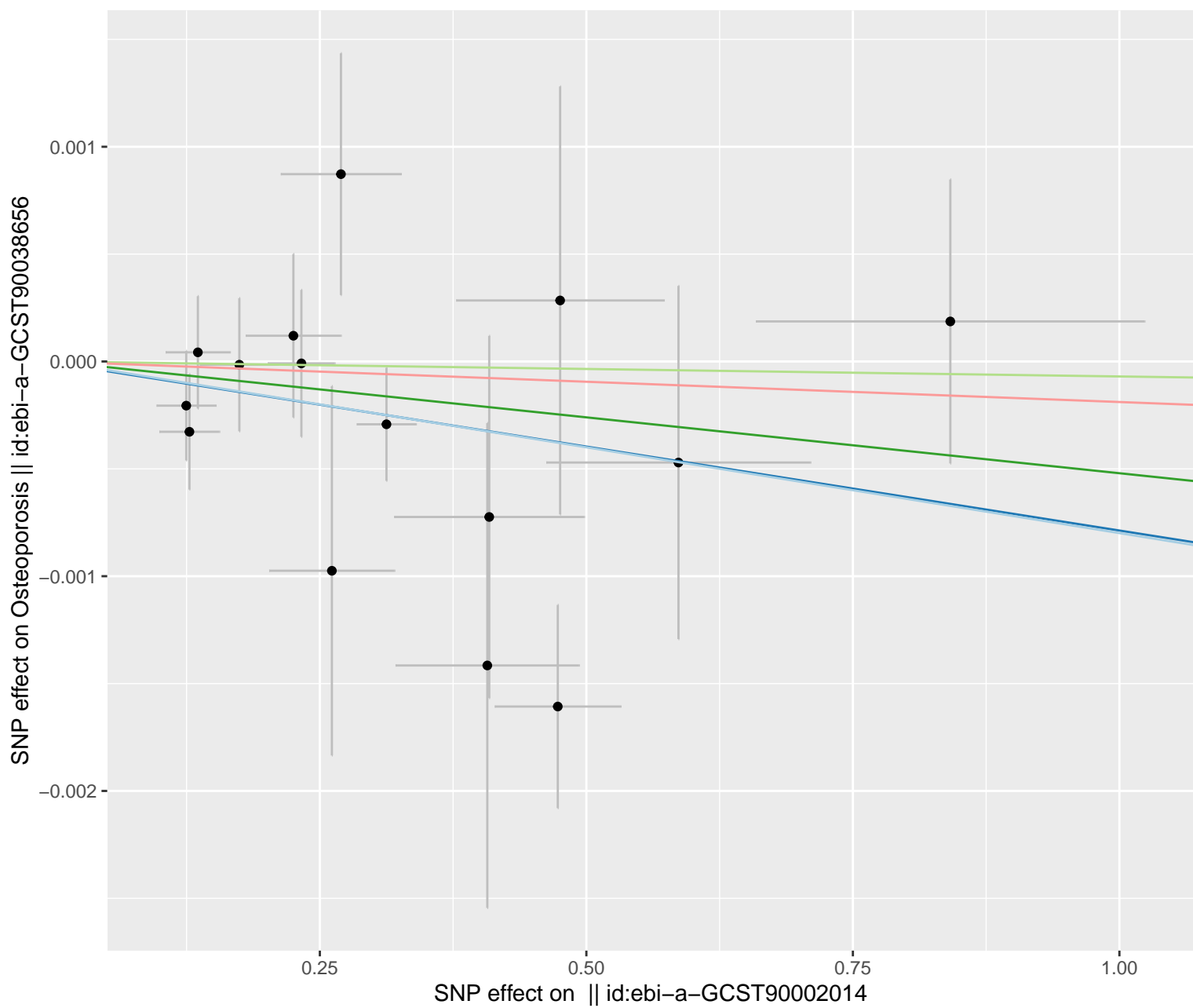

Supplement: Supplementary File 1 — Results of the causal effect of immune cells on osteoporosis. [file DataSheet_1.zip › Supplementary file 1/CCR2 on CD62L+ myeloid DC.pdf]

# MR Test

- Inverse variance weighted
- MR Egger
- Simple mode
- Weighted median
- Weighted mode

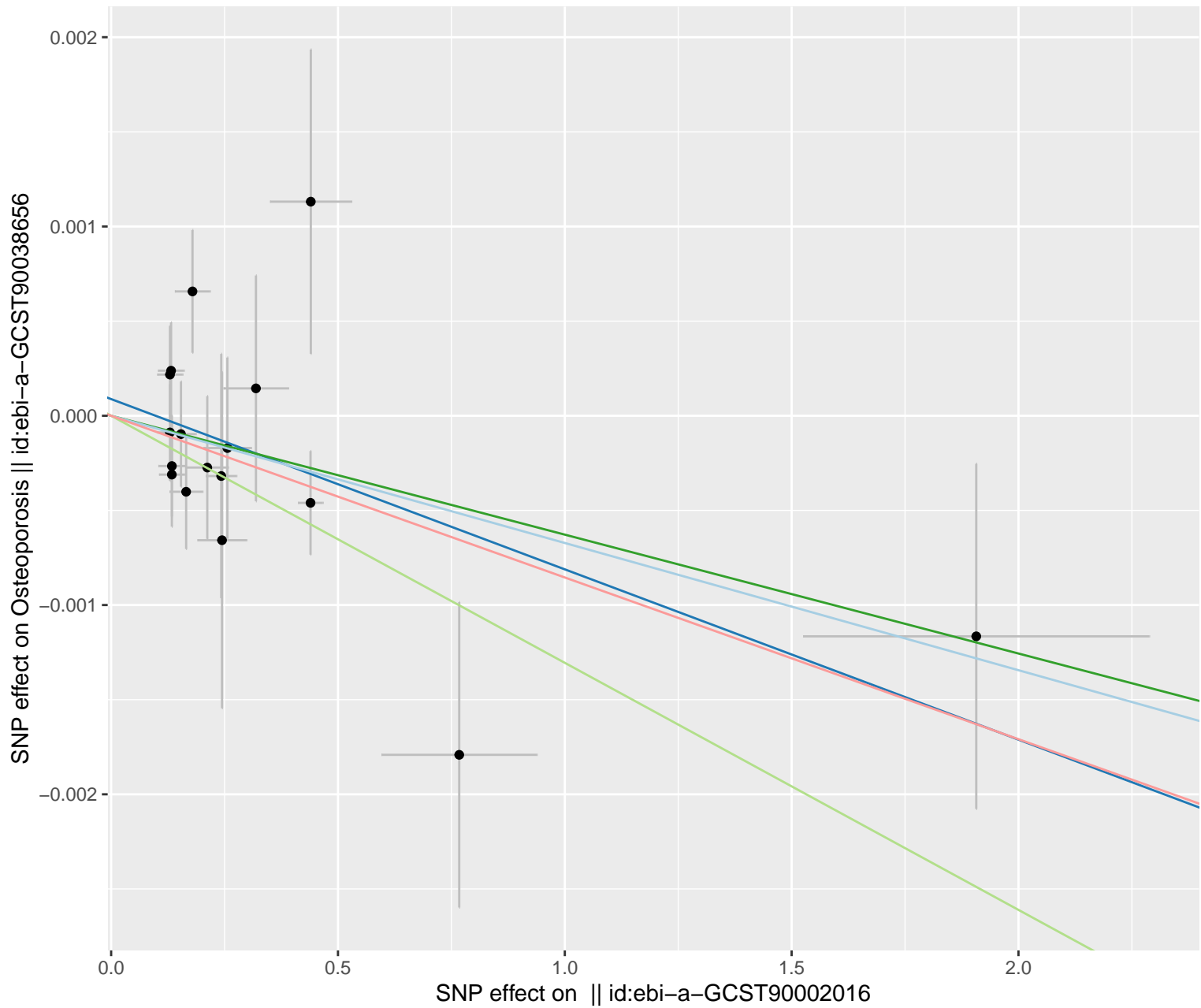

Supplement: Supplementary File 1 — Results of the causal effect of immune cells on osteoporosis. [file DataSheet_1.zip › Supplementary file 1/CCR2 on CD62L+ plasmacytoid DC.pdf]

# MR Test

- Inverse variance weighted
- MR Egger
- Simple mode
- Weighted median
- Weighted mode

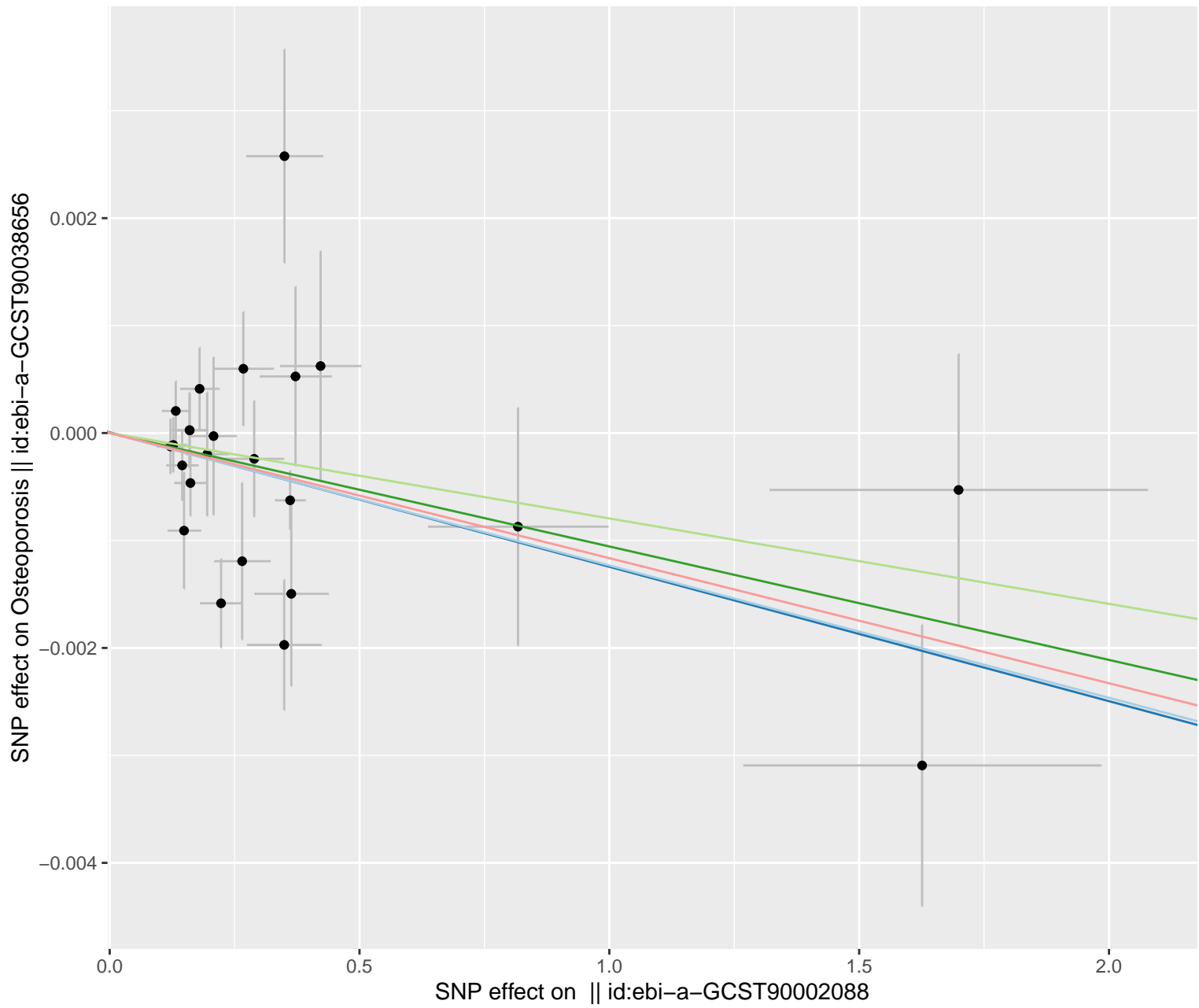

Supplement: Supplementary File 1 — Results of the causal effect of immune cells on osteoporosis. [file DataSheet_1.zip › Supplementary file 1/CD11c on CD62L+ myeloid DC.pdf]

# MR Test

- Inverse variance weighted
- MR Egger
- Simple mode
- Weighted median
- Weighted mode

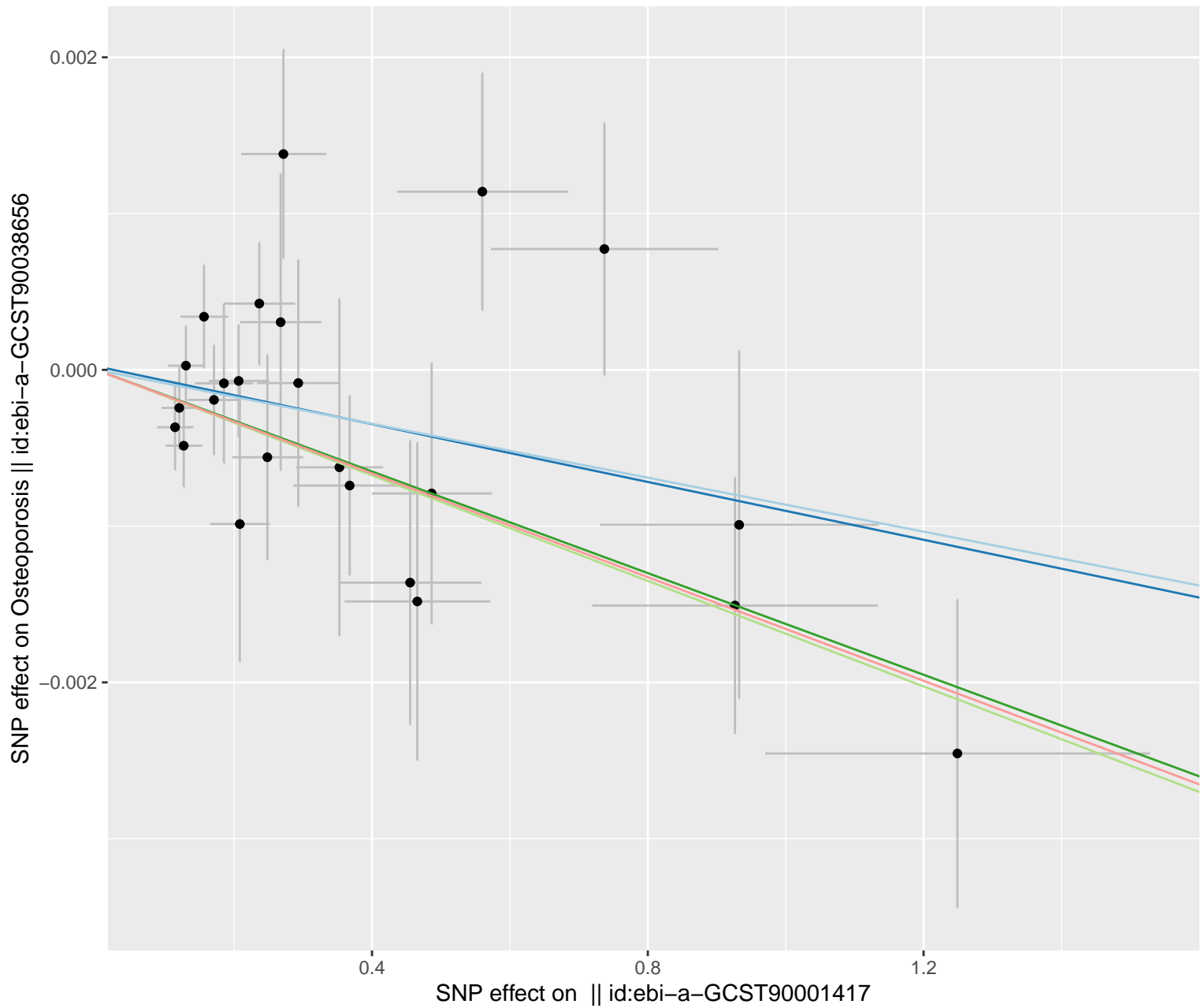

Supplement: Supplementary File 1 — Results of the causal effect of immune cells on osteoporosis. [file DataSheet_1.zip › Supplementary file 1/CD24+ CD27+ %B cell.pdf]

## MR Test

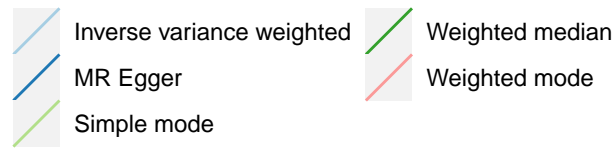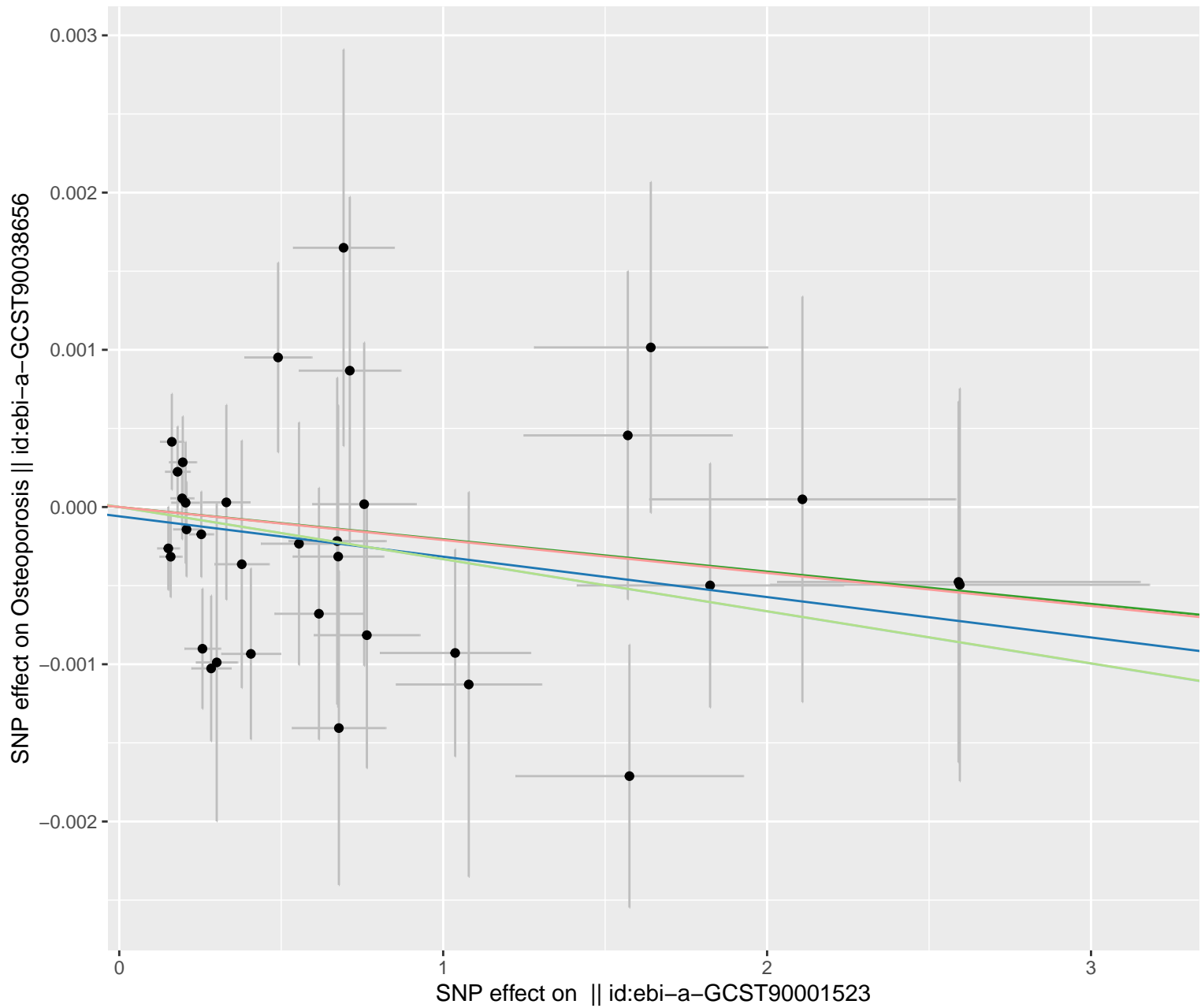

Supplement: Supplementary File 1 — Results of the causal effect of immune cells on osteoporosis. [file DataSheet_1.zip › Supplementary file 1/CD33- HLA DR+ AC.pdf]

# MR Test

- Inverse variance weighted
- MR Egger
- Simple mode
- Weighted median
- Weighted mode

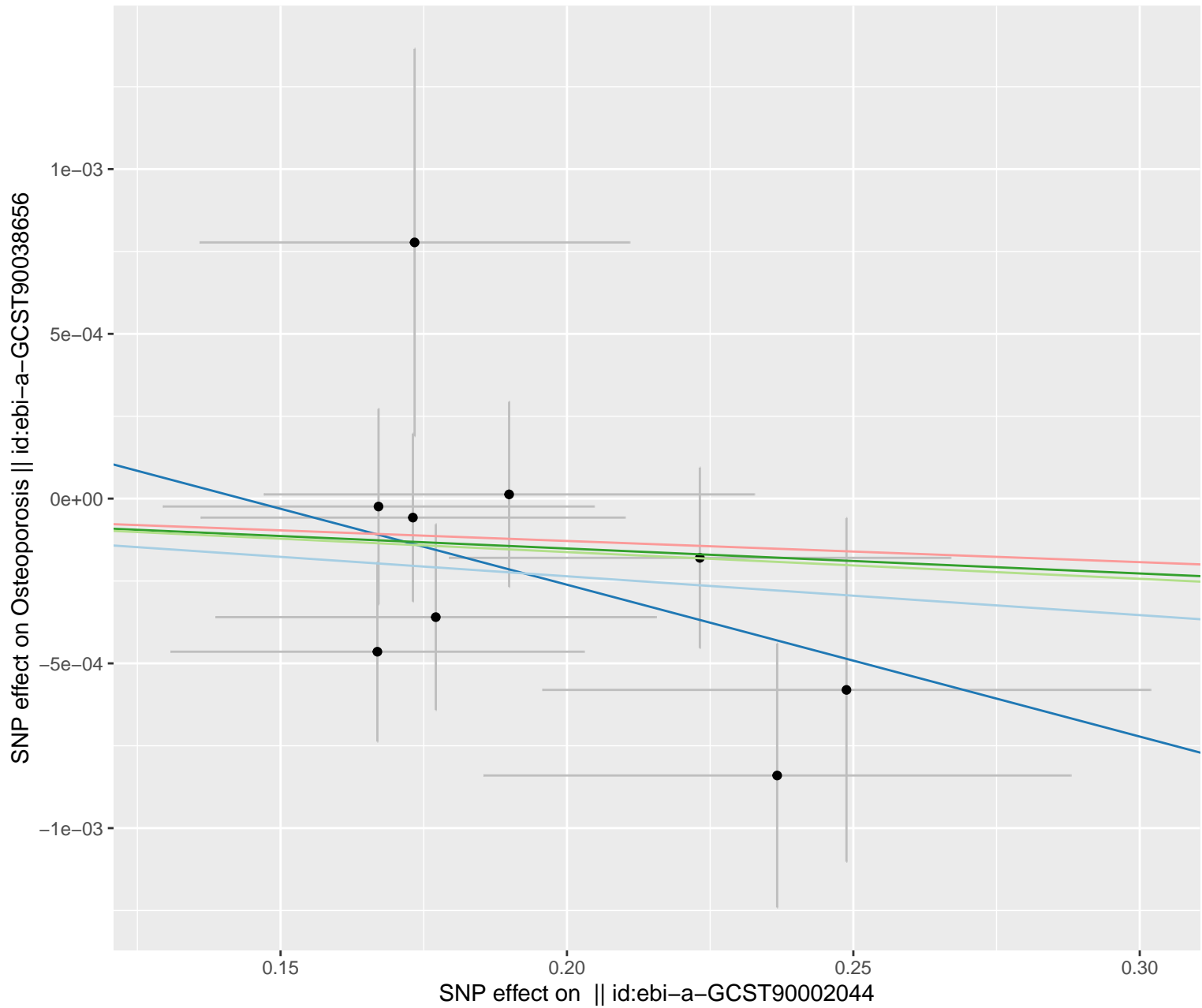

Supplement: Supplementary File 1 — Results of the causal effect of immune cells on osteoporosis. [file DataSheet_1.zip › Supplementary file 1/CD45 on CD33dim HLA DR+ CD11b-.pdf]

# MR Test

- Inverse variance weighted
- MR Egger
- Simple mode
- Weighted median
- Weighted mode

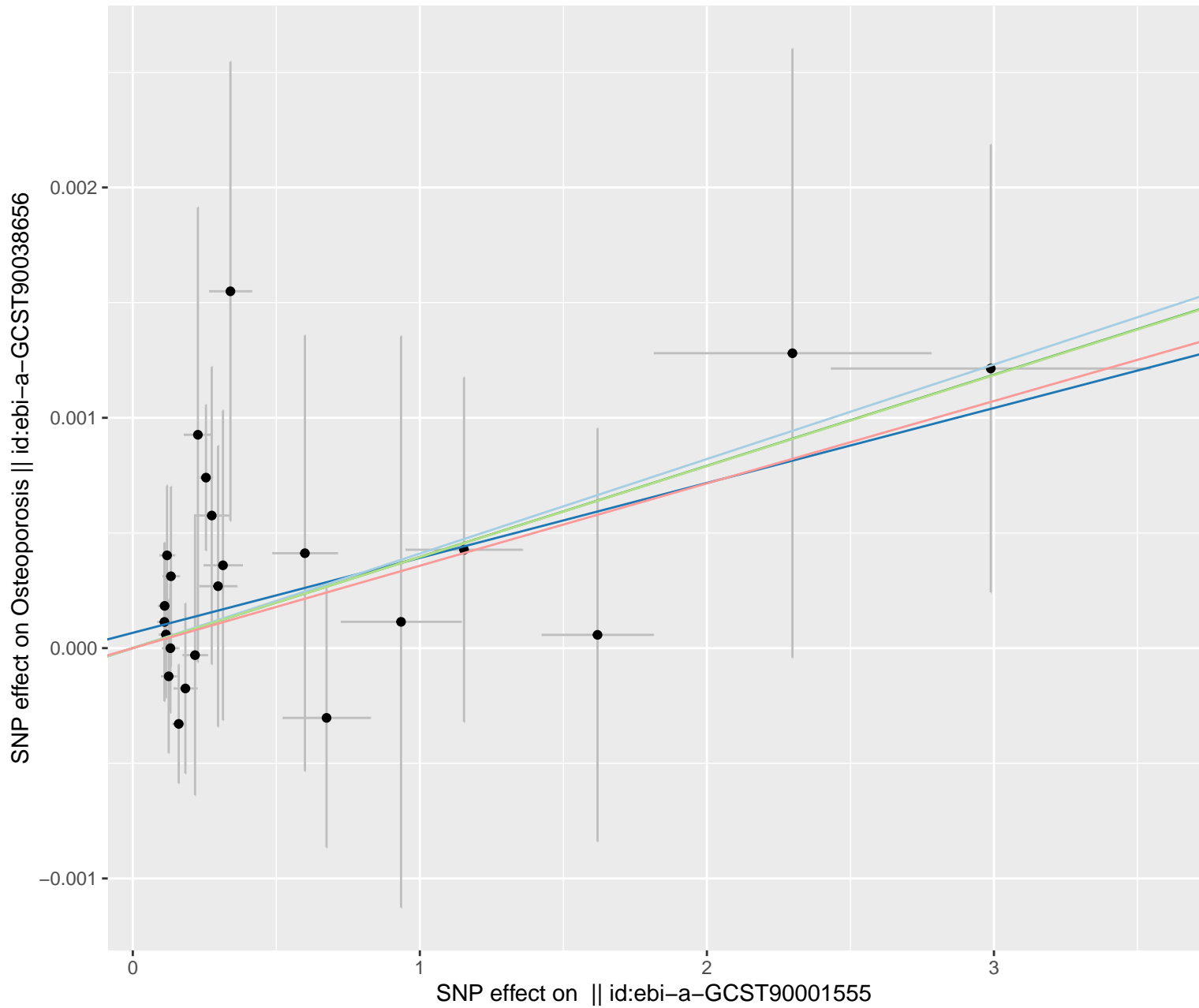

Supplement: Supplementary File 1 — Results of the causal effect of immune cells on osteoporosis. [file DataSheet_1.zip › Supplementary file 1/EM CD8br %CD8brú¿riskú⌐.pdf]

## MR Test

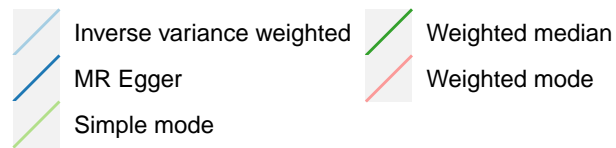

SNP effect on Osteoporosis || id:ebi-a-GCST90038656

SNP effect on || id:ebi-a-GCST90002106

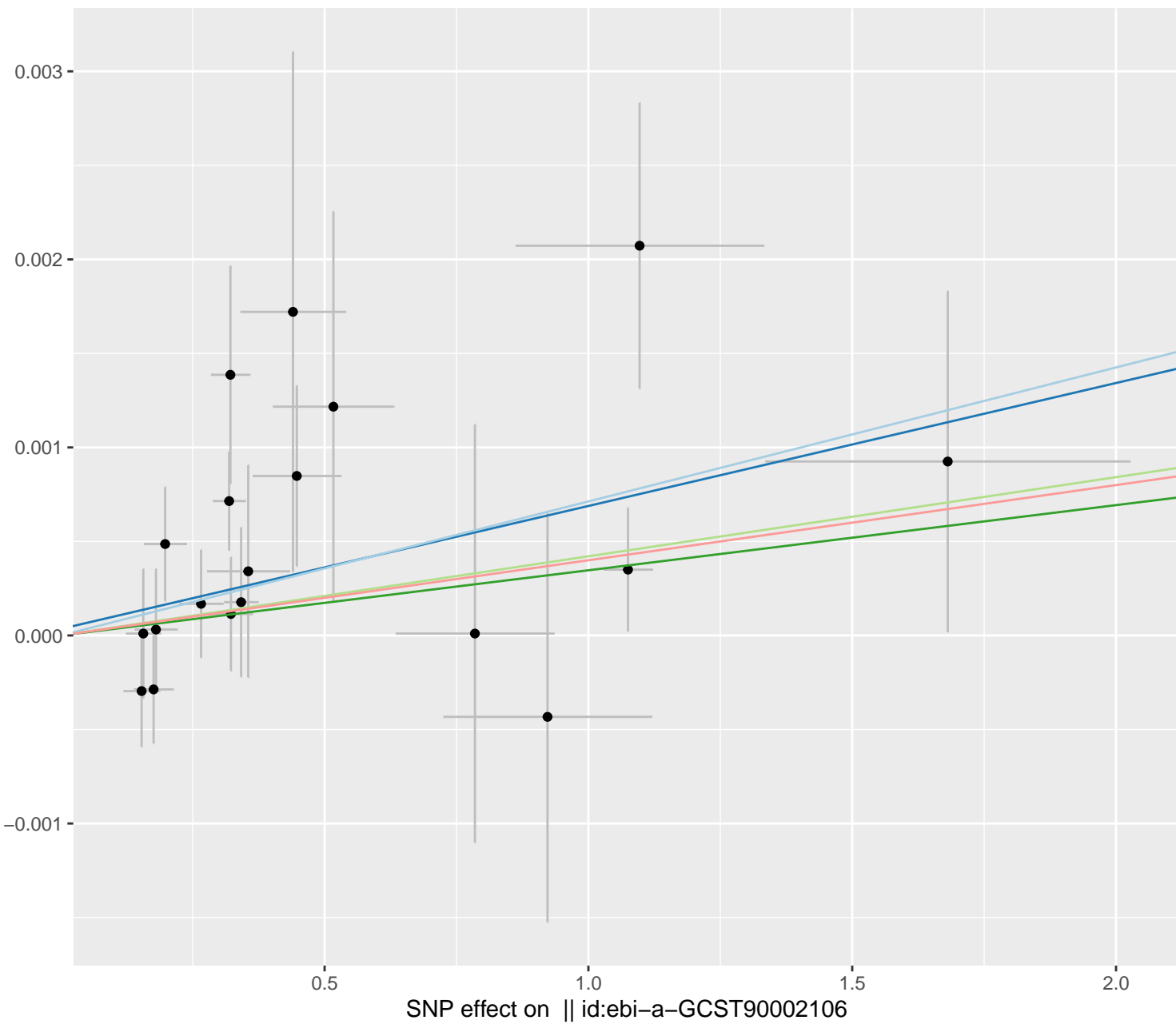

Supplement: Supplementary File 1 — Results of the causal effect of immune cells on osteoporosis. [file DataSheet_1.zip › Supplementary file 1/HLA DR on DCú¿riskú⌐.pdf]

## MR Test

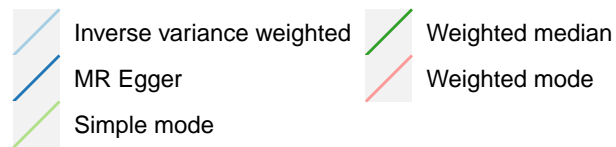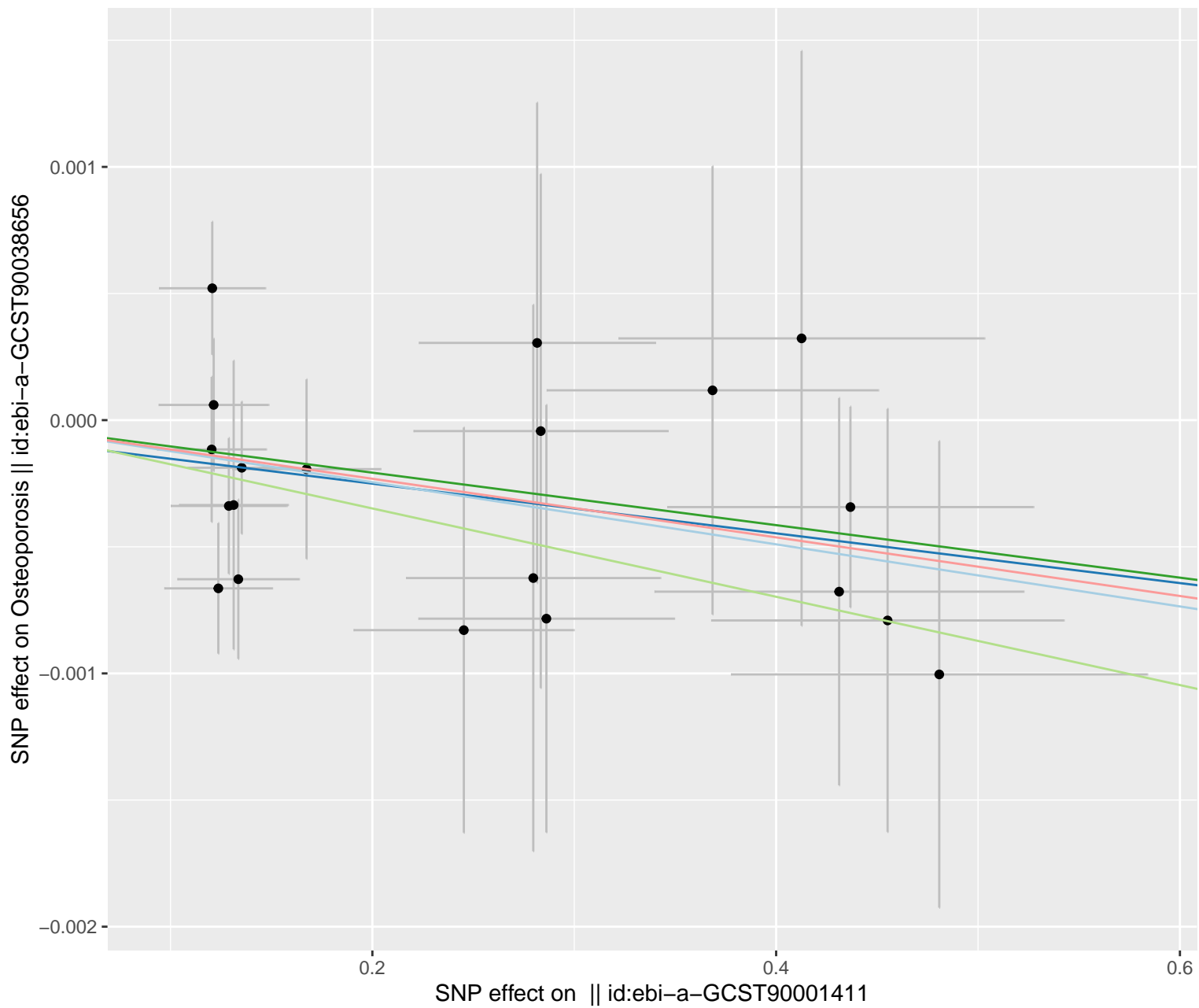

Supplement: Supplementary File 1 — Results of the causal effect of immune cells on osteoporosis. [file DataSheet_1.zip › Supplementary file 1/IgD+ CD24+ %B cell.pdf]

## MR Test

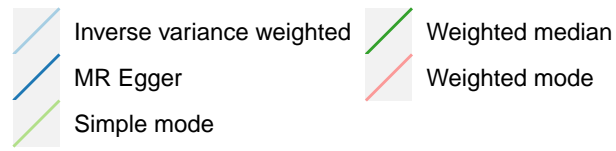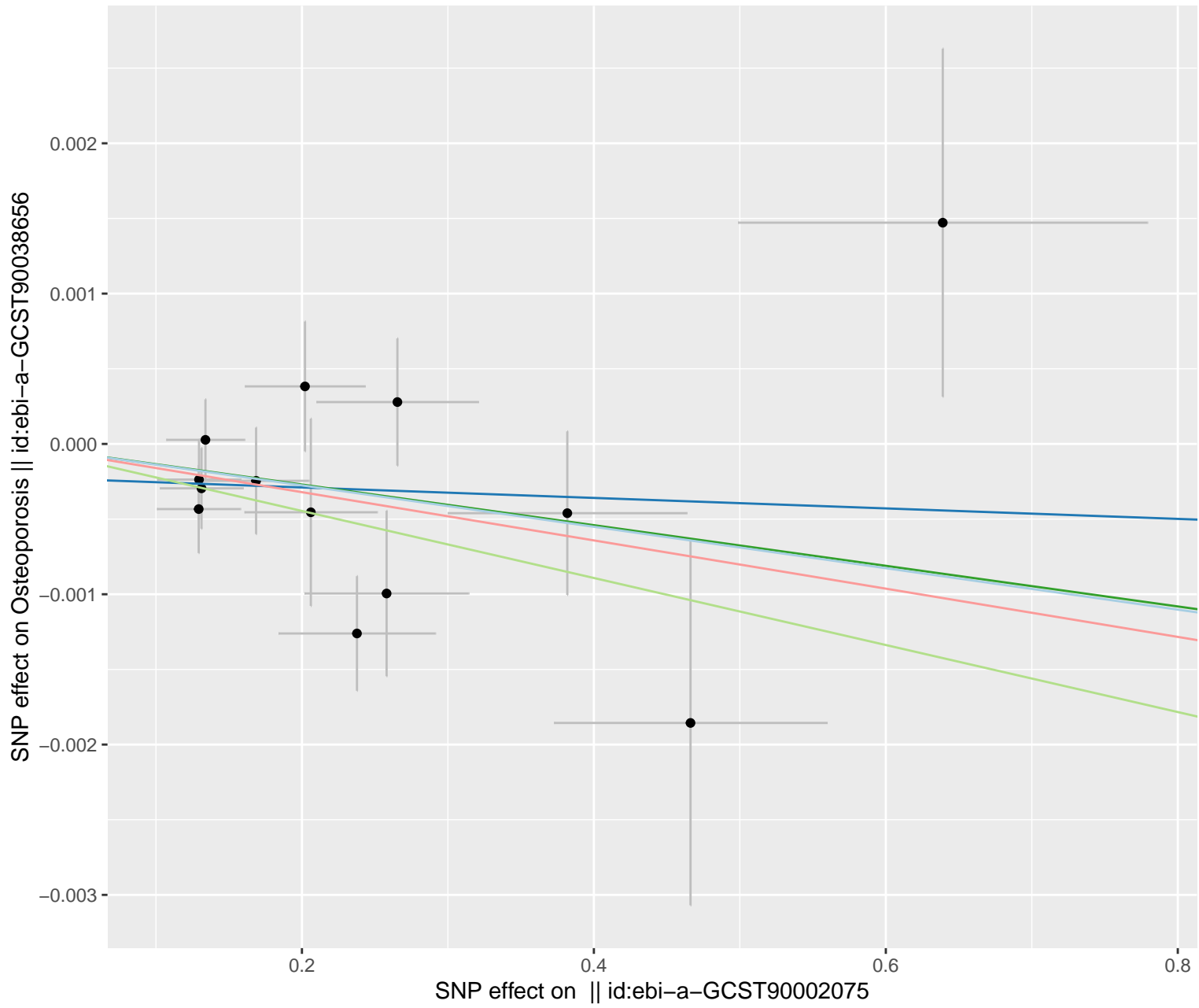

Supplement: Supplementary File 1 — Results of the causal effect of immune cells on osteoporosis. [file DataSheet_1.zip › Supplementary file 1/SSC-A on B cell.pdf]

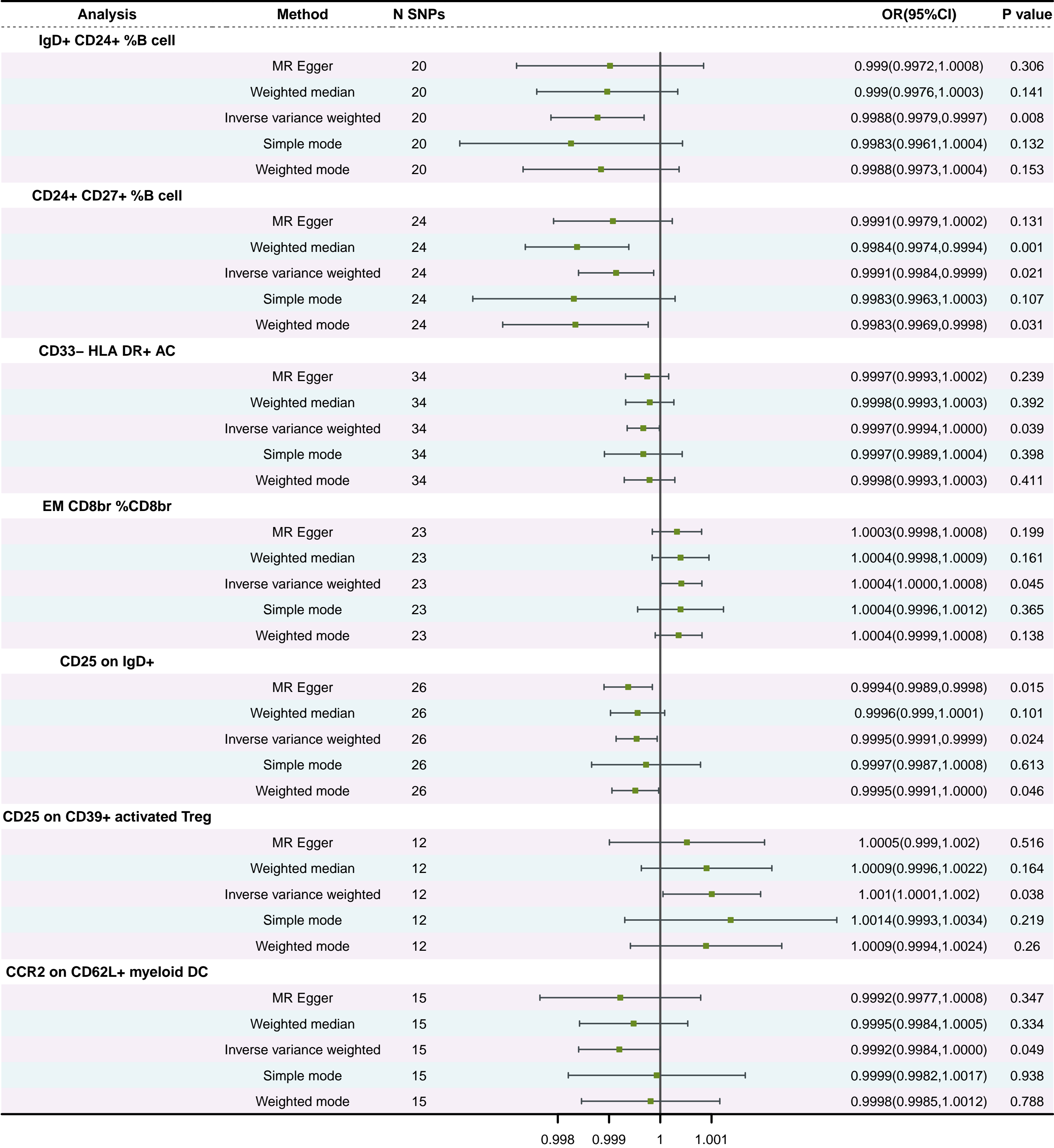

Supplement: Supplementary File 2 — Sensitivity analysis results of immune cells on osteoporosis. [file DataSheet_2.zip › Supplementary file 2/forest1.pdf]

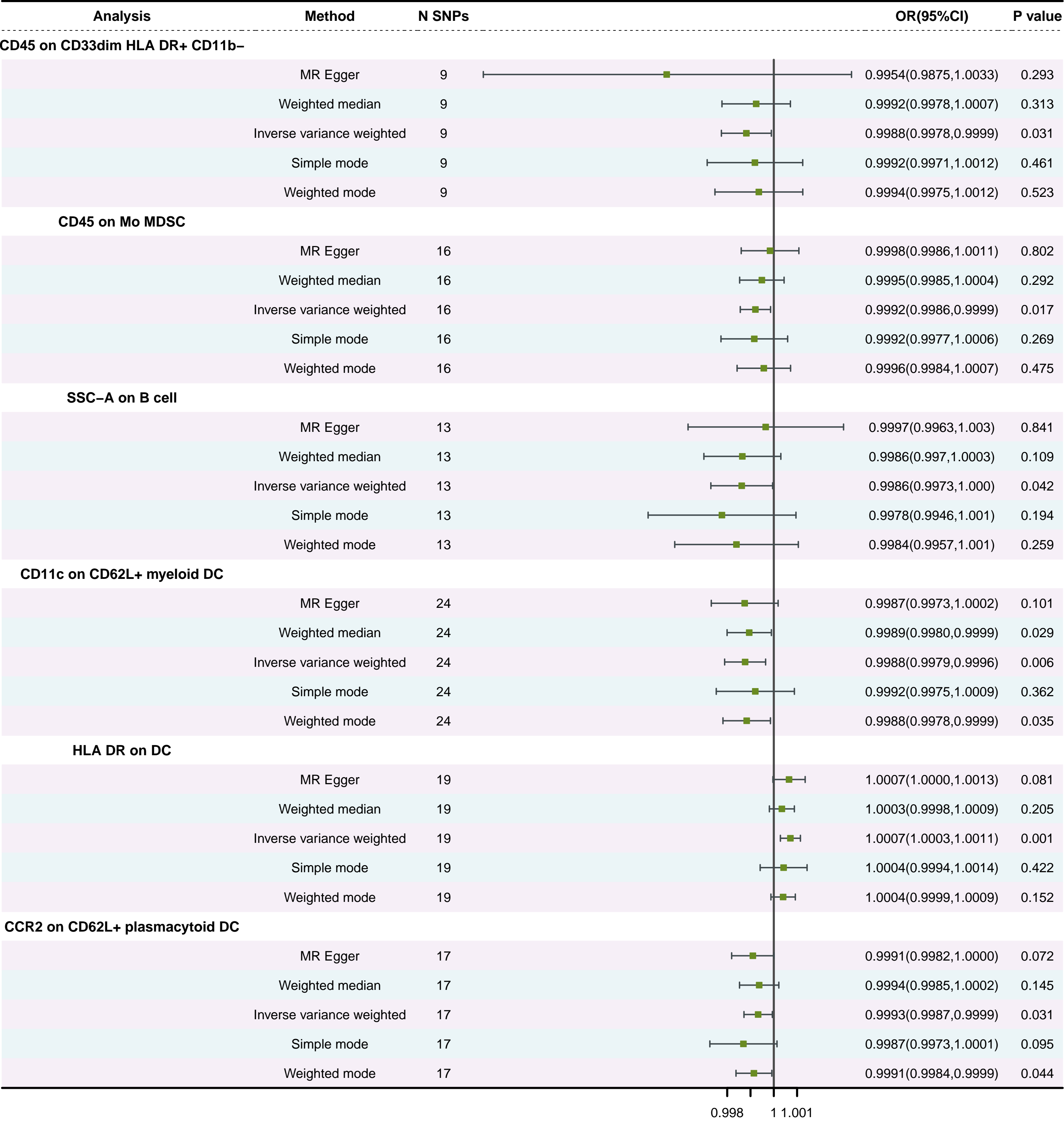

Supplement: Supplementary File 2 — Sensitivity analysis results of immune cells on osteoporosis. [file DataSheet_2.zip › Supplementary file 2/forest2.pdf]

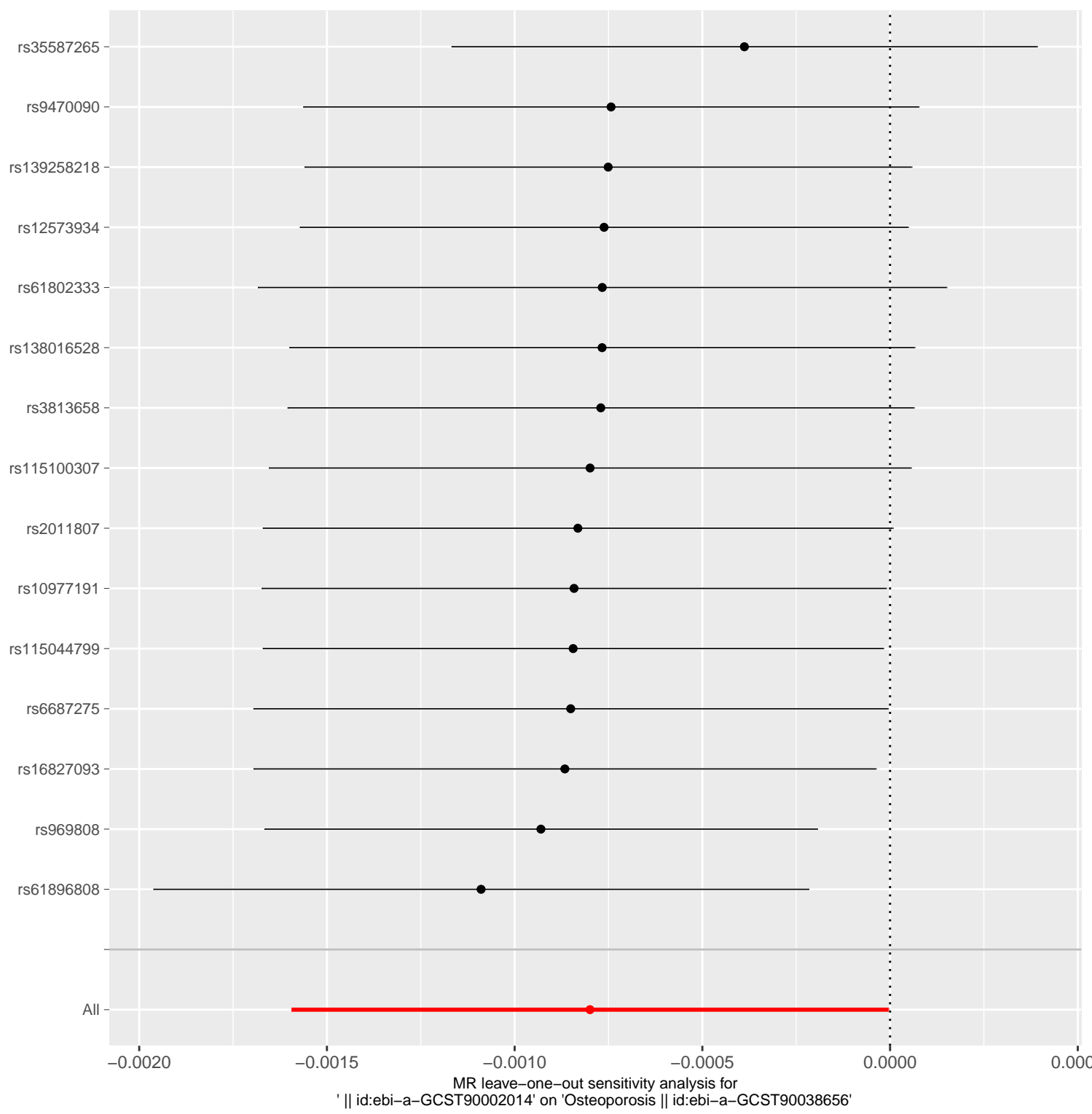

Supplement: Supplementary File 3 — Scatterplots of immune cells on osteoporosis. [file DataSheet_3.zip › Supplementary file 3/CCR2 on CD62L+ myeloid DC/sensitivity-analysis.pdf]

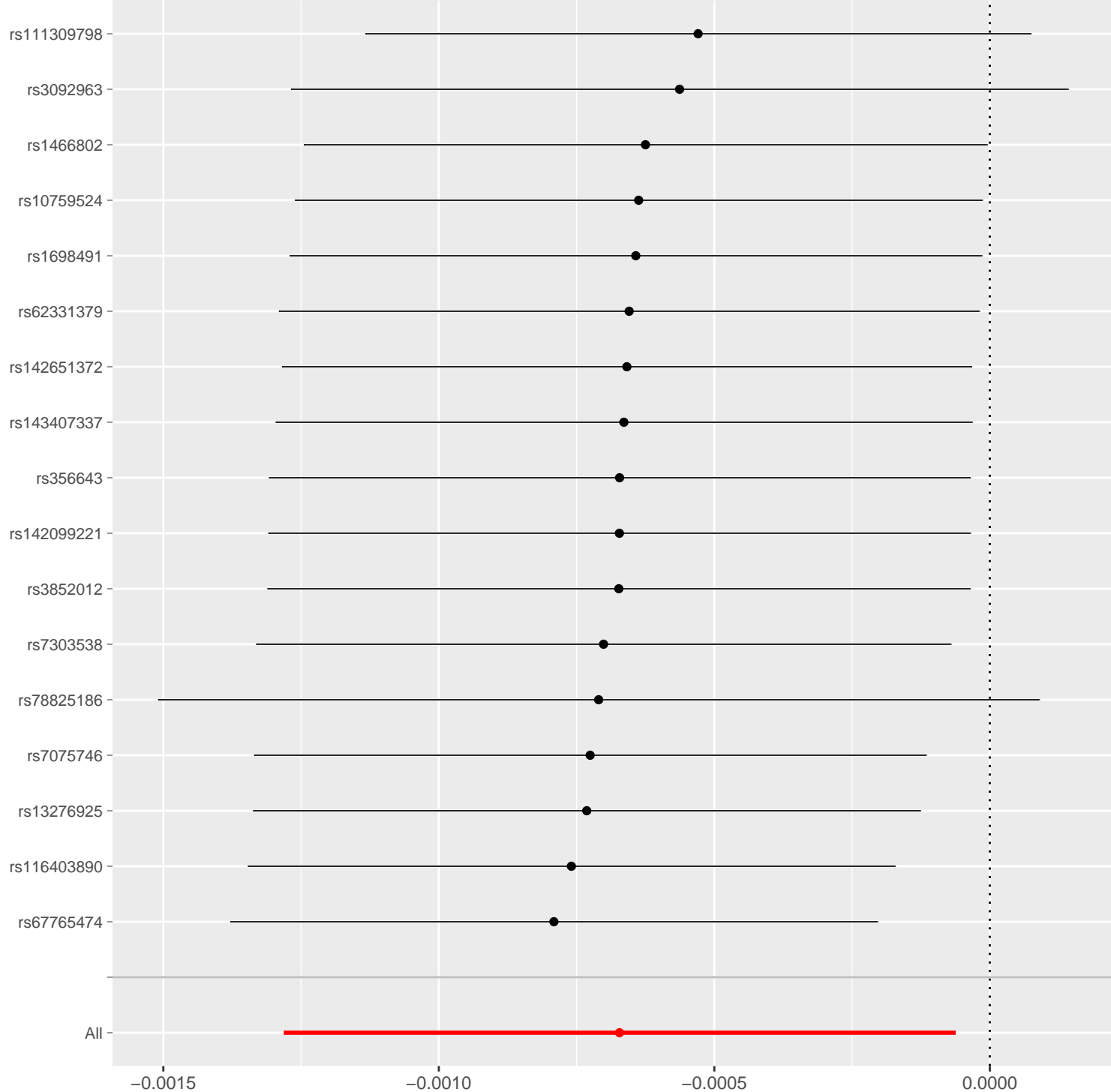

MR leave-one-out sensitivity analysis for  
' || id:ebi-a-GCST90002016' on 'Osteoporosis || id:ebi-a-GCST90038656'

Supplement: Supplementary File 3 — Scatterplots of immune cells on osteoporosis. [file DataSheet_3.zip › Supplementary file 3/CCR2 on CD62L+ plasmacytoid DC/sensitivity-analysis.pdf]

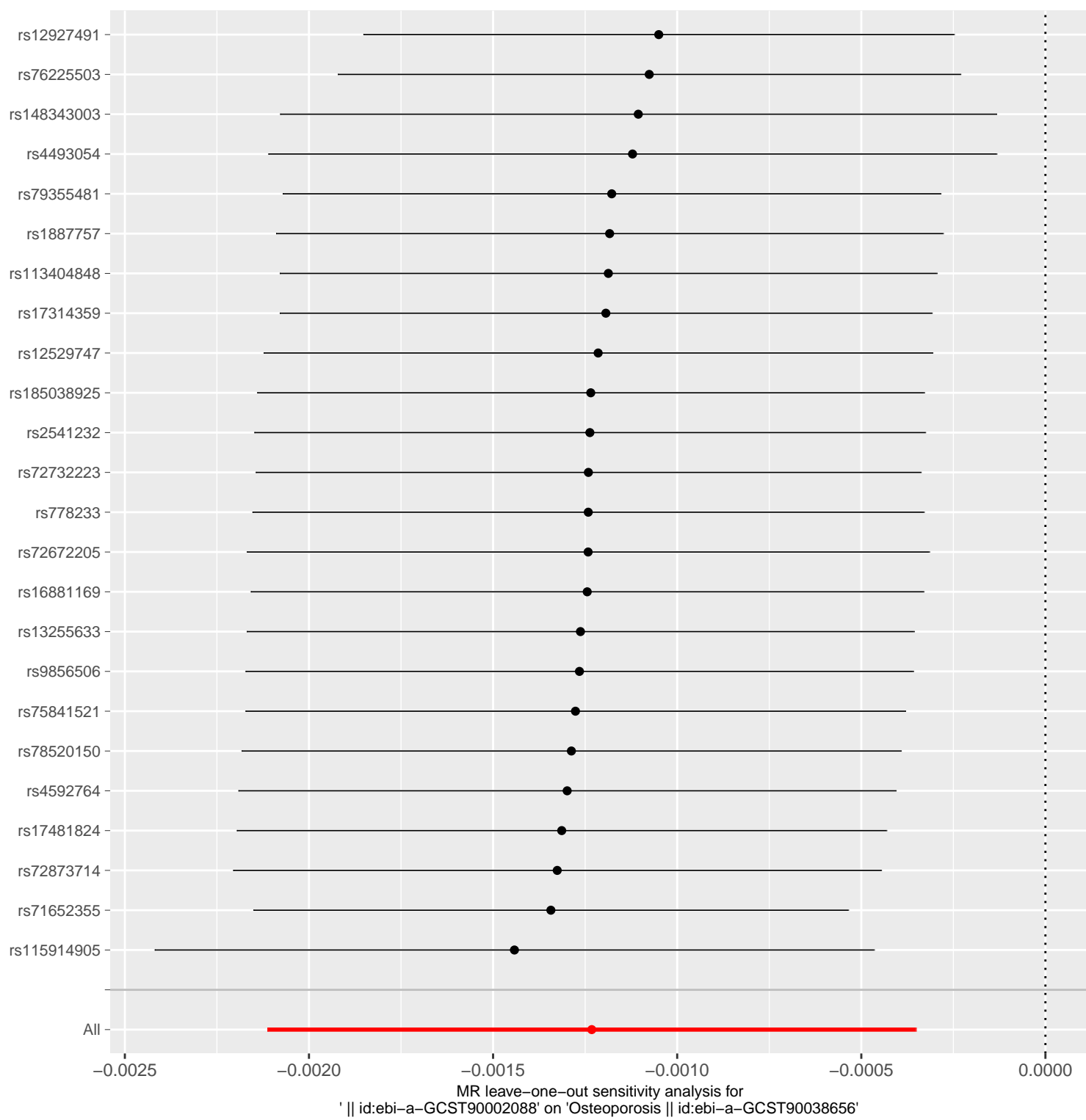

Supplement: Supplementary File 3 — Scatterplots of immune cells on osteoporosis. [file DataSheet_3.zip › Supplementary file 3/CD11c on CD62L+ myeloid DC/sensitivity-analysis.pdf]

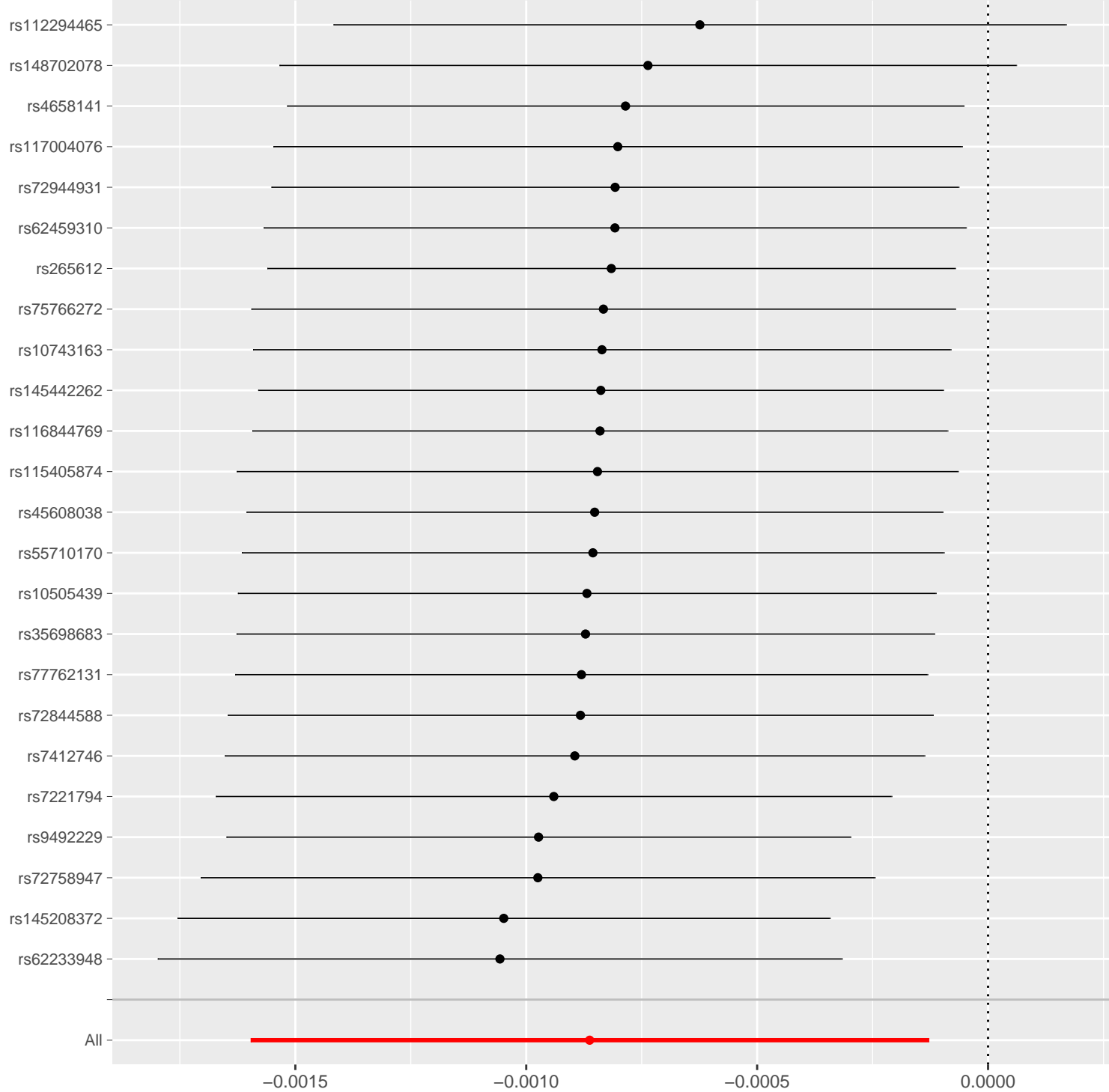

Supplement: Supplementary File 3 — Scatterplots of immune cells on osteoporosis. [file DataSheet_3.zip › Supplementary file 3/CD24+ CD27+ %B cell/sensitivity-analysis.pdf]

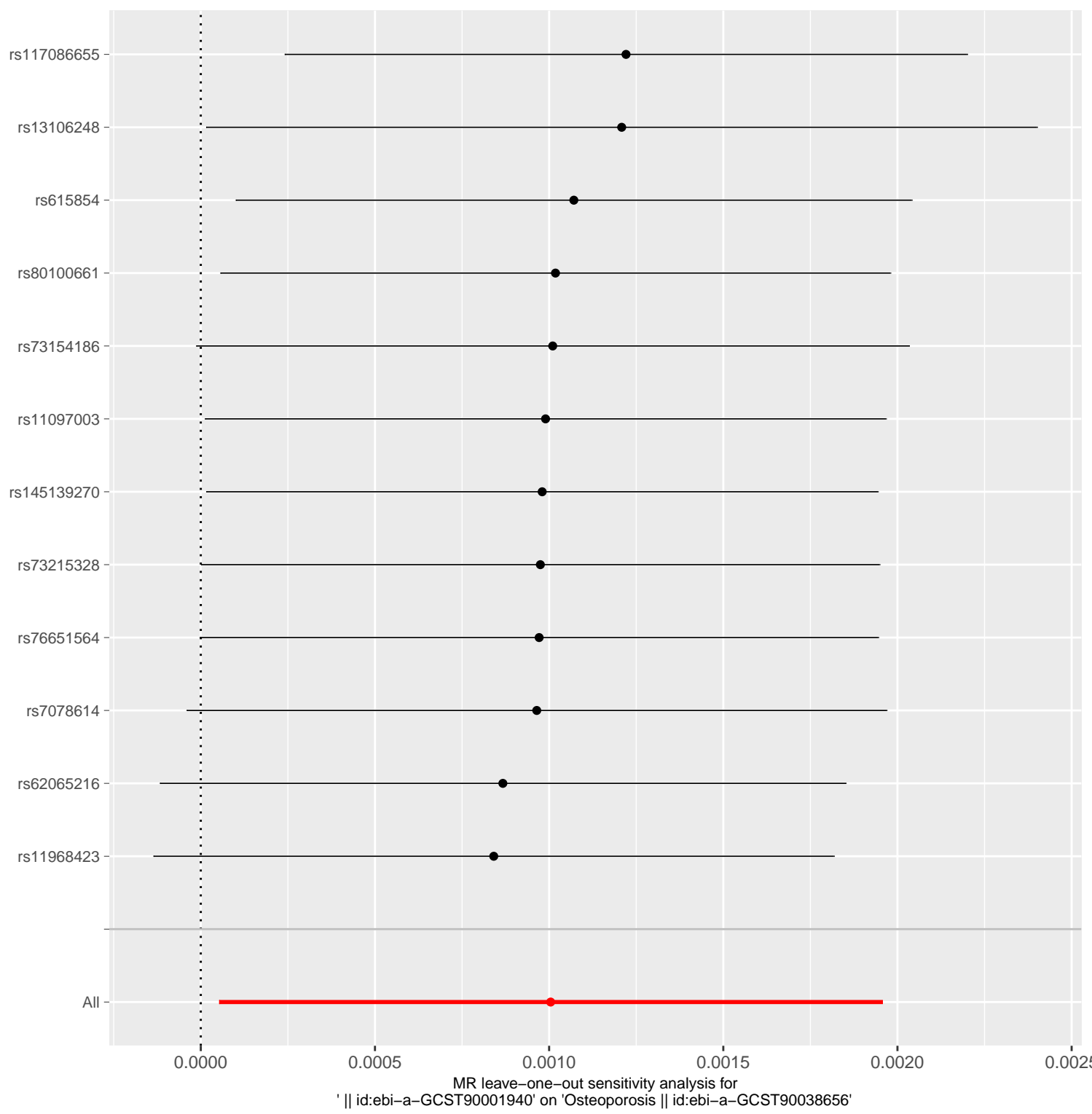

Supplement: Supplementary File 3 — Scatterplots of immune cells on osteoporosis. [file DataSheet_3.zip › Supplementary file 3/CD25 on CD39+ activated Tregú¿riskú⌐/sensitivity-analysis.pdf]

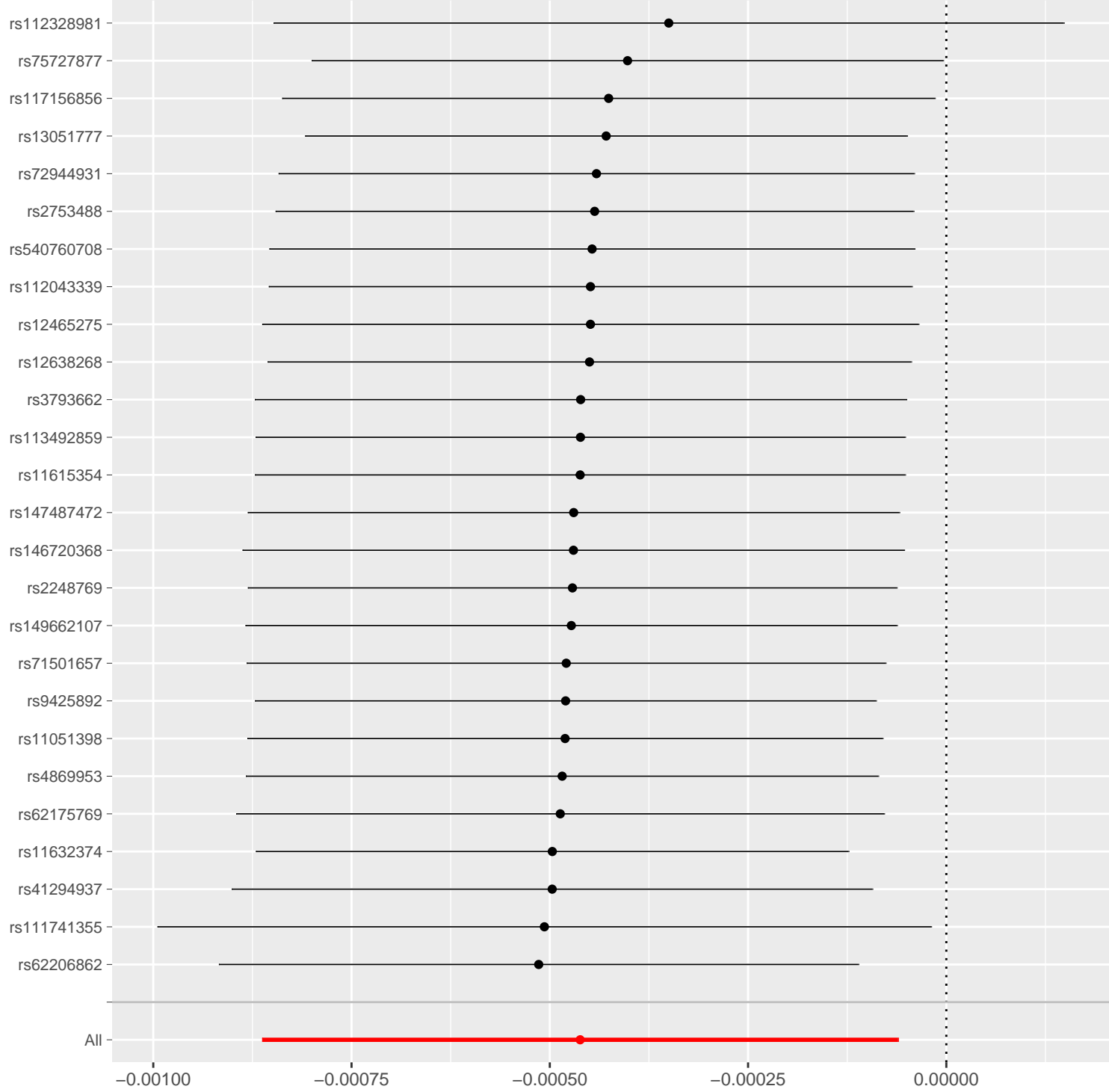

Supplement: Supplementary File 3 — Scatterplots of immune cells on osteoporosis. [file DataSheet_3.zip › Supplementary file 3/CD25 on IgD+/sensitivity-analysis.pdf]

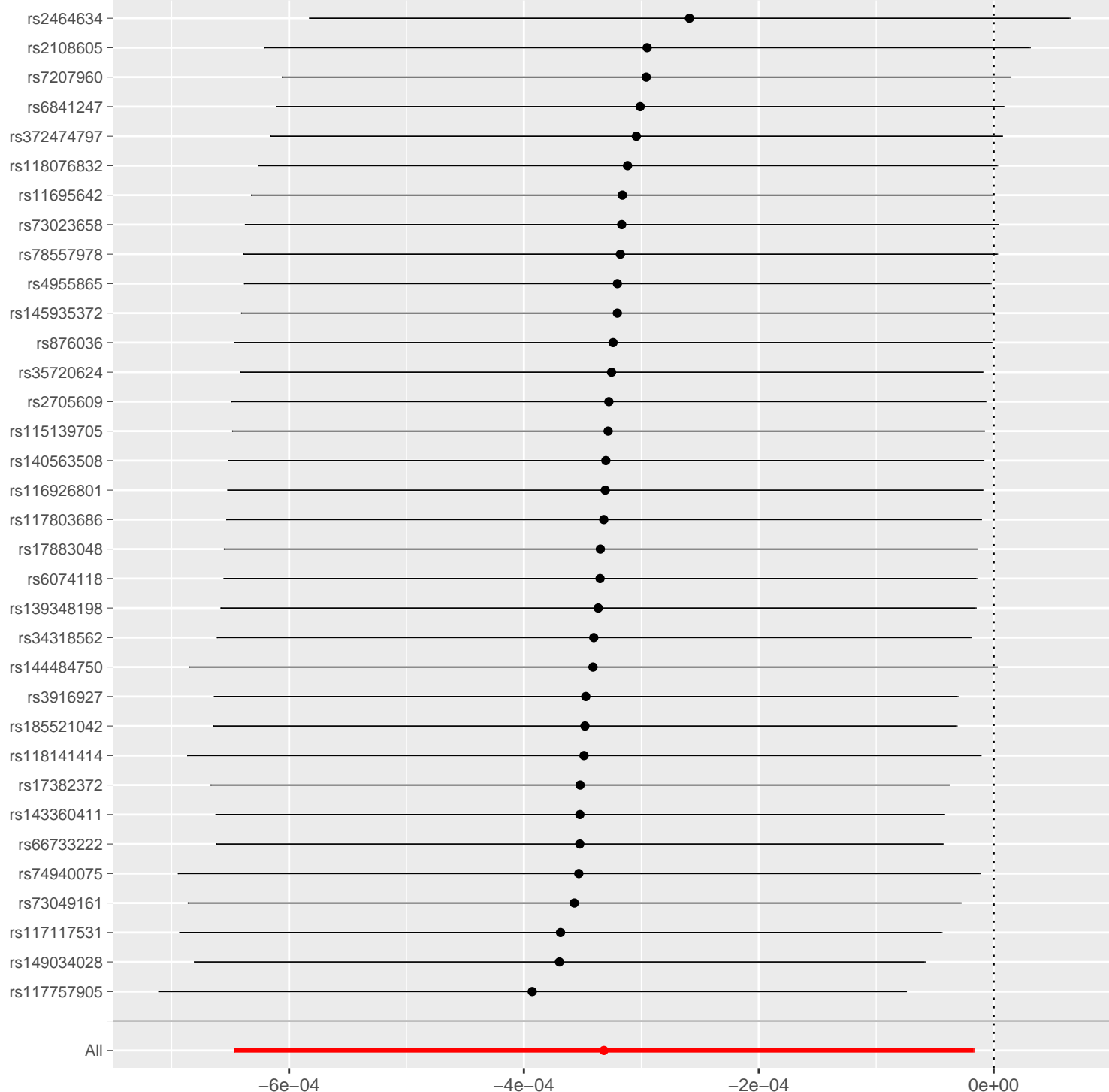

Supplement: Supplementary File 3 — Scatterplots of immune cells on osteoporosis. [file DataSheet_3.zip › Supplementary file 3/CD33- HLA DR+ AC/sensitivity-analysis.pdf]

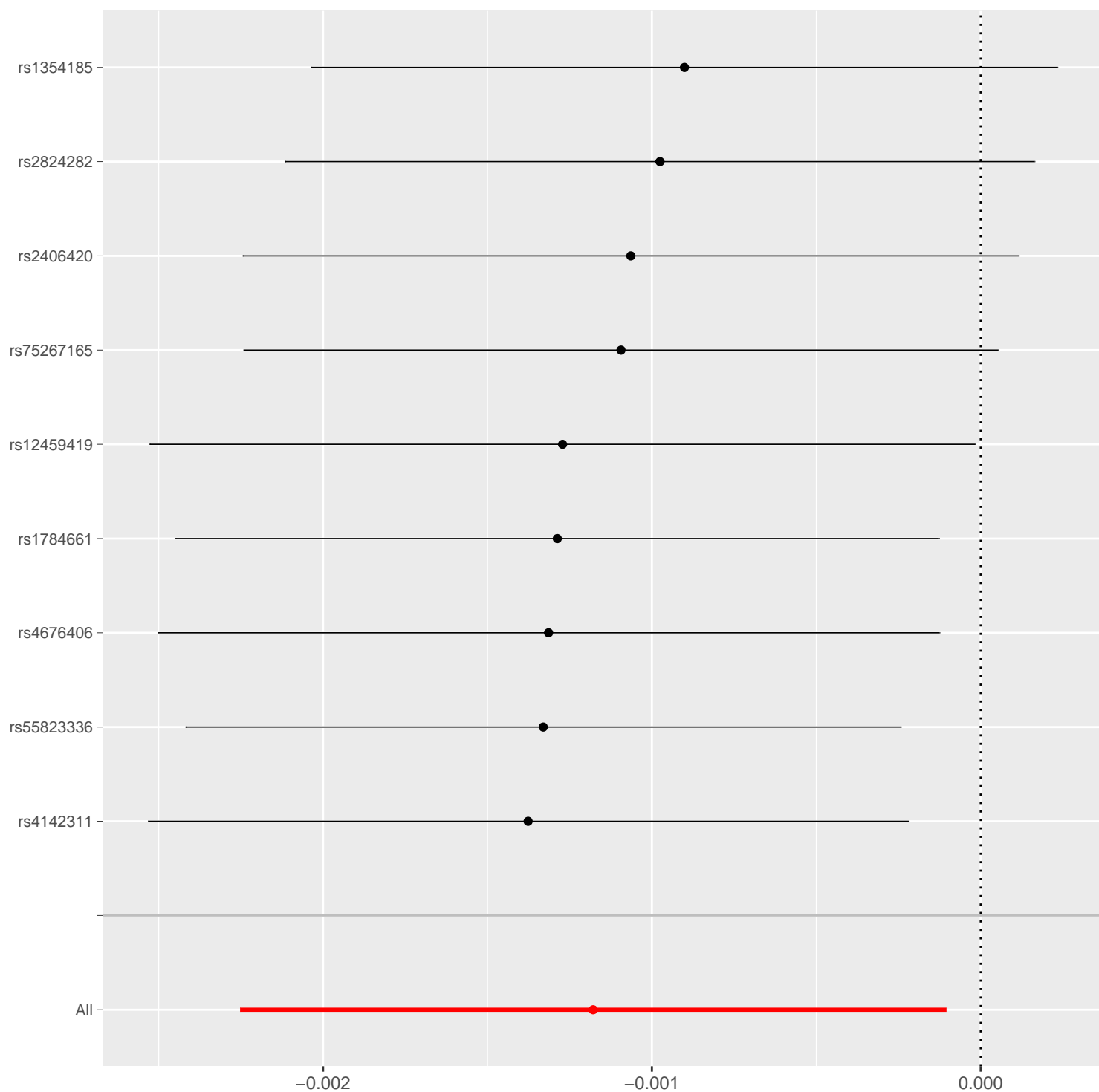

Supplement: Supplementary File 3 — Scatterplots of immune cells on osteoporosis. [file DataSheet_3.zip › Supplementary file 3/CD45 on CD33dim HLA DR+ CD11b-/sensitivity-analysis.pdf]

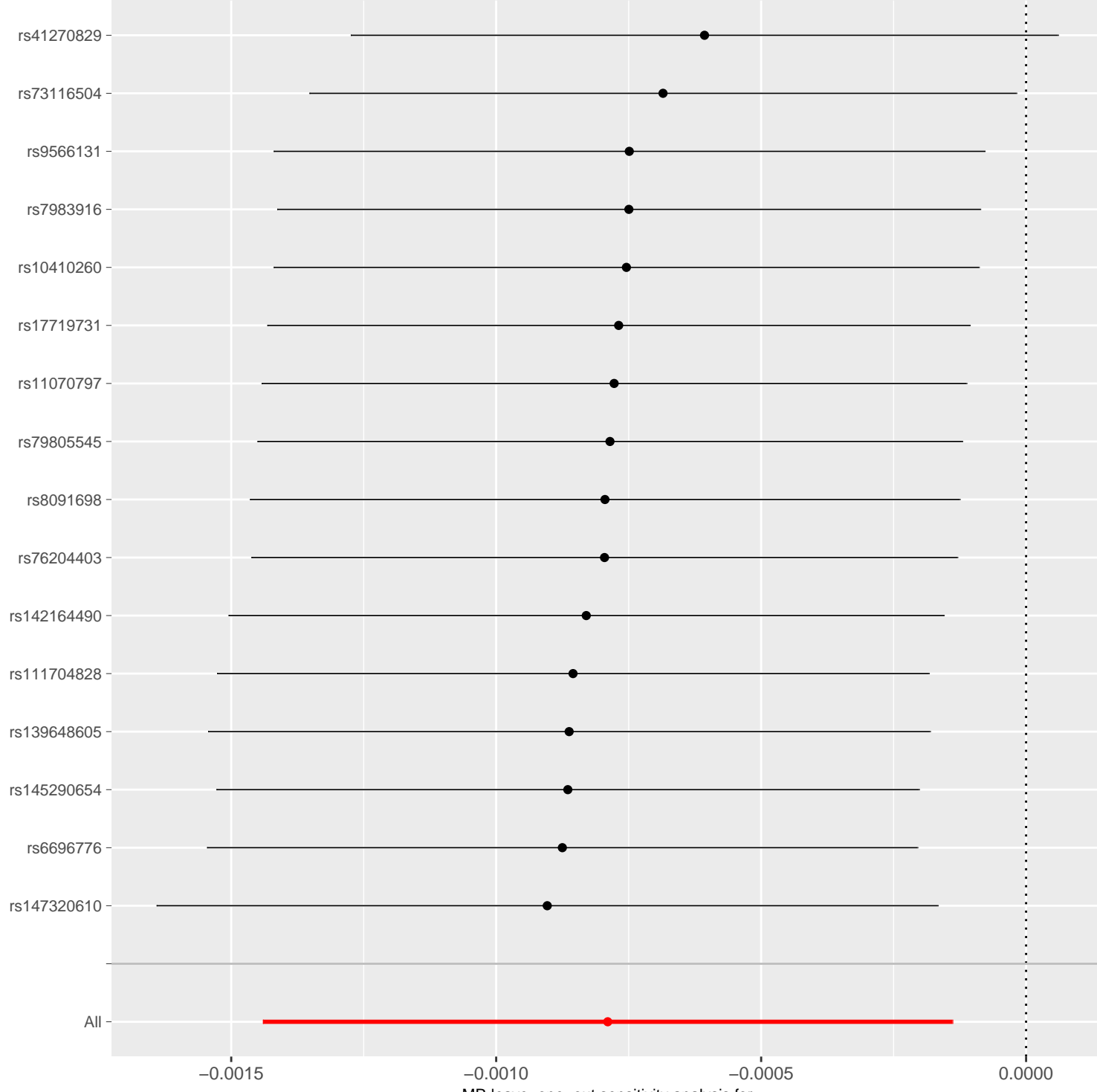

MR leave-one-out sensitivity analysis for  
' || id:ebi-a-GCST90002049' on 'Osteoporosis || id:ebi-a-GCST90038656'

Supplement: Supplementary File 3 — Scatterplots of immune cells on osteoporosis. [file DataSheet_3.zip › Supplementary file 3/CD45 on Mo MDSC/sensitivity-analysis.pdf]

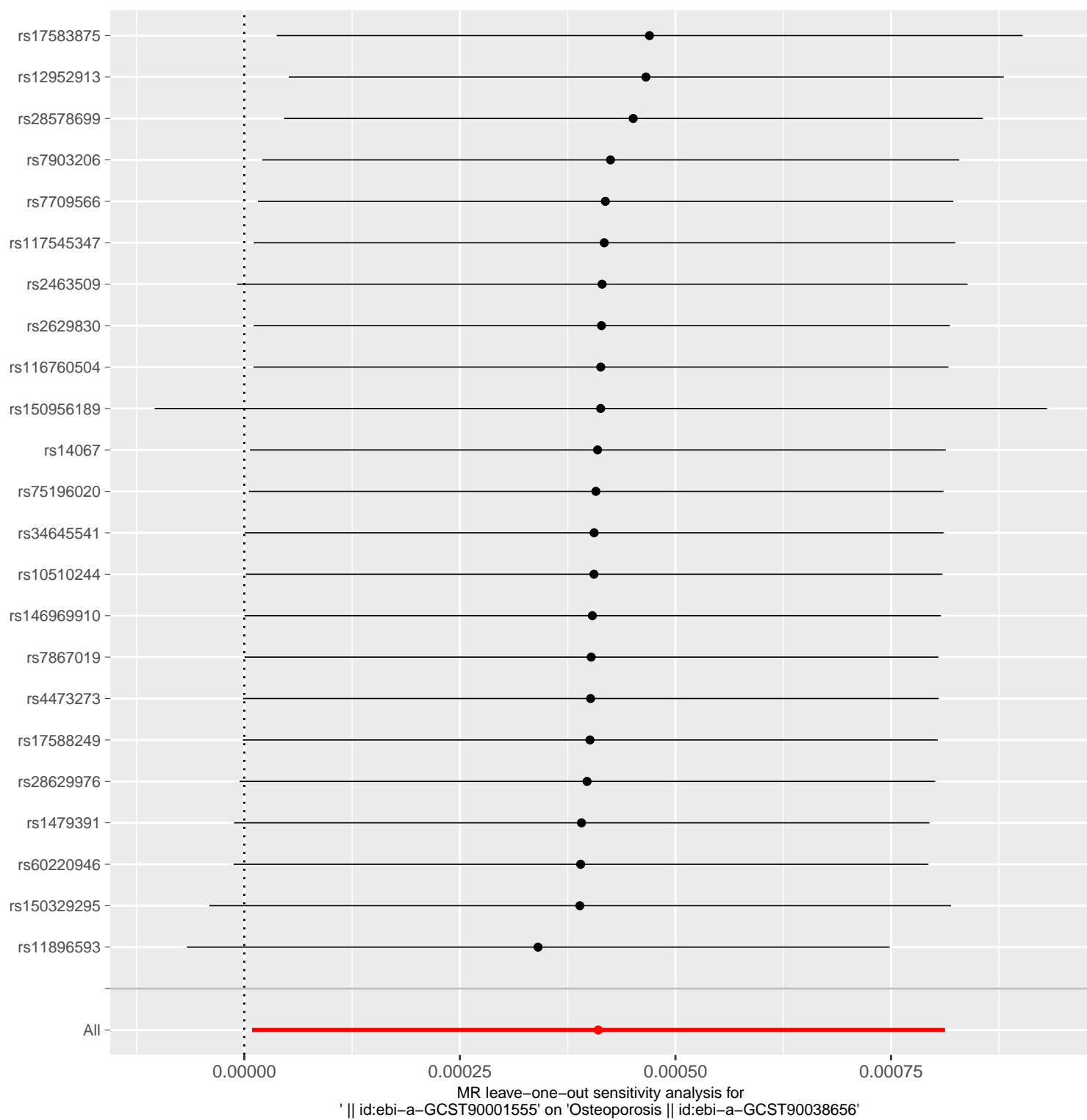

Supplement: Supplementary File 3 — Scatterplots of immune cells on osteoporosis. [file DataSheet_3.zip › Supplementary file 3/EM CD8br %CD8brú¿riskú⌐/sensitivity-analysis.pdf]

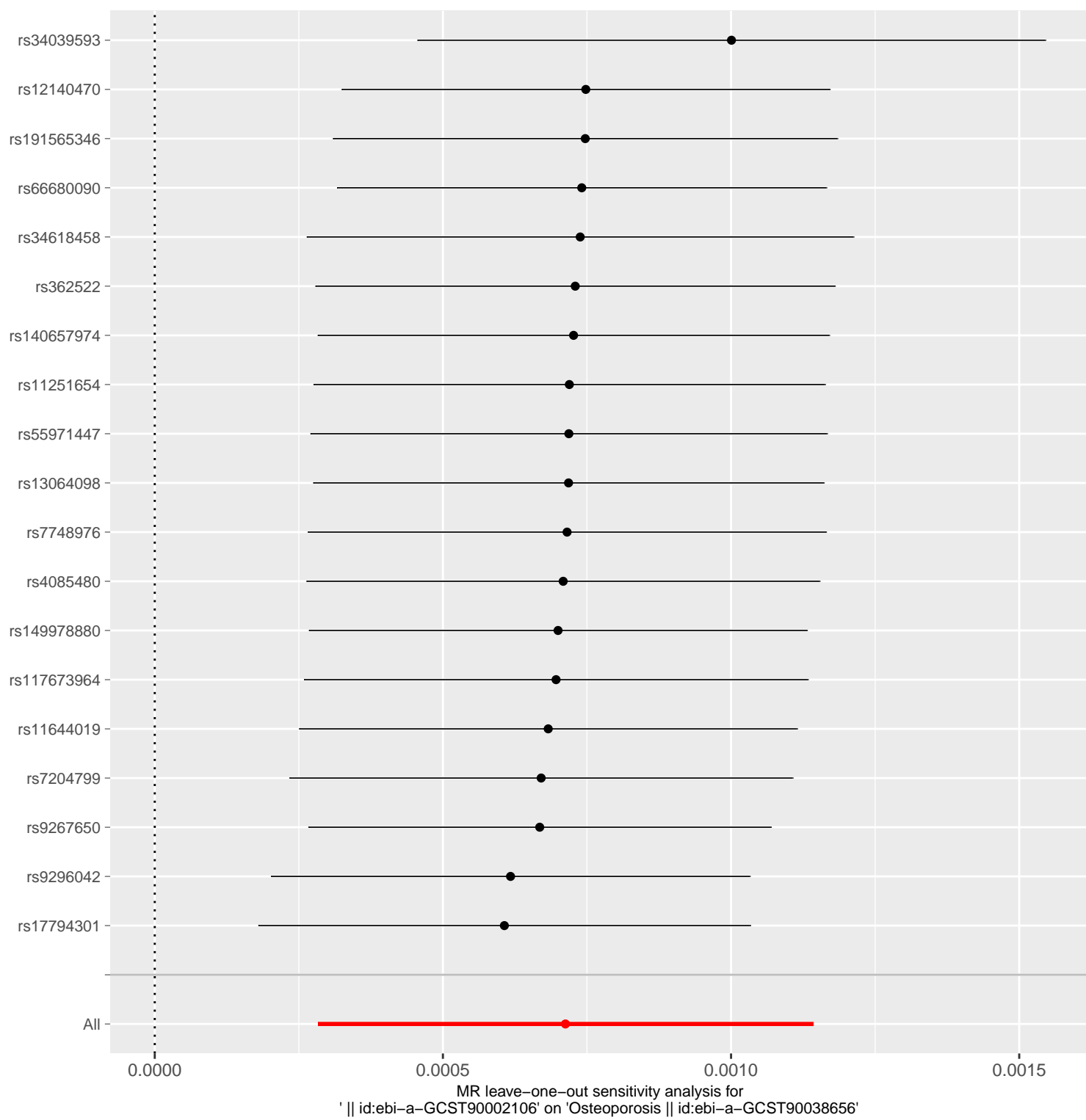

Supplement: Supplementary File 3 — Scatterplots of immune cells on osteoporosis. [file DataSheet_3.zip › Supplementary file 3/HLA DR on DCú¿riskú⌐/sensitivity-analysis.pdf]

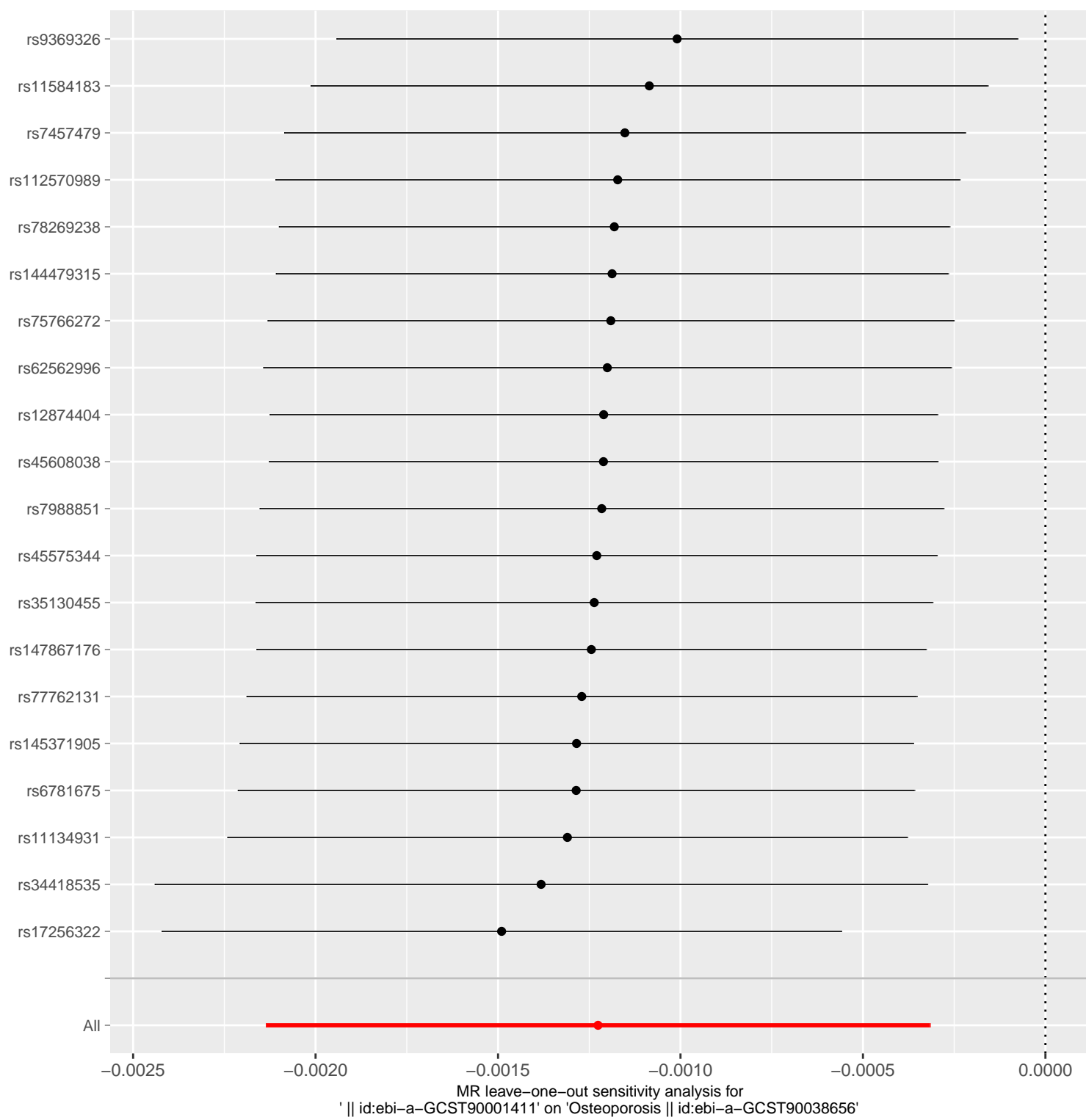

Supplement: Supplementary File 3 — Scatterplots of immune cells on osteoporosis. [file DataSheet_3.zip › Supplementary file 3/IgD+ CD24+ %B cell/sensitivity-analysis.pdf]

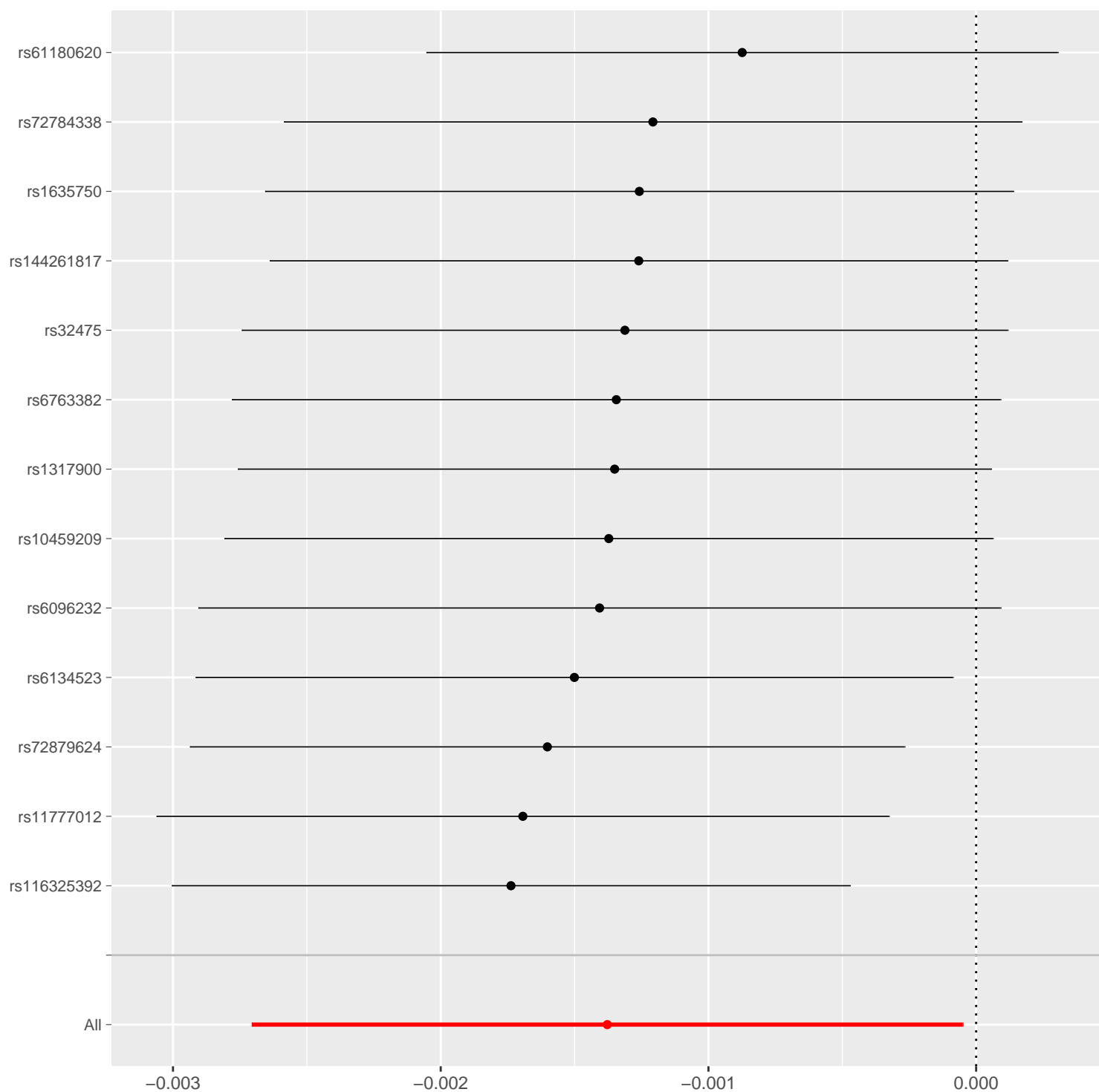

MR leave-one-out sensitivity analysis for  
' || id:ebi-a-GCST90002075' on 'Osteoporosis || id:ebi-a-GCST90038656'

Supplement: Supplementary File 3 — Scatterplots of immune cells on osteoporosis. [file DataSheet_3.zip › Supplementary file 3/SSC-A on B cell/sensitivity-analysis.pdf]

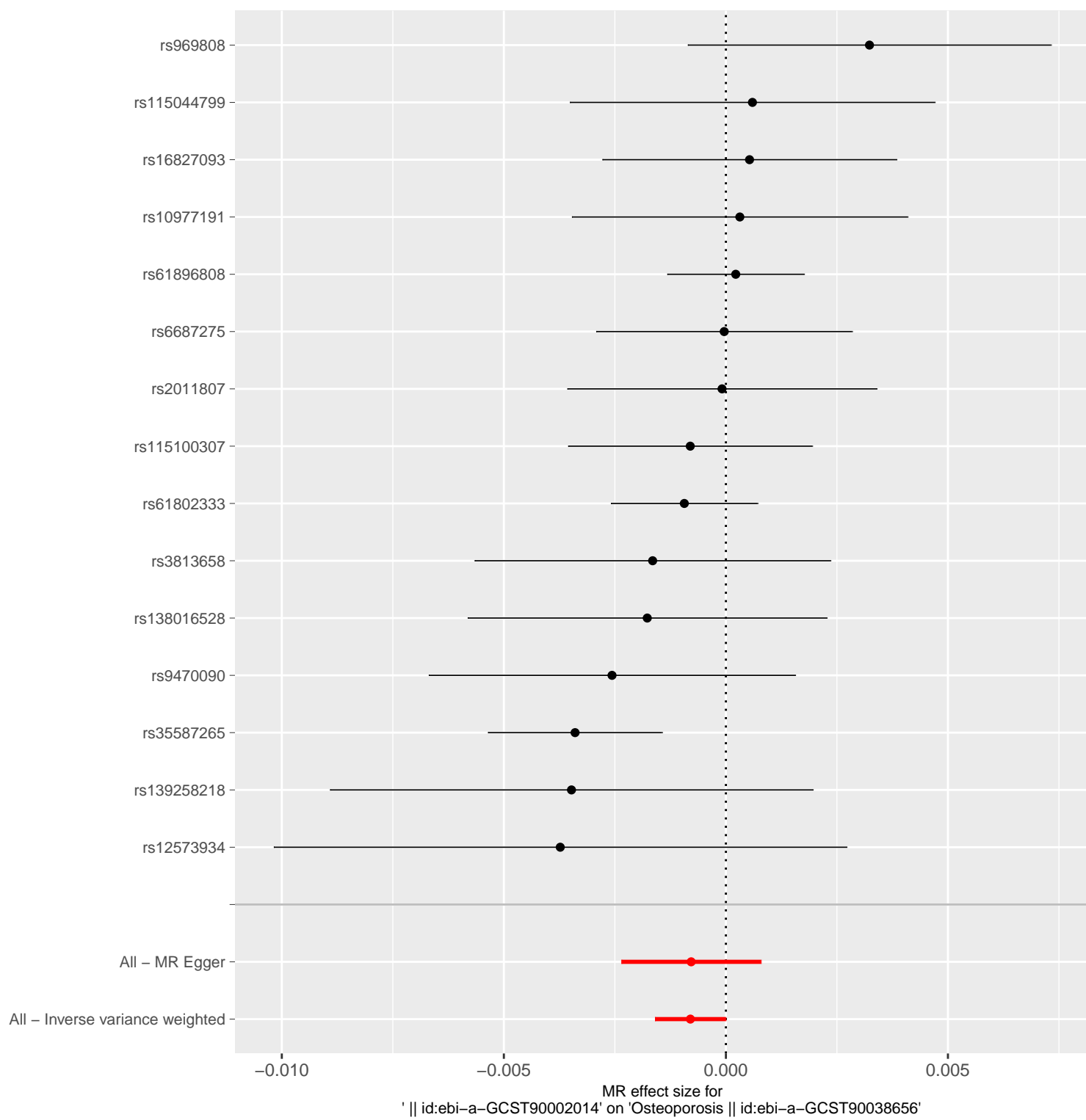

Supplement: Supplementary File 5 — Supplementary figures. [file DataSheet_5.zip › Supplementary file 5/CCR2 on CD62L+ myeloid DC/forest.pdf]

# MR Method

- Inverse variance weighted
- MR Egger

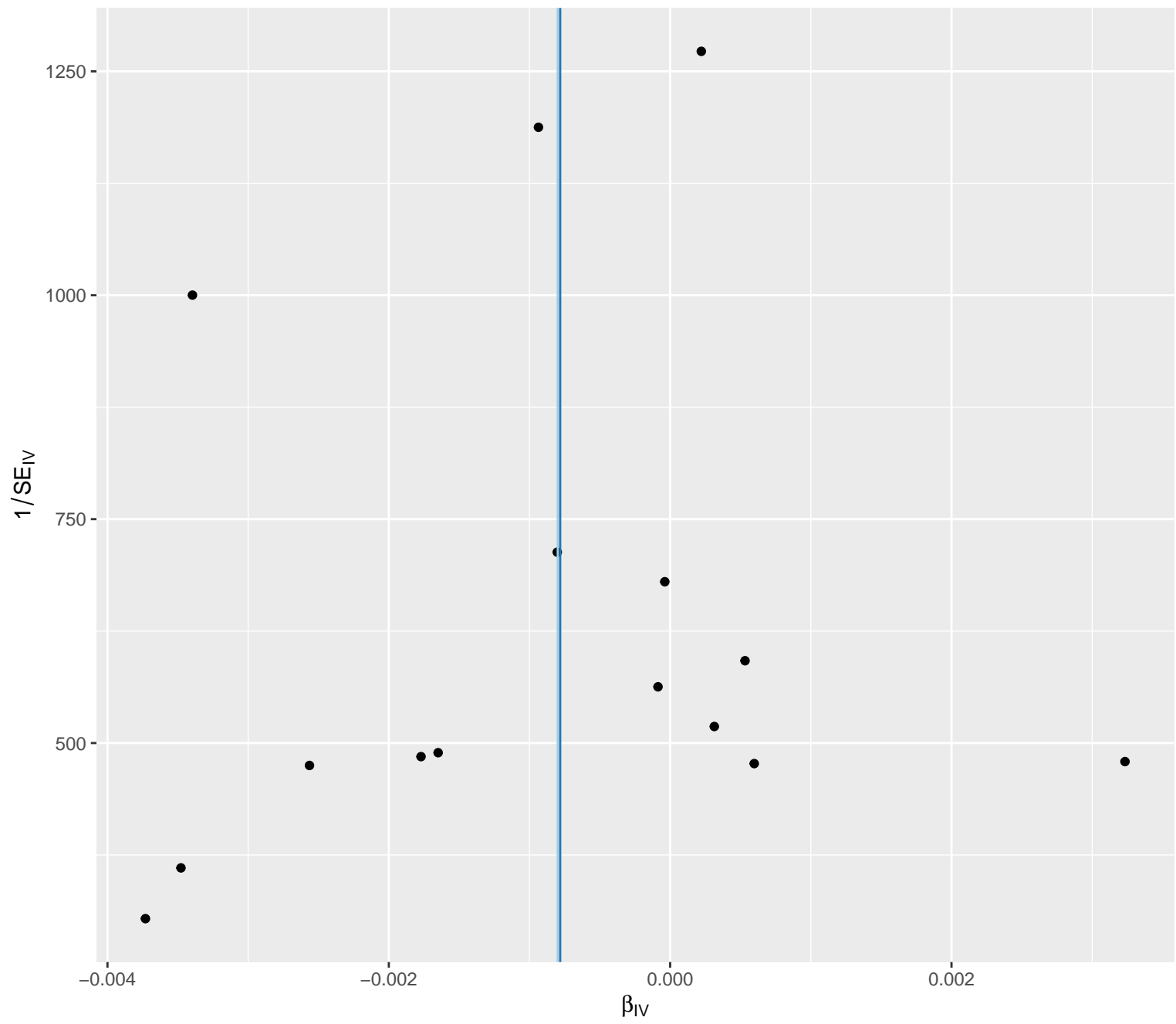

Supplement: Supplementary File 5 — Supplementary figures. [file DataSheet_5.zip › Supplementary file 5/CCR2 on CD62L+ myeloid DC/funnelplot.pdf]

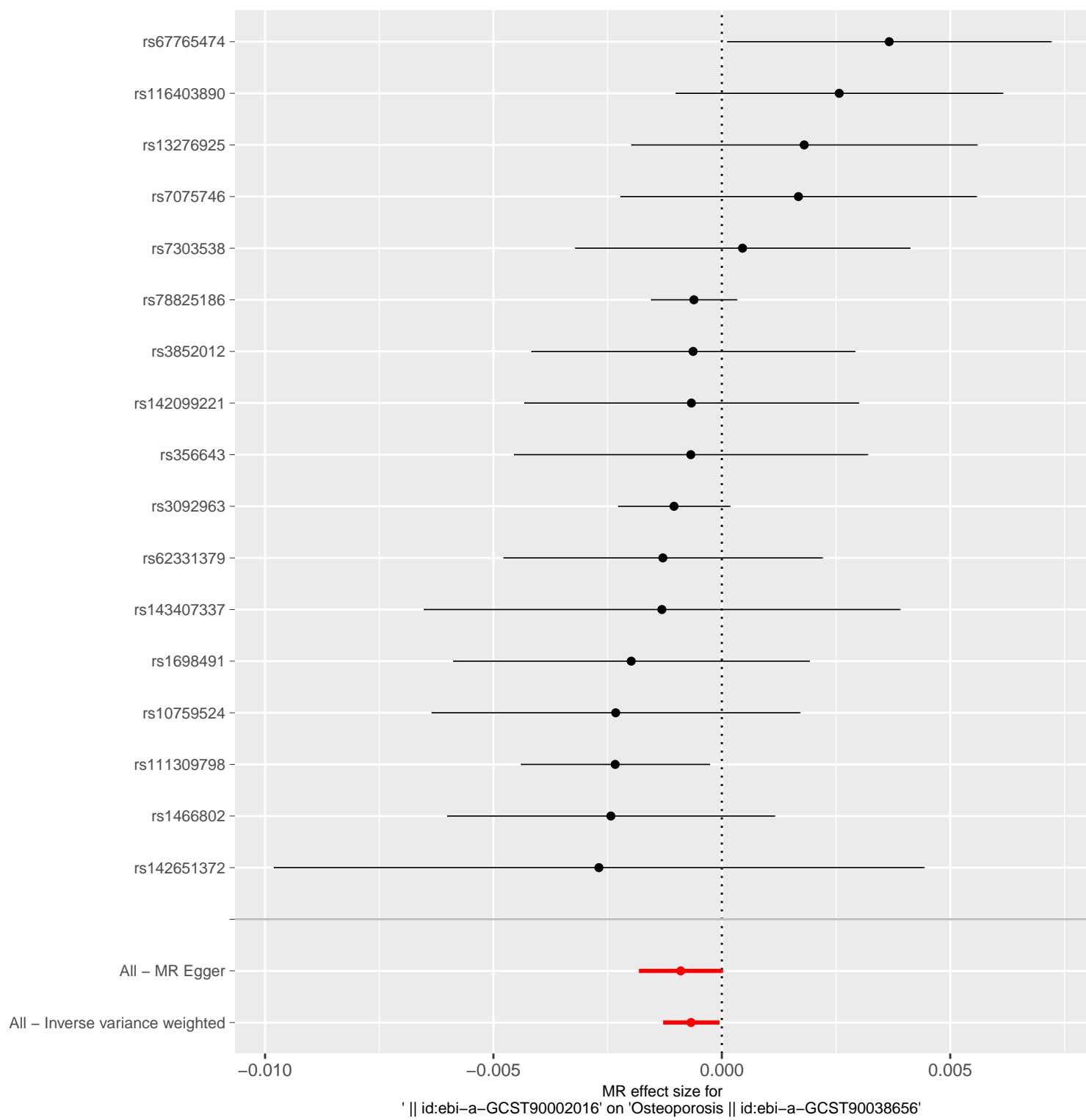

Supplement: Supplementary File 5 — Supplementary figures. [file DataSheet_5.zip › Supplementary file 5/CCR2 on CD62L+ plasmacytoid DC/forest.pdf]

# MR Method

- Inverse variance weighted
- MR Egger

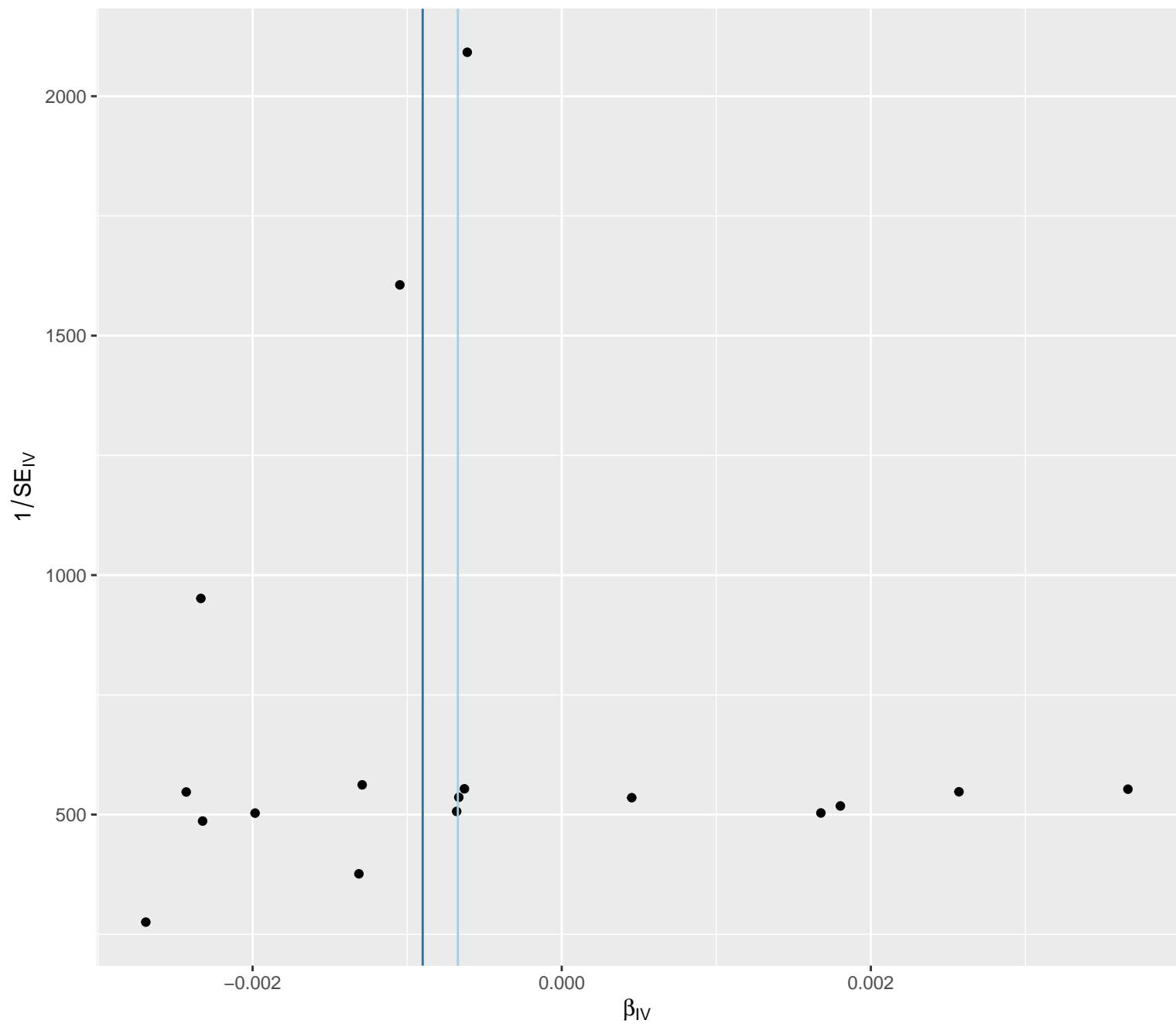

Supplement: Supplementary File 5 — Supplementary figures. [file DataSheet_5.zip › Supplementary file 5/CCR2 on CD62L+ plasmacytoid DC/funnelplot.pdf]

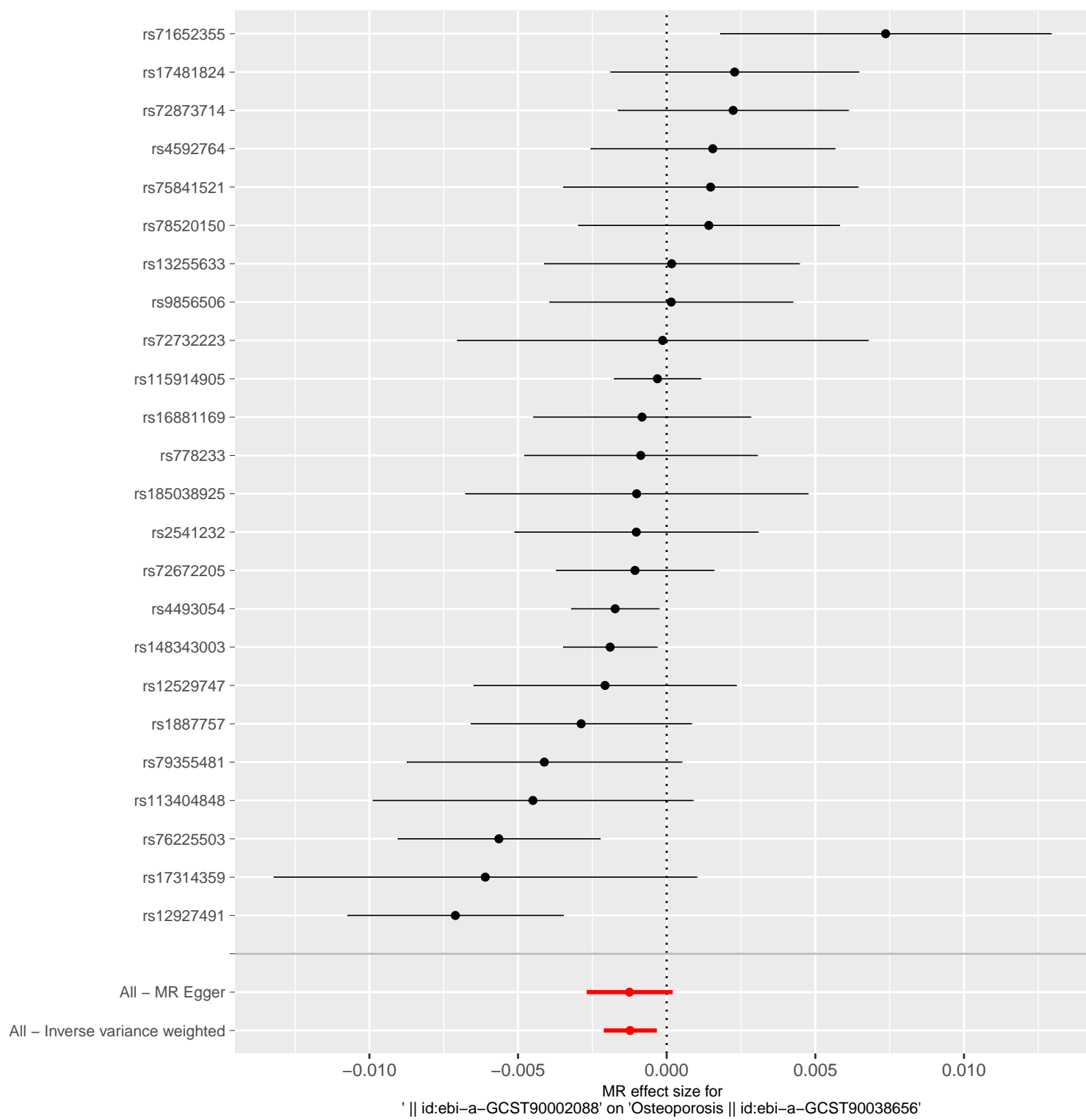

Supplement: Supplementary File 5 — Supplementary figures. [file DataSheet_5.zip › Supplementary file 5/CD11c on CD62L+ myeloid DC/forest.pdf]

# MR Method

- Inverse variance weighted
- MR Egger

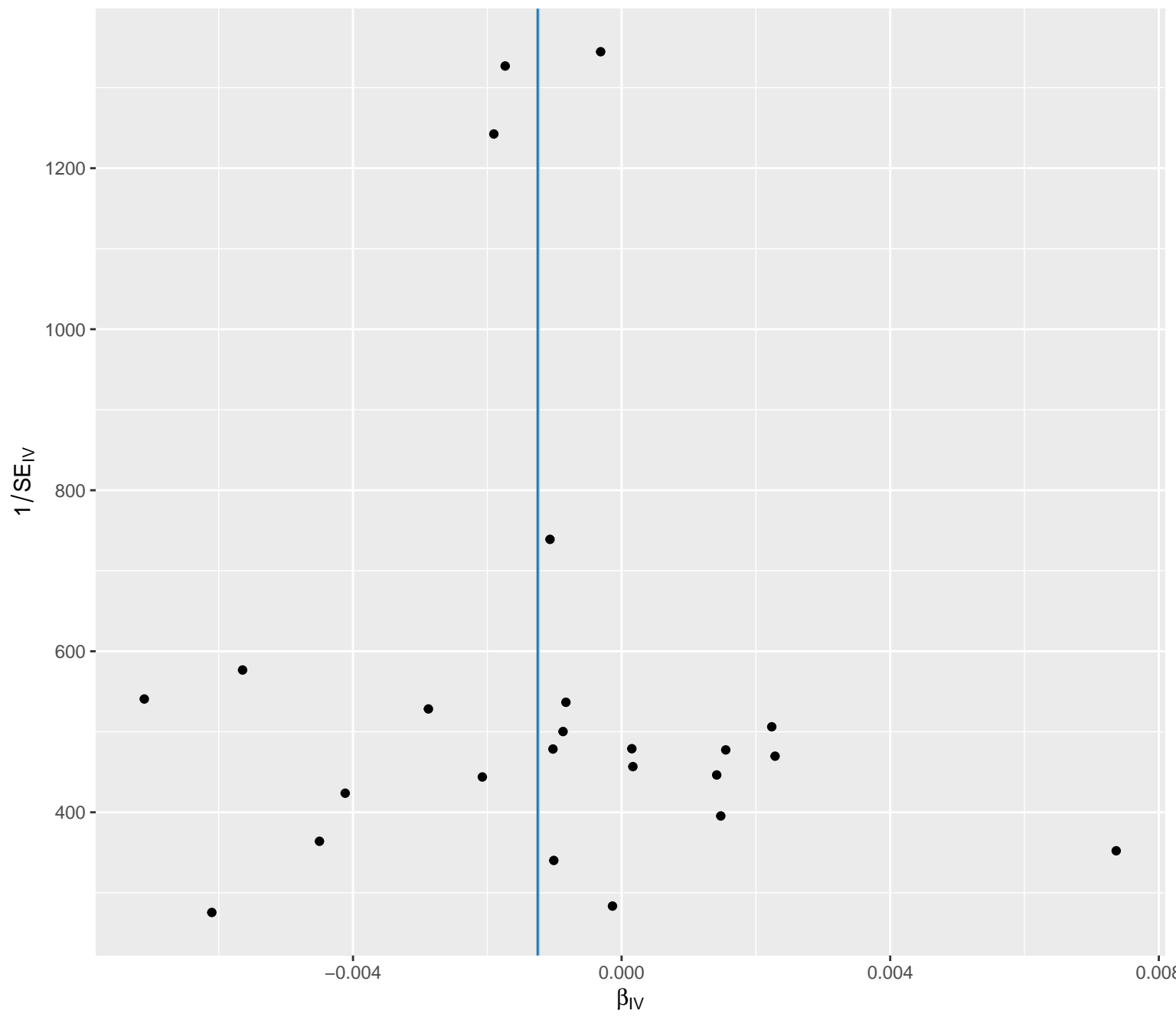

Supplement: Supplementary File 5 — Supplementary figures. [file DataSheet_5.zip › Supplementary file 5/CD11c on CD62L+ myeloid DC/funnelplot.pdf]

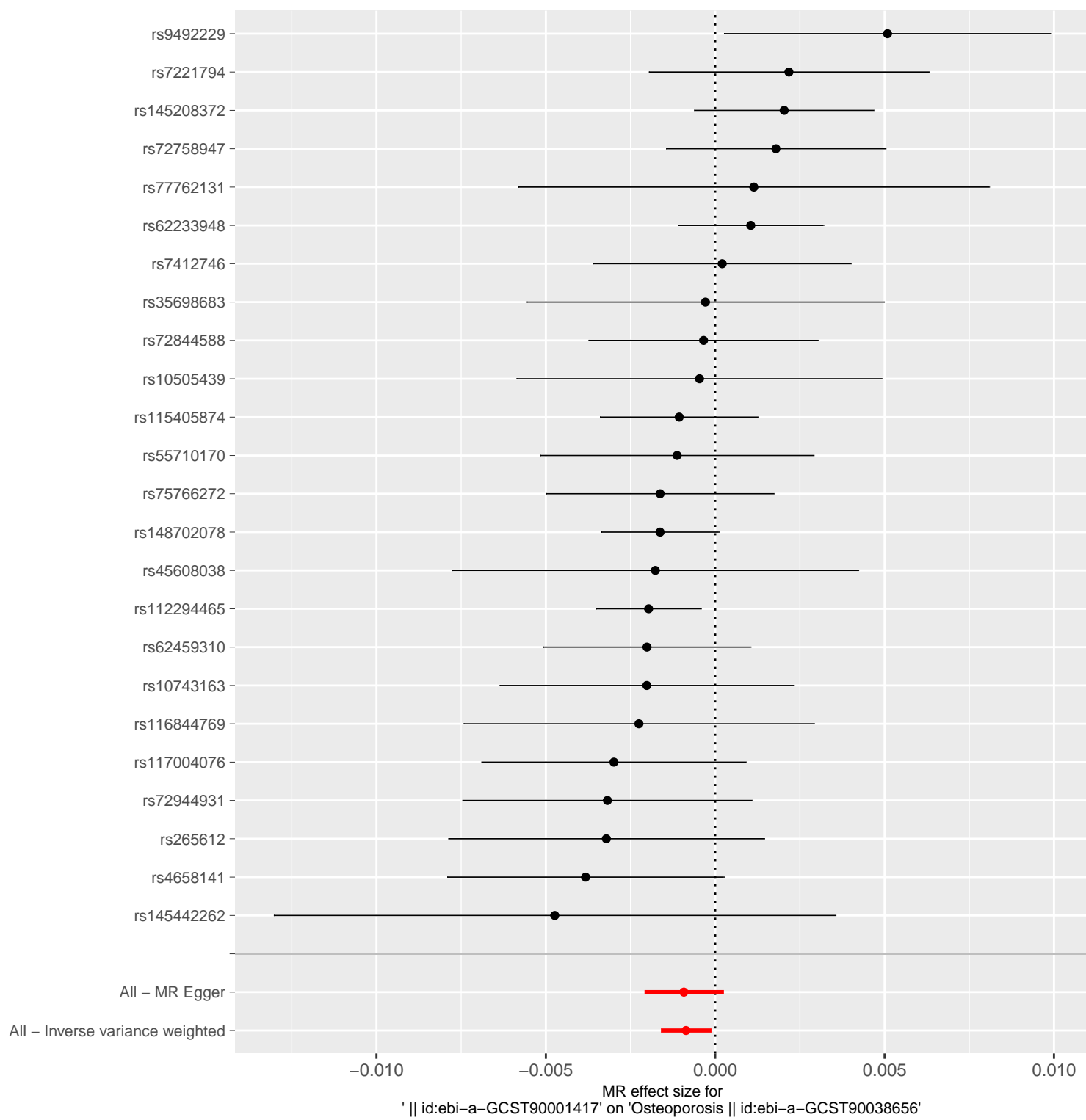

Supplement: Supplementary File 5 — Supplementary figures. [file DataSheet_5.zip › Supplementary file 5/CD24+ CD27+ %B cell/forest.pdf]

# MR Method

- Inverse variance weighted
- MR Egger

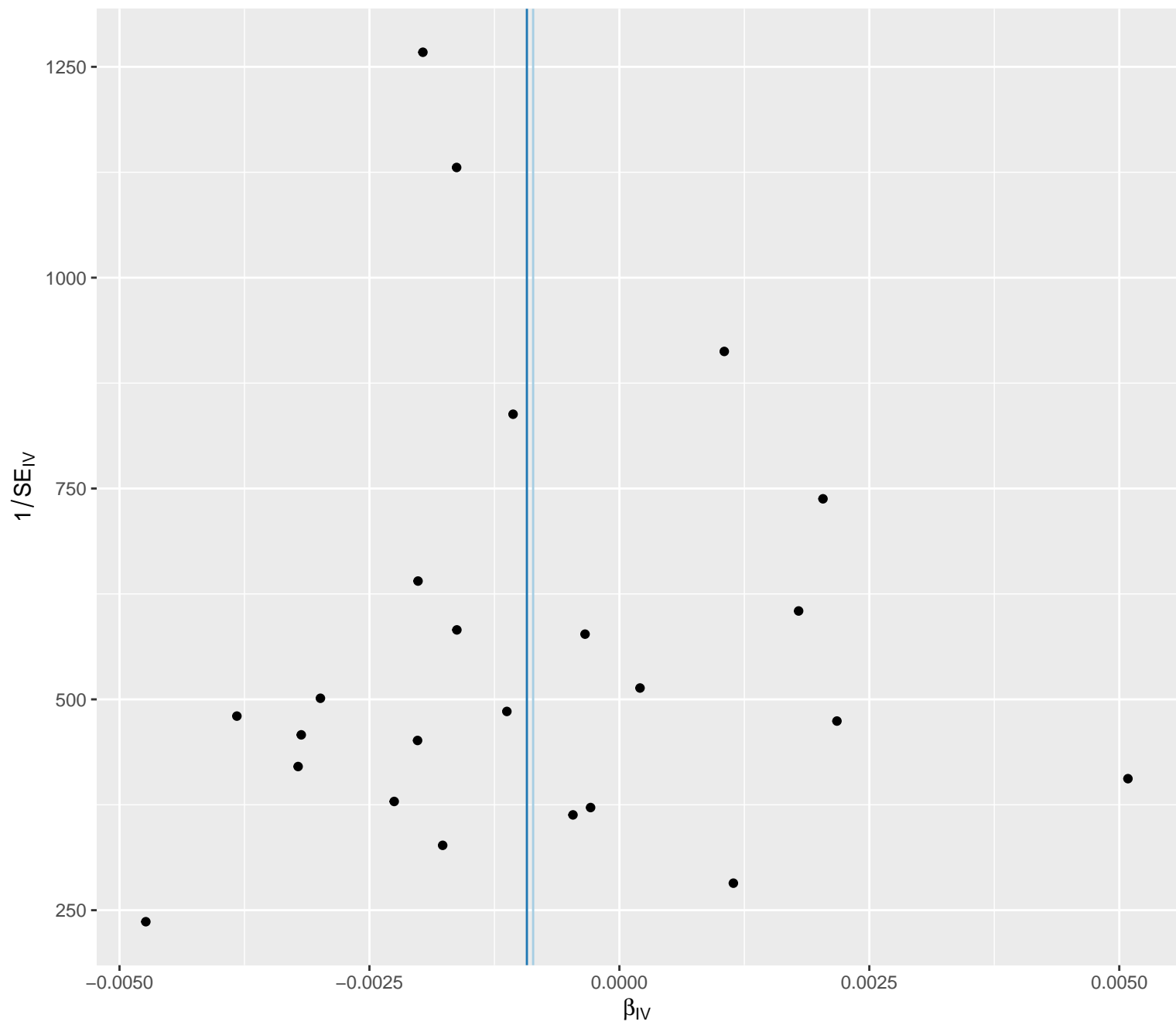

Supplement: Supplementary File 5 — Supplementary figures. [file DataSheet_5.zip › Supplementary file 5/CD24+ CD27+ %B cell/funnelplot.pdf]

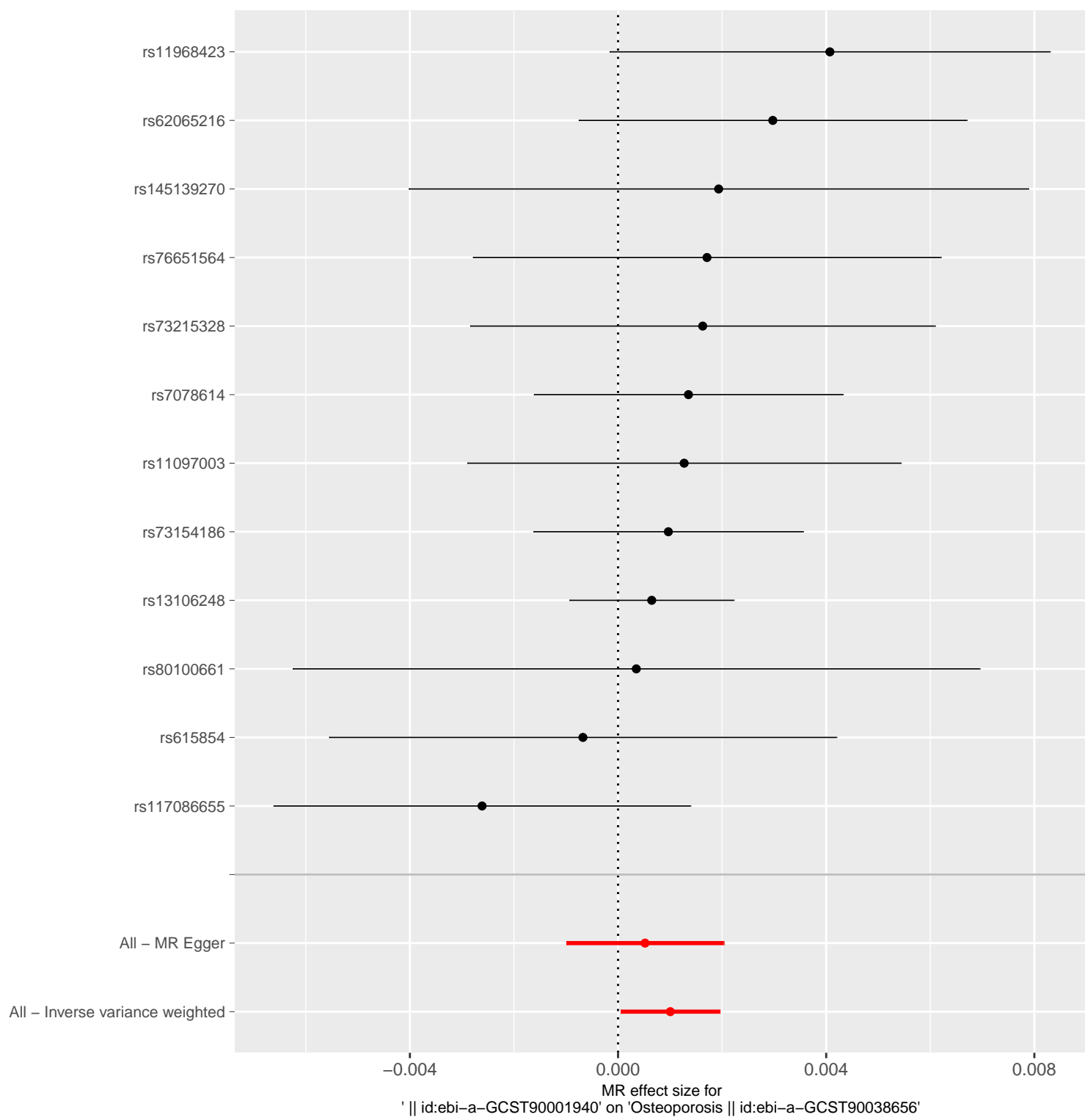

Supplement: Supplementary File 5 — Supplementary figures. [file DataSheet_5.zip › Supplementary file 5/CD25 on CD39+ activated Tregú¿riskú⌐/forest.pdf]

# MR Method

- Inverse variance weighted
- MR Egger

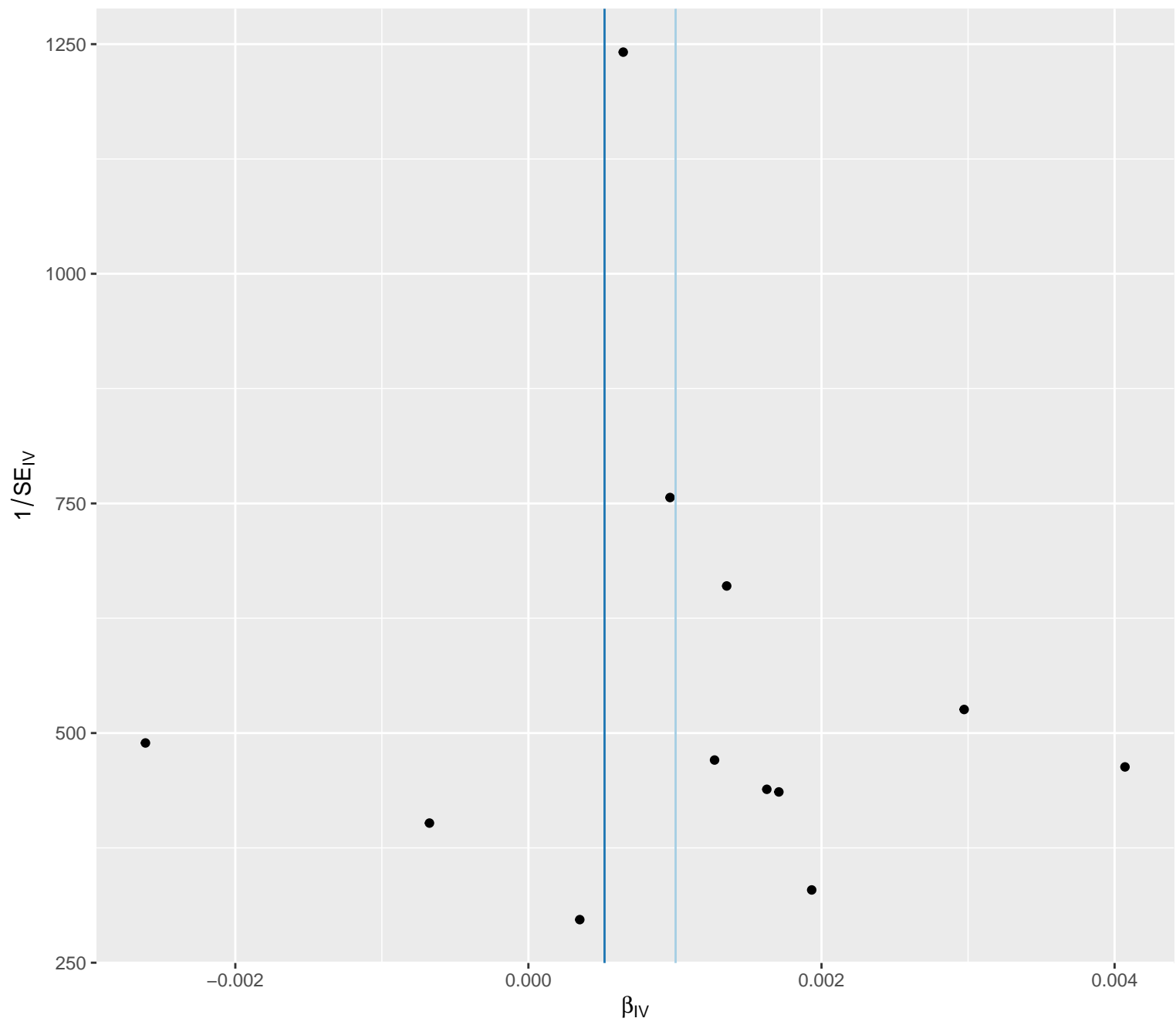

Supplement: Supplementary File 5 — Supplementary figures. [file DataSheet_5.zip › Supplementary file 5/CD25 on CD39+ activated Tregú¿riskú⌐/funnelplot.pdf]

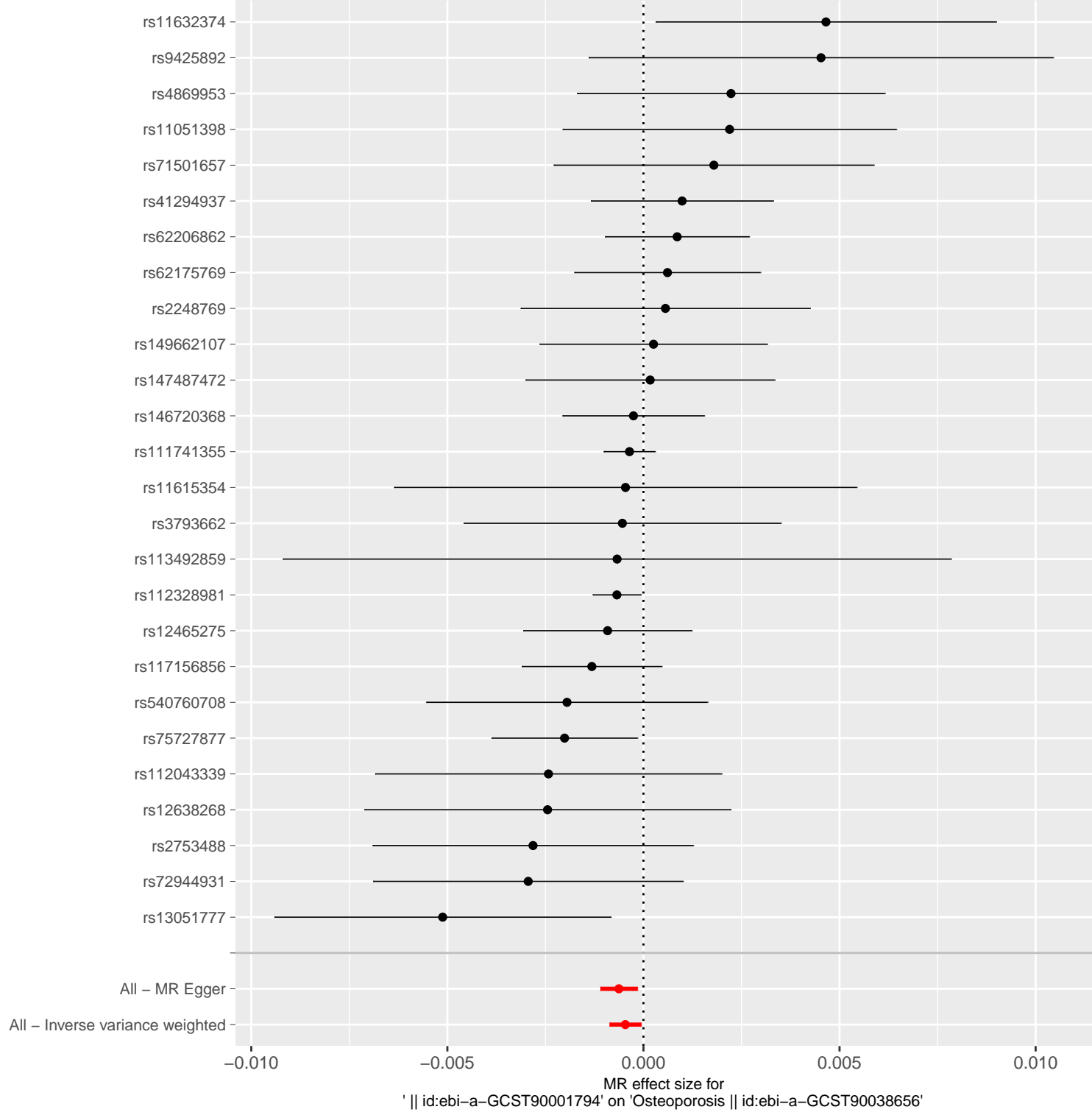

Supplement: Supplementary File 5 — Supplementary figures. [file DataSheet_5.zip › Supplementary file 5/CD25 on IgD+/forest.pdf]

# MR Method

- Inverse variance weighted
- MR Egger

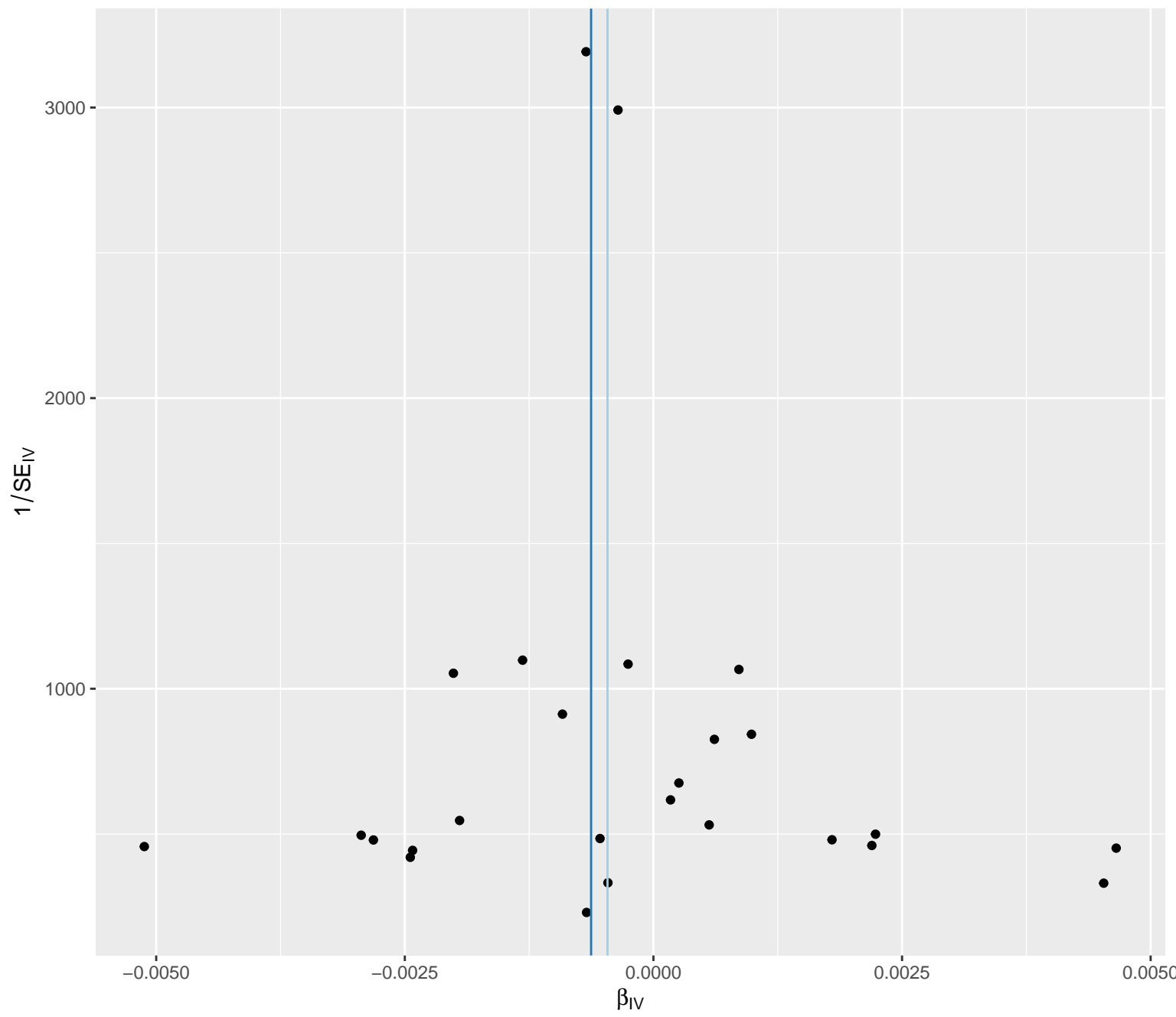

Supplement: Supplementary File 5 — Supplementary figures. [file DataSheet_5.zip › Supplementary file 5/CD25 on IgD+/funnelplot.pdf]

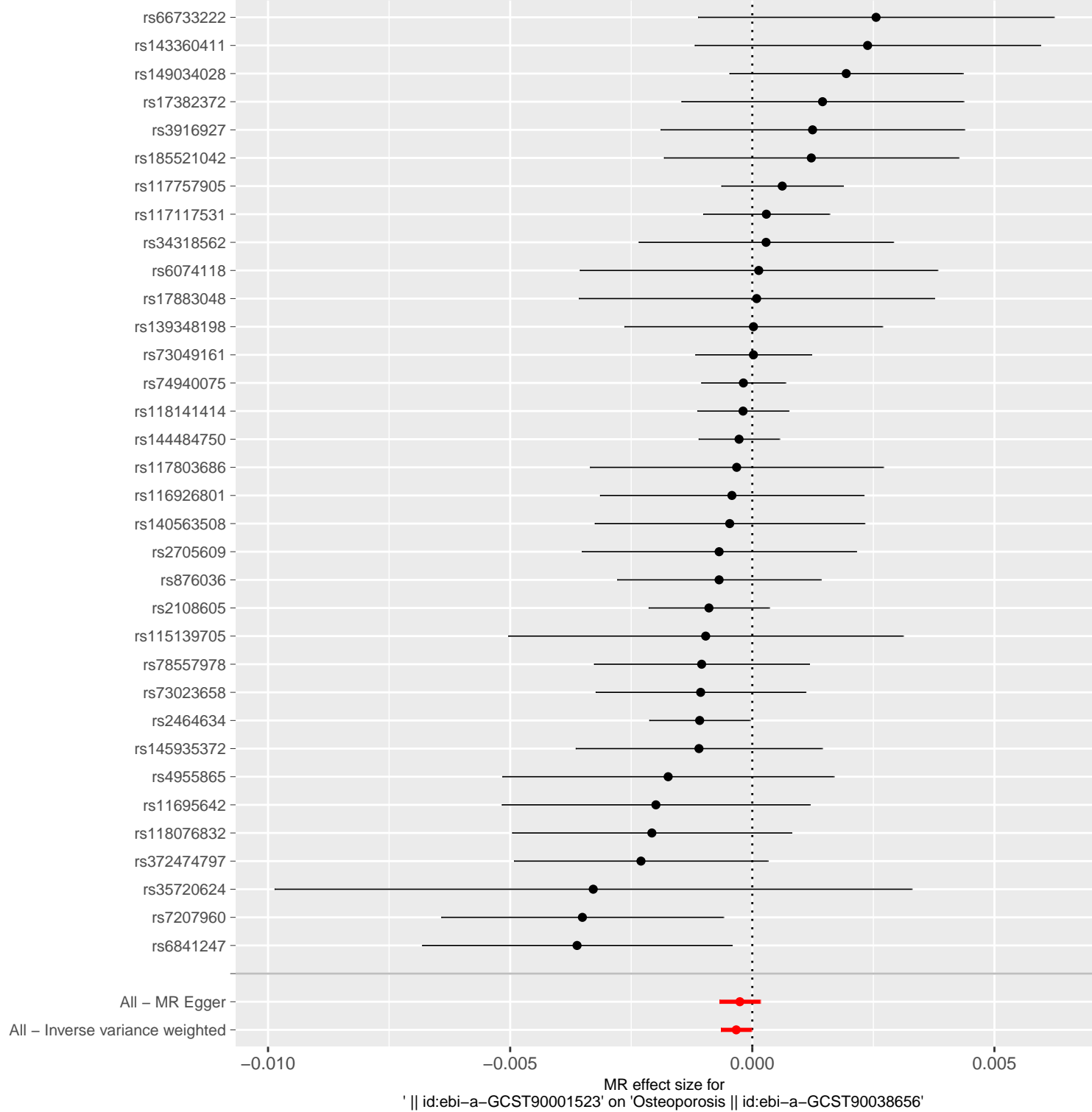

Supplement: Supplementary File 5 — Supplementary figures. [file DataSheet_5.zip › Supplementary file 5/CD33- HLA DR+ AC/forest.pdf]

# MR Method

- Inverse variance weighted
- MR Egger

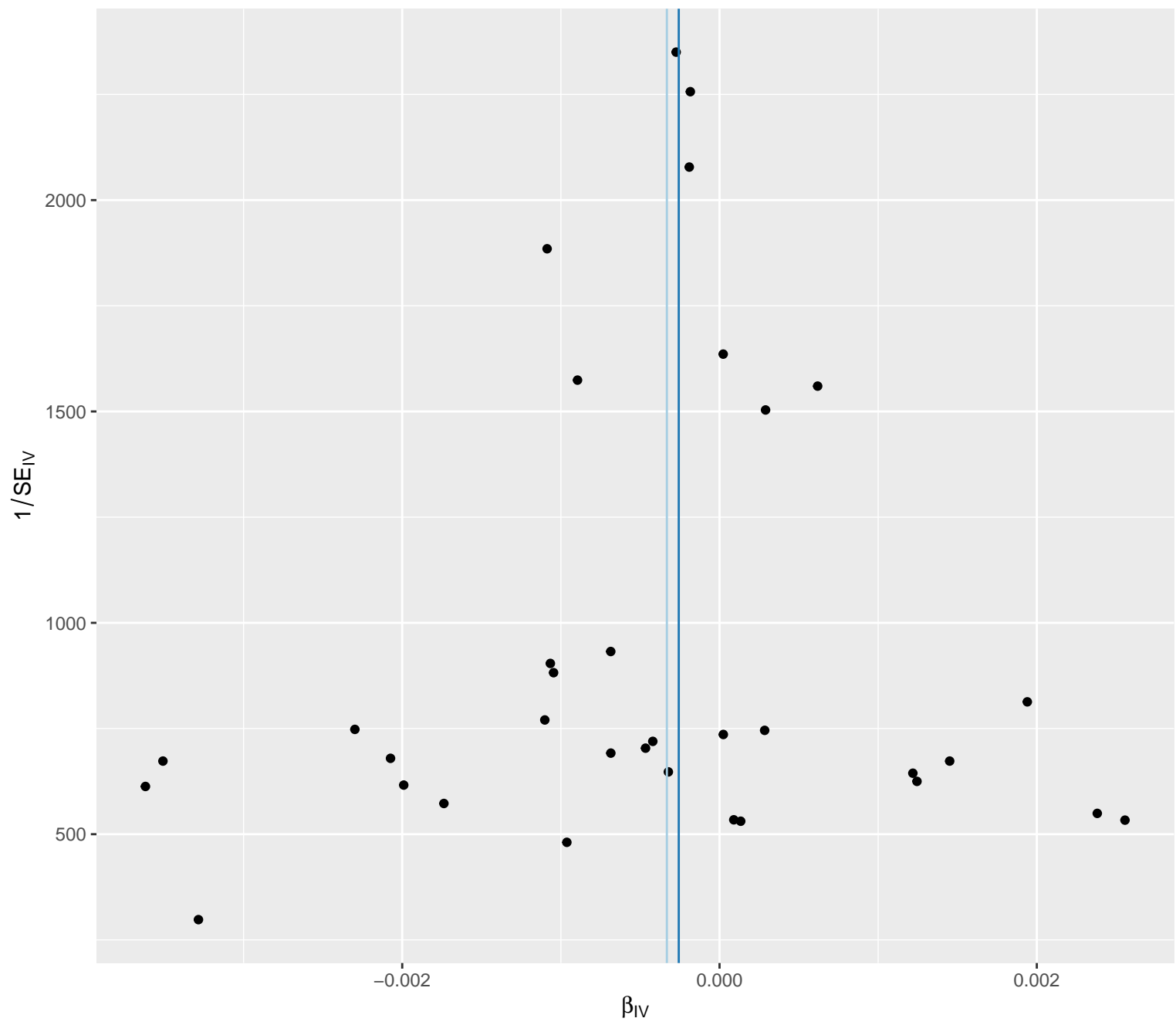

Supplement: Supplementary File 5 — Supplementary figures. [file DataSheet_5.zip › Supplementary file 5/CD33- HLA DR+ AC/funnelplot.pdf]

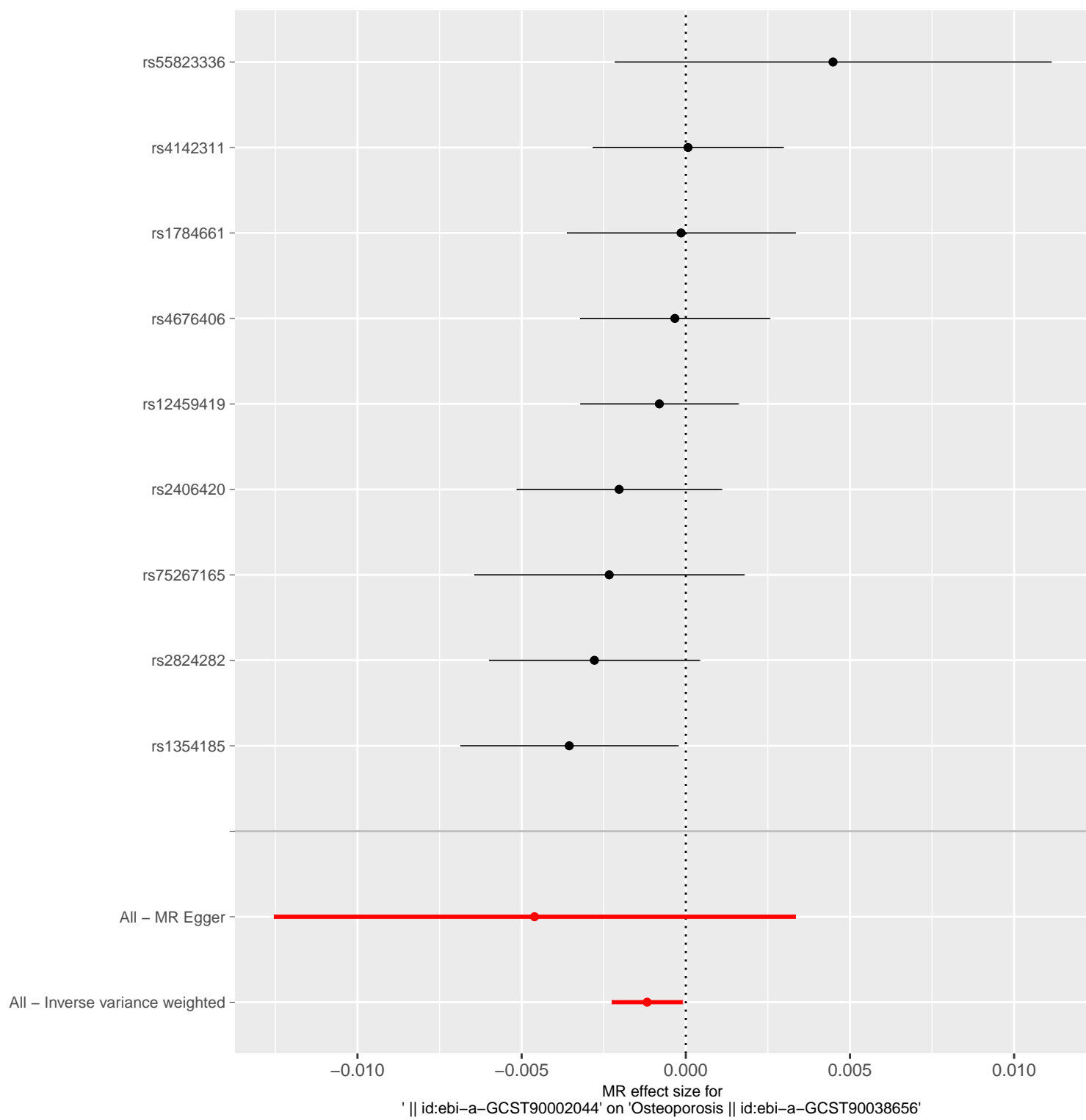

Supplement: Supplementary File 5 — Supplementary figures. [file DataSheet_5.zip › Supplementary file 5/CD45 on CD33dim HLA DR+ CD11b-/forest.pdf]

# MR Method

- Inverse variance weighted
- MR Egger

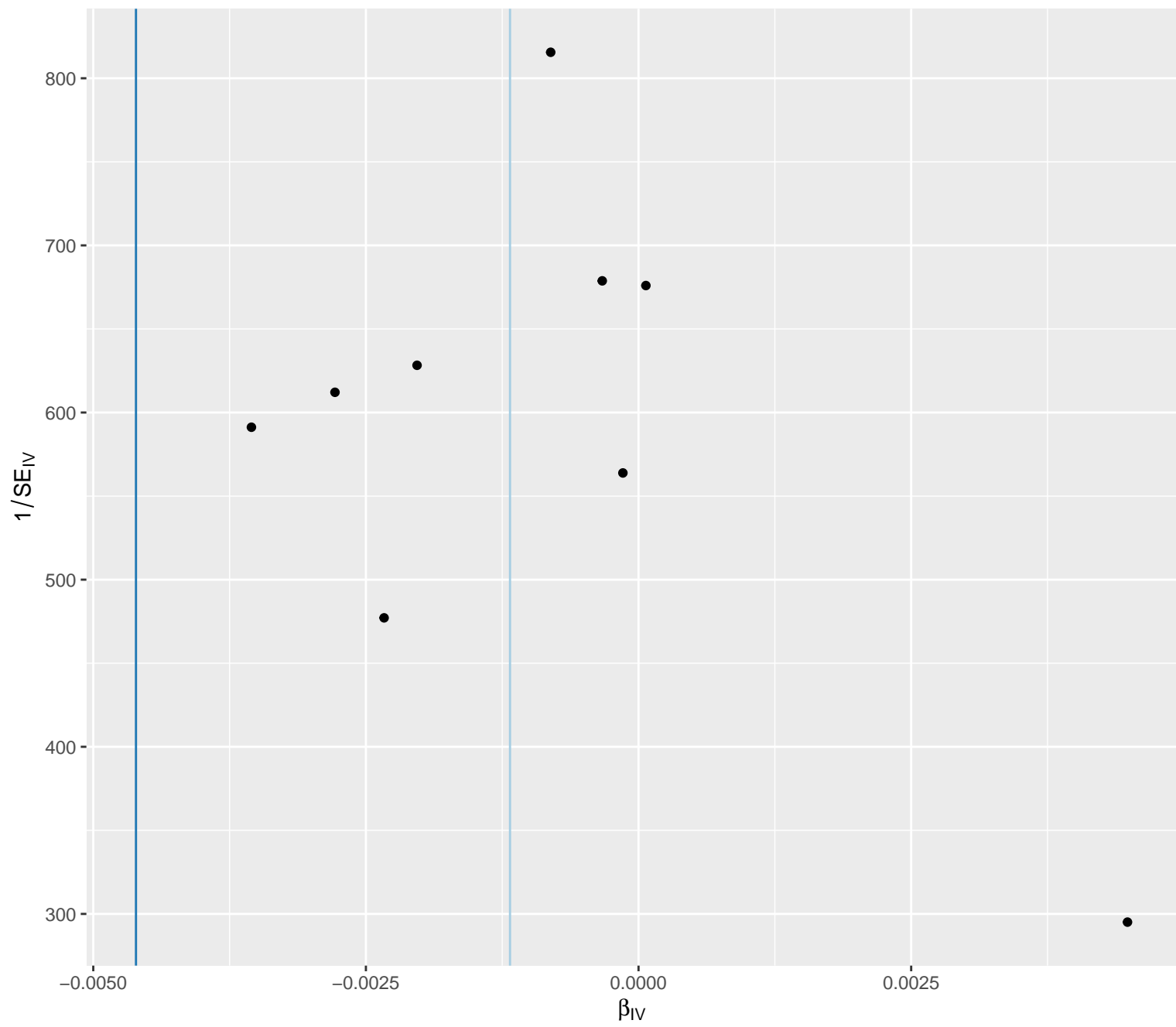

Supplement: Supplementary File 5 — Supplementary figures. [file DataSheet_5.zip › Supplementary file 5/CD45 on CD33dim HLA DR+ CD11b-/funnelplot.pdf]

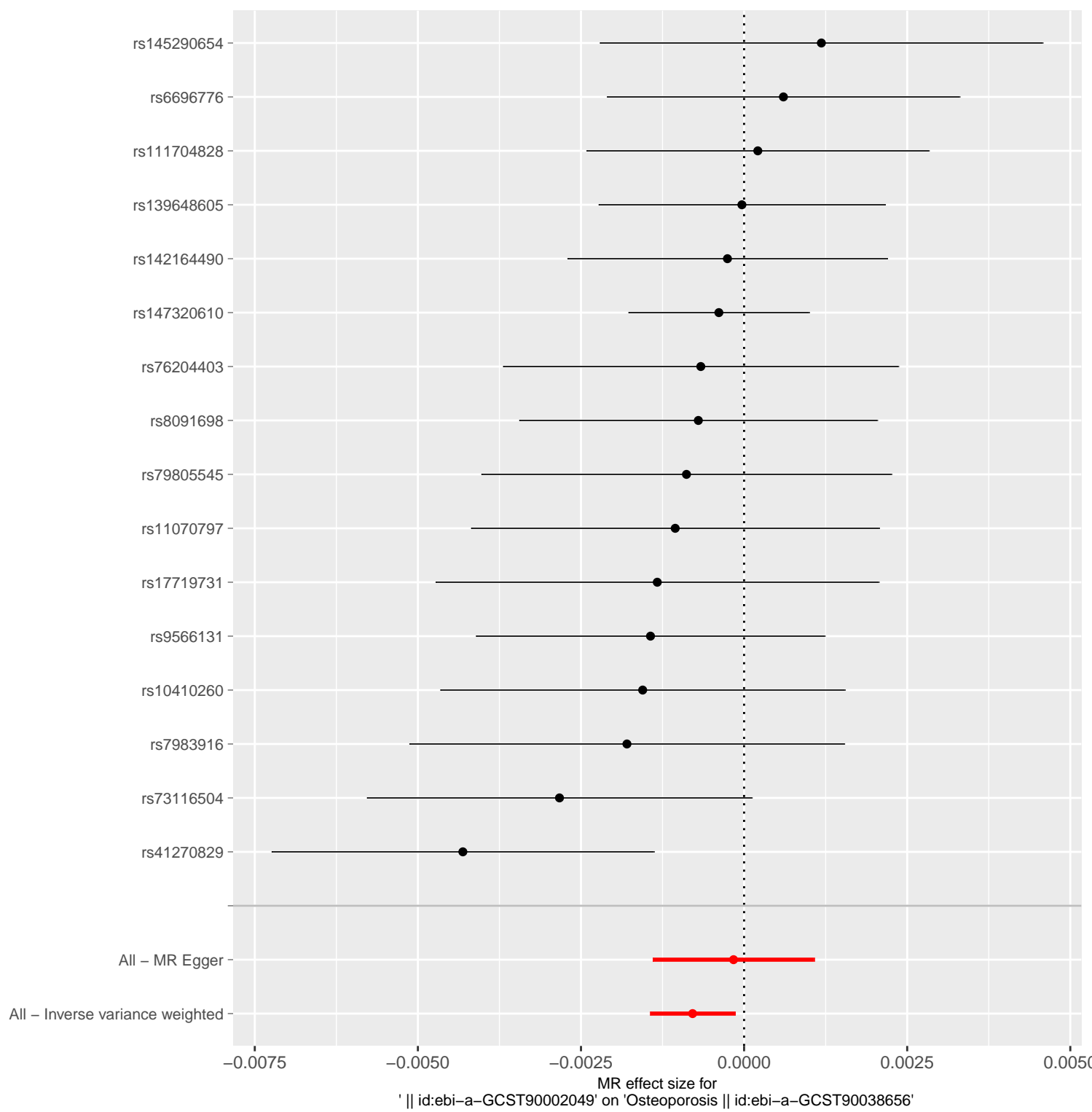

Supplement: Supplementary File 5 — Supplementary figures. [file DataSheet_5.zip › Supplementary file 5/CD45 on Mo MDSC/forest.pdf]

# MR Method

- Inverse variance weighted
- MR Egger

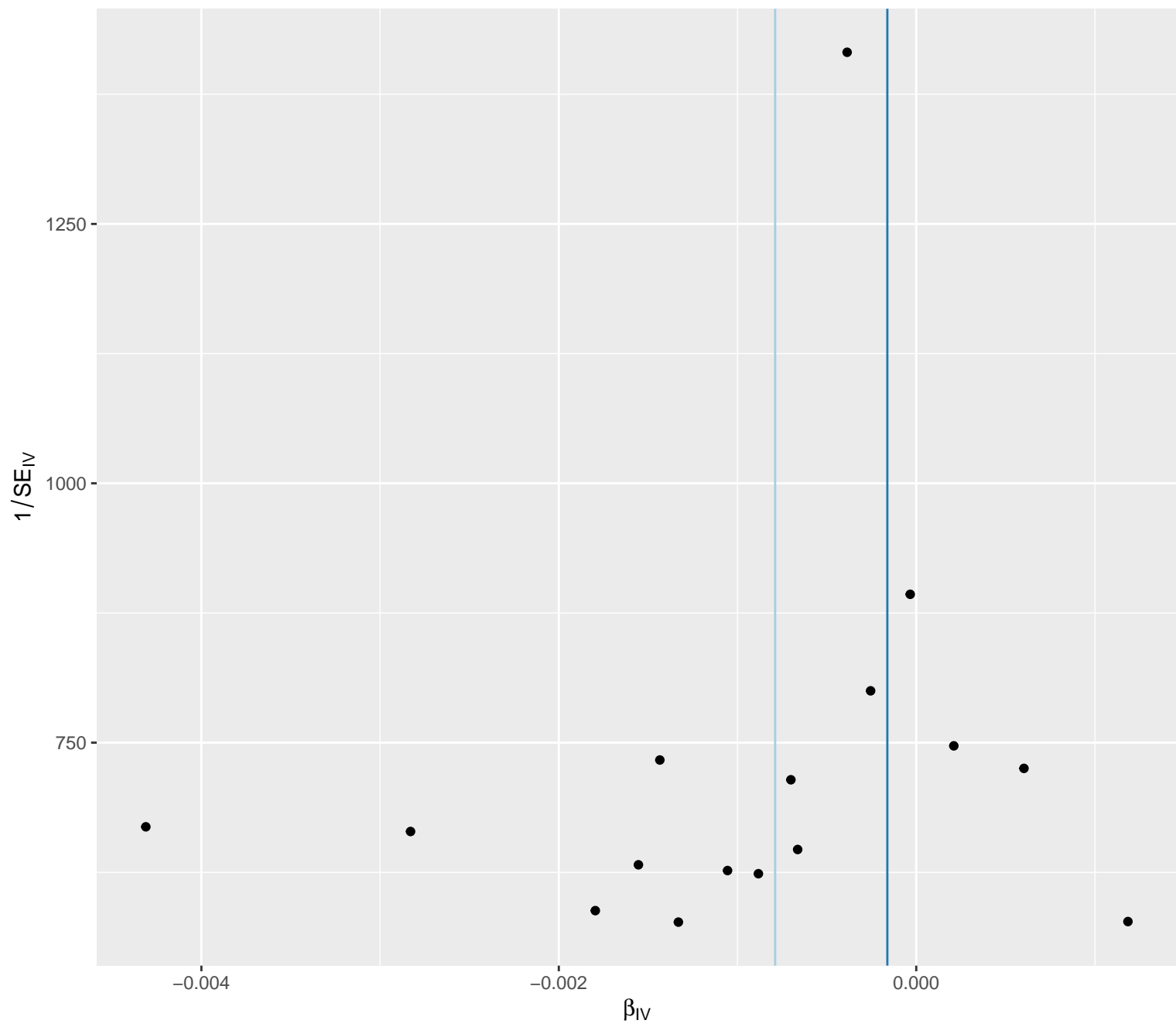

Supplement: Supplementary File 5 — Supplementary figures. [file DataSheet_5.zip › Supplementary file 5/CD45 on Mo MDSC/funnelplot.pdf]

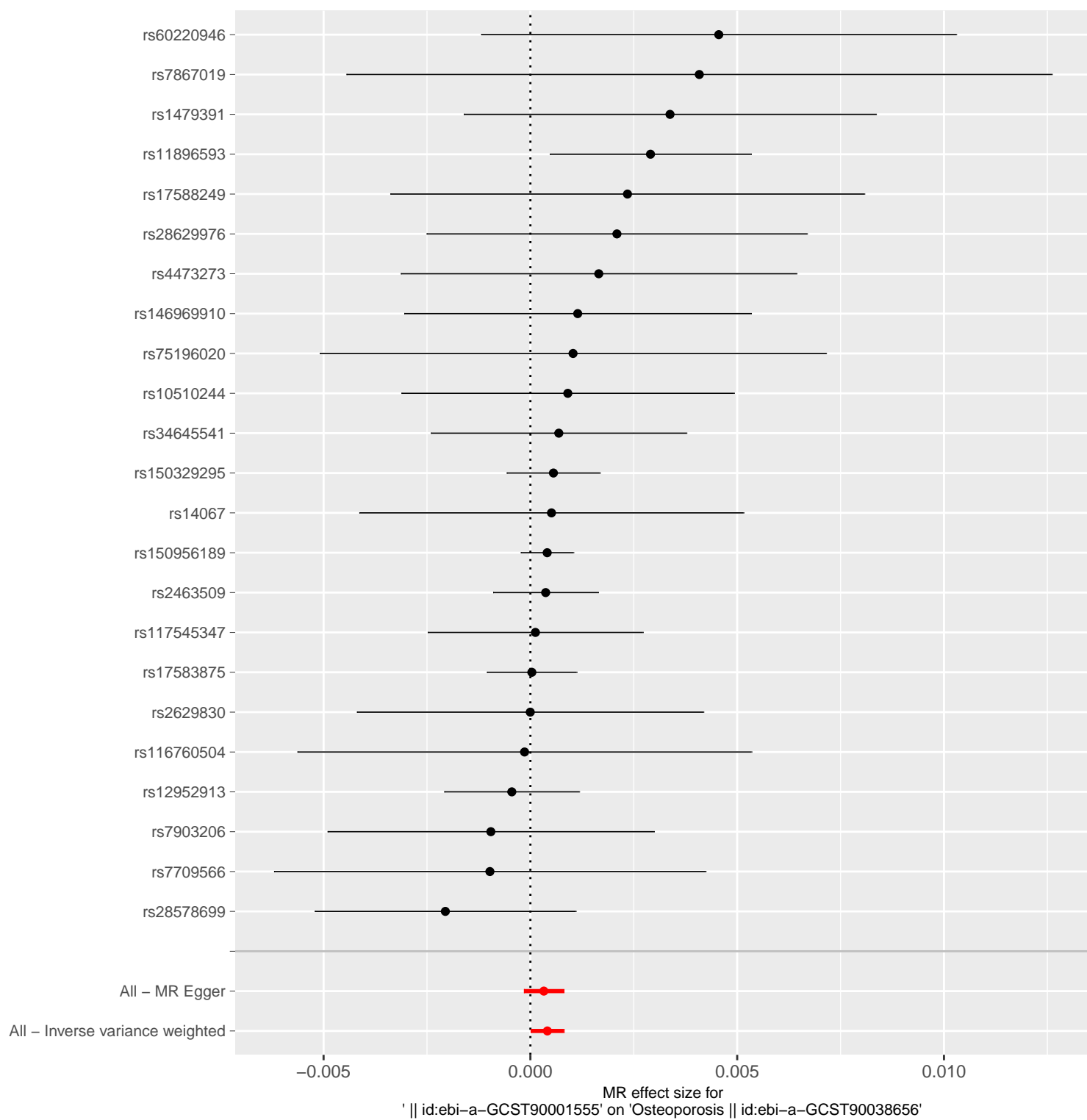

Supplement: Supplementary File 5 — Supplementary figures. [file DataSheet_5.zip › Supplementary file 5/EM CD8br %CD8brú¿riskú⌐/forest.pdf]

# MR Method

- Inverse variance weighted
- MR Egger

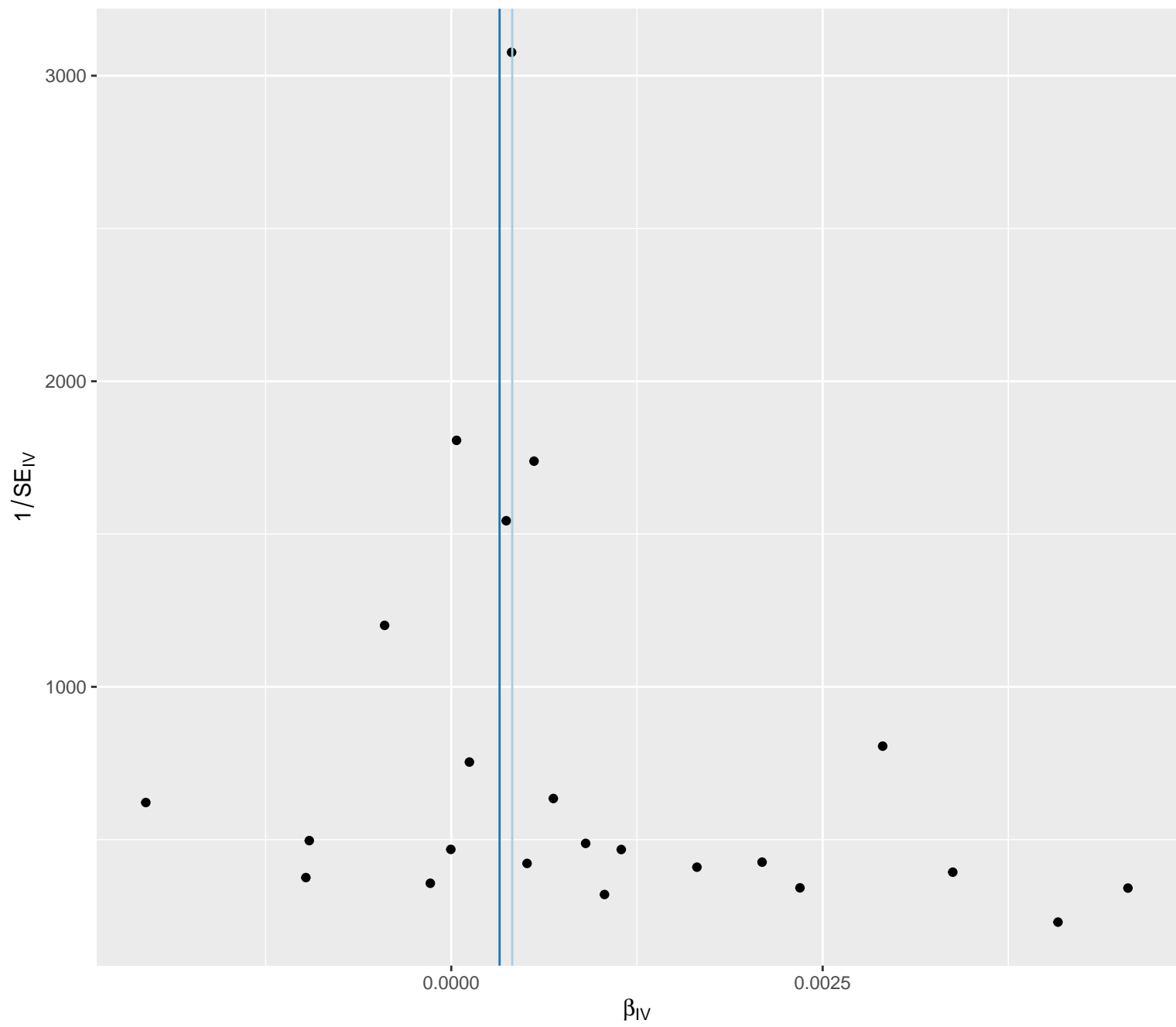

Supplement: Supplementary File 5 — Supplementary figures. [file DataSheet_5.zip › Supplementary file 5/EM CD8br %CD8brú¿riskú⌐/funnelplot.pdf]

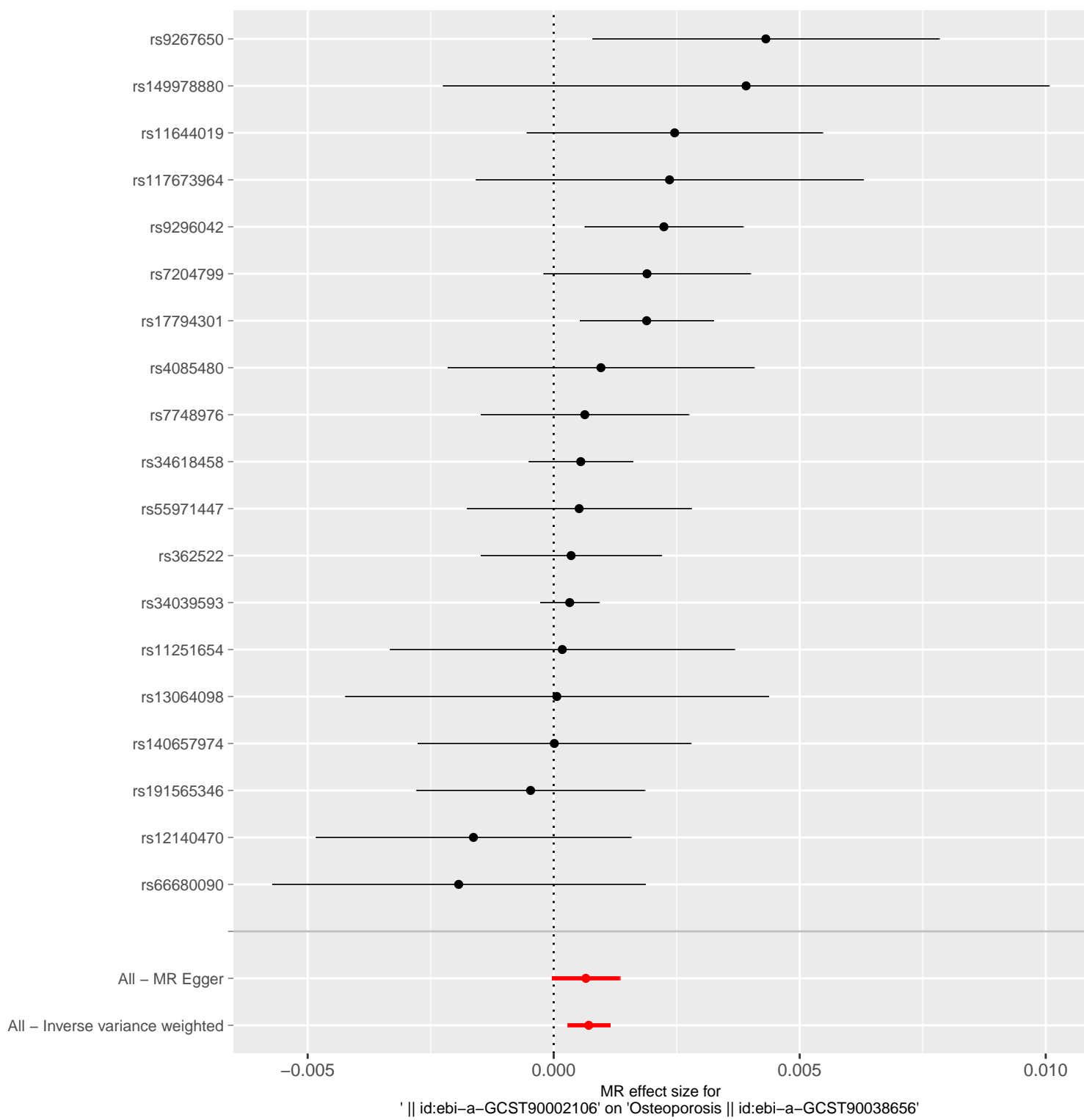

Supplement: Supplementary File 5 — Supplementary figures. [file DataSheet_5.zip › Supplementary file 5/HLA DR on DCú¿riskú⌐/forest.pdf]

# MR Method

- Inverse variance weighted
- MR Egger

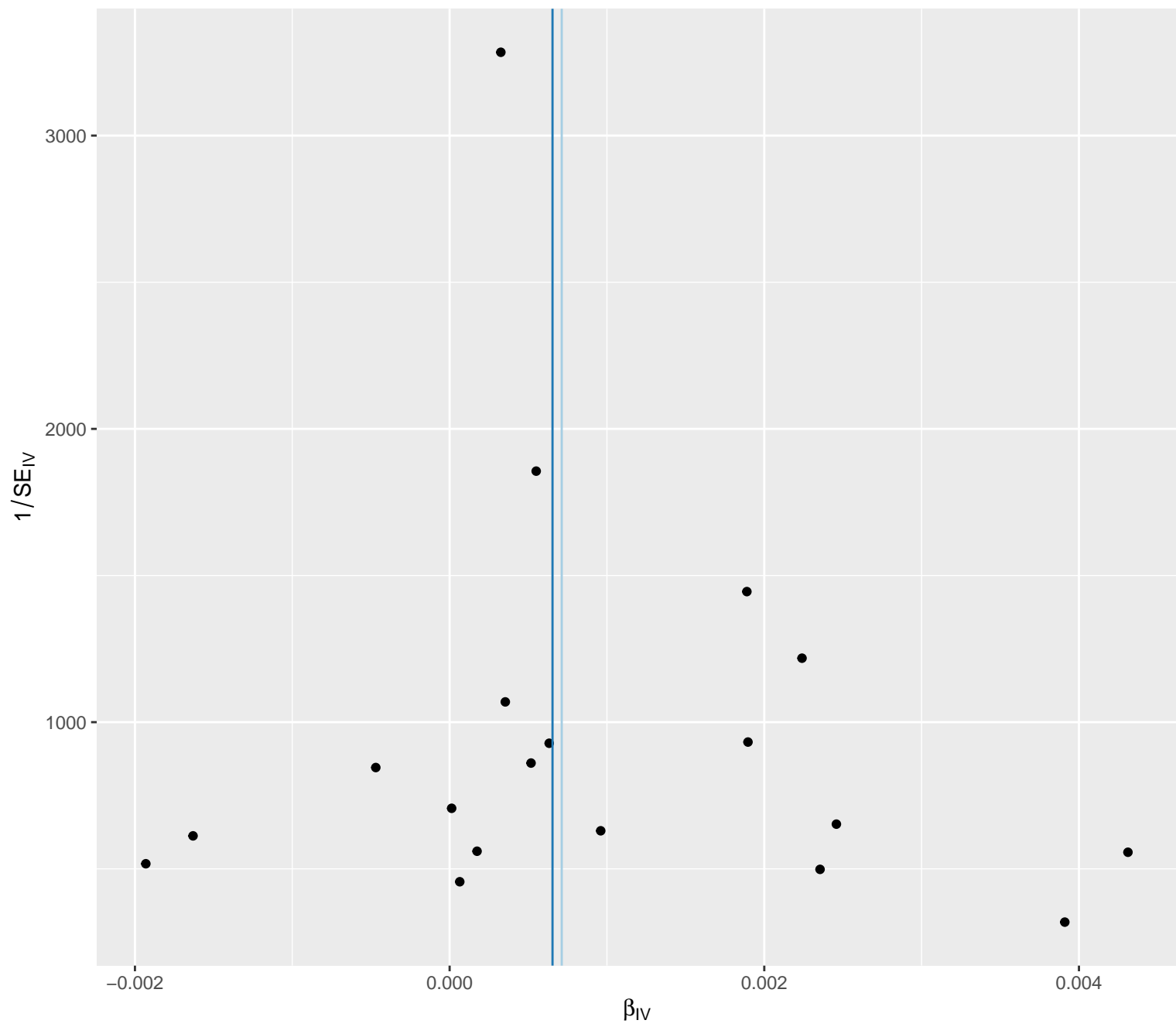

Supplement: Supplementary File 5 — Supplementary figures. [file DataSheet_5.zip › Supplementary file 5/HLA DR on DCú¿riskú⌐/funnelplot.pdf]

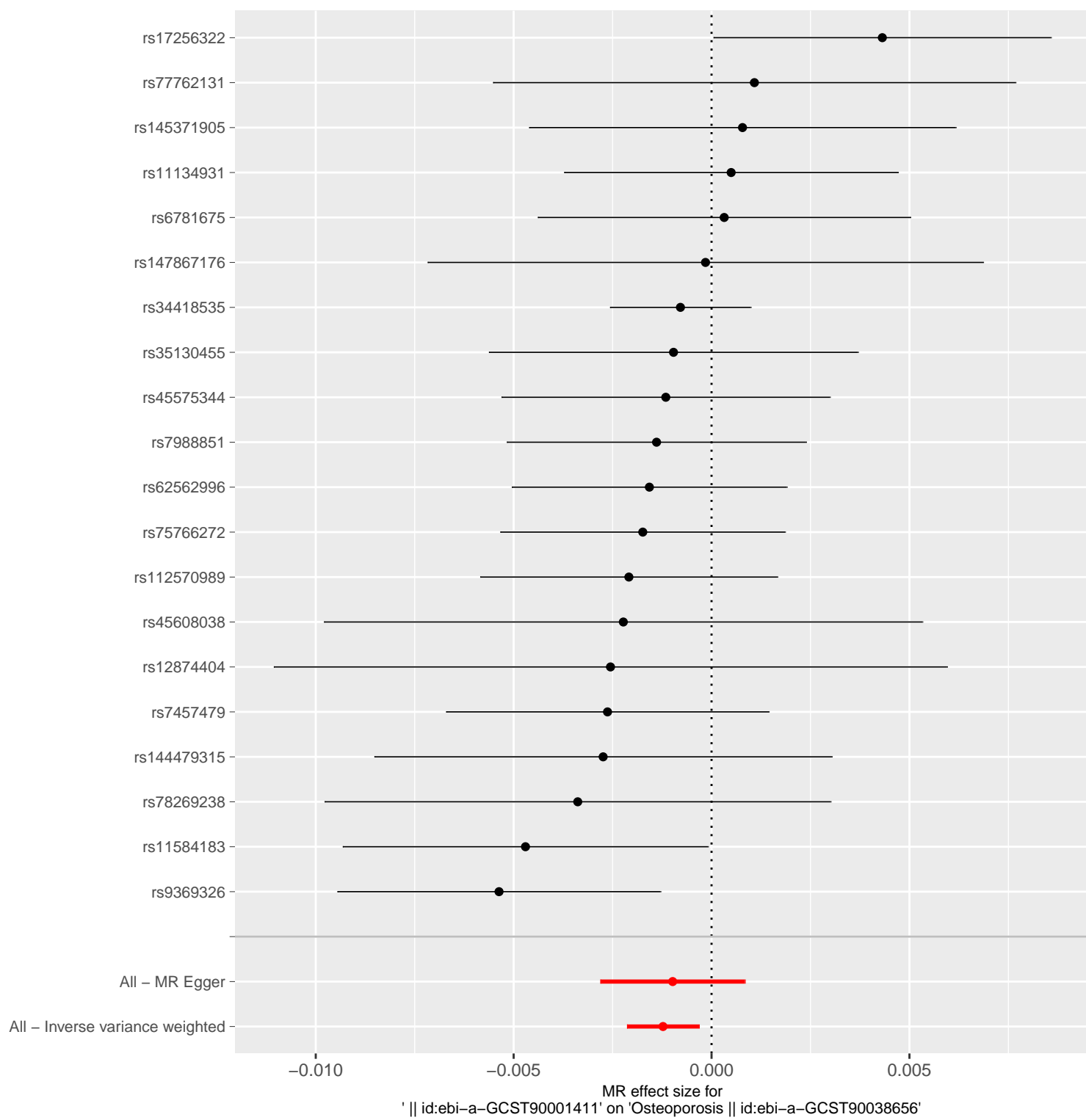

Supplement: Supplementary File 5 — Supplementary figures. [file DataSheet_5.zip › Supplementary file 5/IgD+ CD24+ %B cell/forest.pdf]

# MR Method

- Inverse variance weighted
- MR Egger

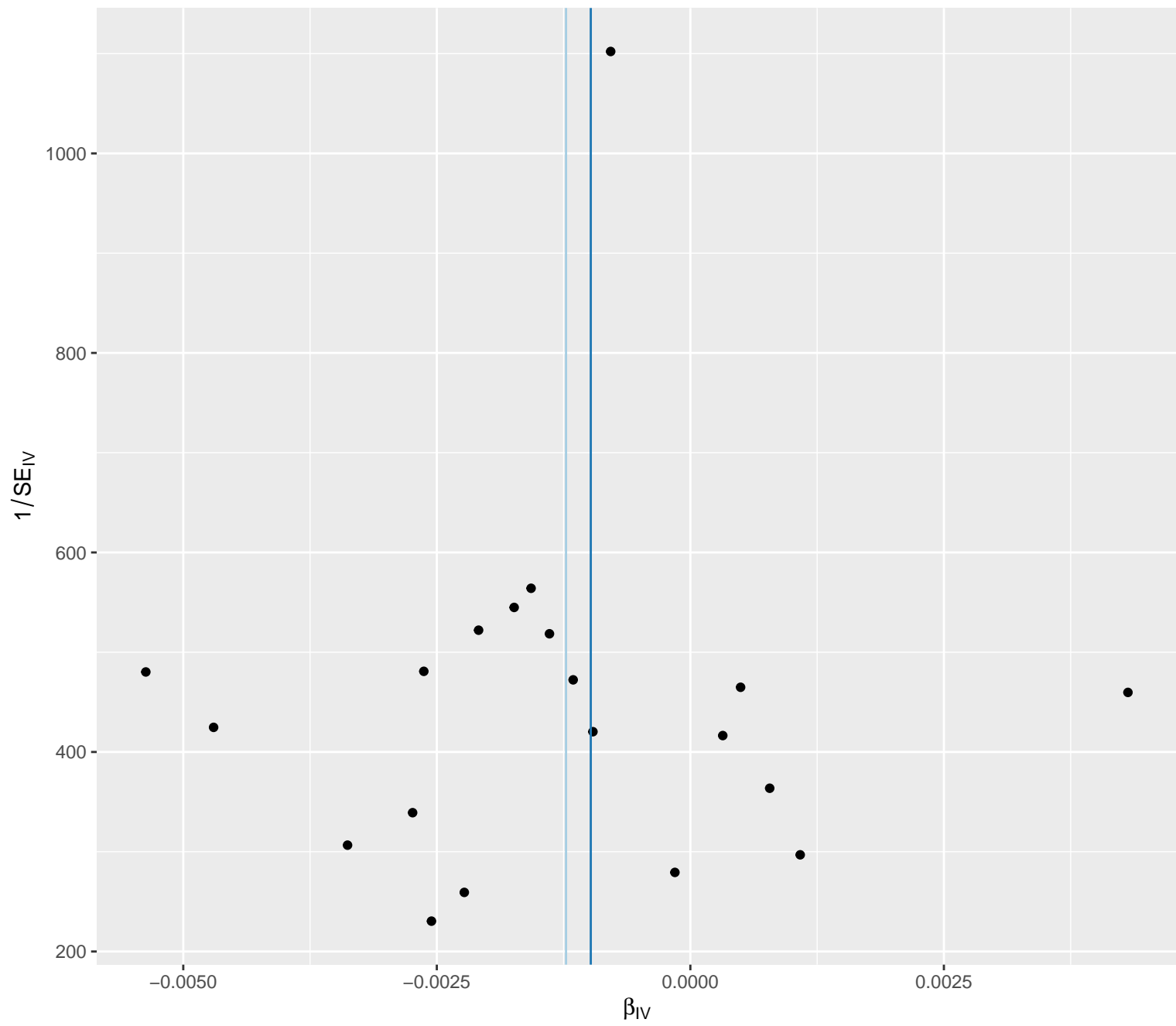

Supplement: Supplementary File 5 — Supplementary figures. [file DataSheet_5.zip › Supplementary file 5/IgD+ CD24+ %B cell/funnelplot.pdf]

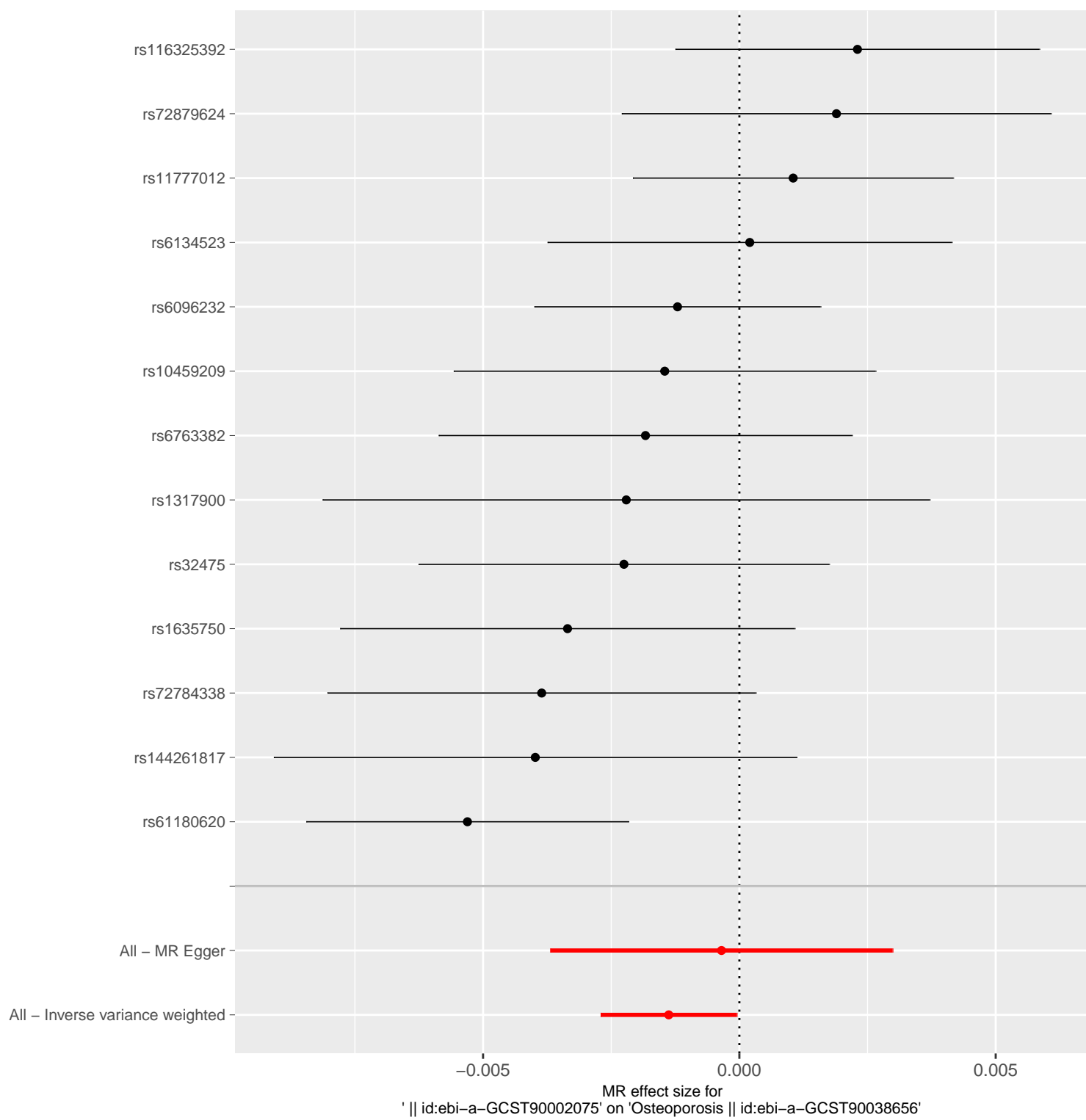

Supplement: Supplementary File 5 — Supplementary figures. [file DataSheet_5.zip › Supplementary file 5/SSC-A on B cell/forest.pdf]

# MR Method

- Inverse variance weighted
- MR Egger

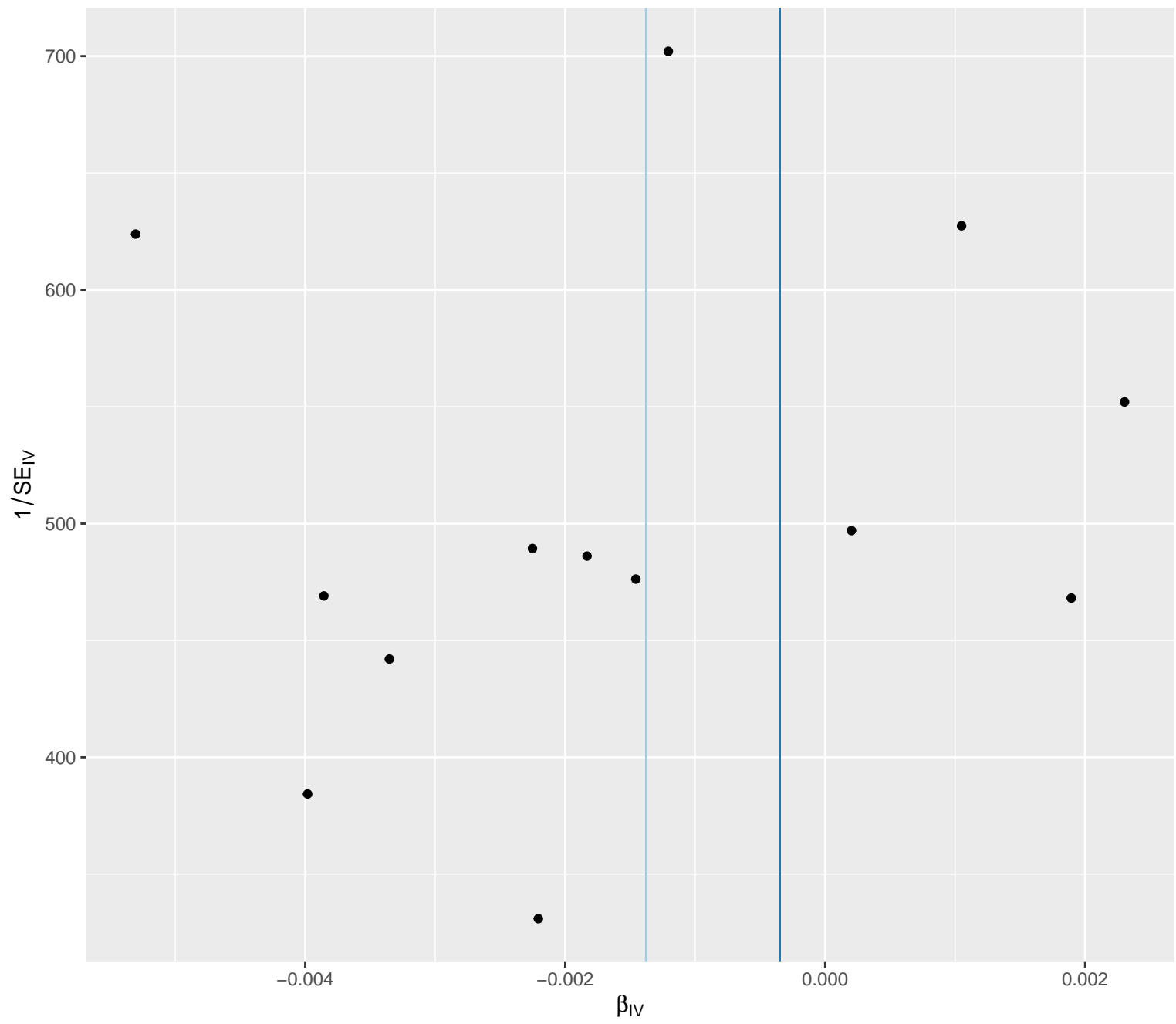

Supplement: Supplementary File 5 — Supplementary figures. [file DataSheet_5.zip › Supplementary file 5/SSC-A on B cell/funnelplot.pdf]
